# Evaluating combined acupuncture and antiresorptive therapy in Chinese women with postmenopausal osteoporosis: a systematic review and network meta-analysis

**DOI:** 10.3389/fendo.2026.1784394

**Published:** 2026-07-01

**Authors:** Jiamei Zhuang, Dan Su, Qinlong Gao, Lingsan Hu, Yue Jiang, Guangbin Yu, Xiong Chen, Dan Xi

**Affiliations:** 1 The Seventh Clinical College of Guangzhou University of Chinese Medicine, Shenzhen Bao’an Chinese Medicine Hospital, Guangzhou University of Chinese Medicine, Shenzhen, Guangdong, China; 2 Clinical Medical College of Acupuncture Moxibustion and Rehabilitation, Guangzhou University of Chinese Medicine, Guangzhou, Guangdong, China

**Keywords:** acupuncture, antiresorptive therapy, network meta-analysis, personalized treatment, postmenopausal osteoporosis

## Abstract

**Background:**

Postmenopausal osteoporosis (PMOP) is a common metabolic disorder in middle−aged and older women, leading to fractures, disability, and increased mortality. Current treatments include bisphosphonates (BPs), calcitonin (CT), vitamin D (VD), and acupuncture (Acu), often used in combination, but comparative evidence on different combined strategies remain limited.

**Objective:**

To systematically compare the efficacy and safety of acupuncture combined with various medications for PMOP, supporting evidence−based clinical decisions.

**Methods:**

We searched eight databases from inception to April 11, 2026 for randomized controlled trials (RCTs) evaluating acupuncture plus pharmacotherapy for PMOP. Primary outcomes included lumbar spine (LS) and femoral neck (FN) bone mineral density (BMD), clinical efficacy, Traditional Chinese Medicine (TCM) syndrome scores, and Visual Analog Scale (VAS) pain scores. Secondary outcomes were procollagen type I N−terminal propeptide (PINP), C−terminal telopeptide (CTX), estradiol (E2), alkaline phosphatase (ALP), and osteocalcin (OCN). A Bayesian network meta−analysis was performed, ranking treatments by surface under the cumulative ranking curve (SUCRA).

**Results:**

We included 112 RCTs (9, 908 patients, 83 treatment strategies). For LS−BMD, Calcitriol (Cal)_Electroacupuncture (ElecAcu) ranked highest. For FN−BMD, CT_VD_Traditional Chinese Medicine (TCM)_Fire dragon moxibustion (FDMox) (without BP) and CT_VD_BP_TCM_Acu (with BP) were best. TCM_Acu showed the best clinical efficacy. For TCM syndrome total score, CT_VD_ Du−moxibustion (DuMox) ranked lowest. For low back pain, TCM_Acu (without BP) and CT_VD_BP_Acu_Moxibustion (Mox) (with BP) were best. CT_VD_BP_Acu_Point Application Therapy (Pat) ranked lowest for VAS. CT_VD_BP_Acupoint injection (AcuInj) produced the lowest PINP, CT_VD_TCM_Acu the lowest CTX, and CT_VD_BP_TCM_Acupoint catgut embedding (ACE) the highest E2. For ALP, CT_VD_BP_Acu ranked lowest with BP, and CT_VD_Mox highest without BP. For OCN, CT_VD_Mox ranked highest without BP, and CT_VD_BP_TCM_Acu_Mox highest with BP.

**Conclusion:**

Acupuncture combined with pharmacotherapy offers differential benefits across efficacy outcomes in PMOP. However, fracture data were nearly absent (only one trial reported two hip fractures) and follow−up was short (≤12 months), limiting any inference on fracture prevention. Treatment selection should be individualized based on therapeutic priorities and patient characteristics, supporting personalized integrative management strategies.

**Systematic review registration:**

https://www.crd.york.ac.uk/PROSPERO/, identifier CRD420251233374.

## 1 Introduction

Postmenopausal osteoporosis (PMOP) is primarily driven by estrogen deficiency, which accelerates osteoclast differentiation and activity, resulting in bone resorption exceeding bone formation and leading to rapid bone loss, particularly during and after menopause (1). Epidemiological data indicate that the prevalence of osteoporosis among postmenopausal women in Brazil ranges from 15% to 33%, depending on study methodology, the use of bone mineral density (BMD) measurements, and self-reported data^2^). In the United States, osteoporosis accounts for more than 2 million fractures annually, including over 700, 000 vertebral fractures and approximately 300, 000 hip fractures, resulting in more than 500, 000 hospital admissions. These fractures substantially impair quality of life and impose a considerable burden on healthcare systems (3).

Pharmacological therapies for osteoporosis are conventionally classified into three categories (4). First, anti-resorptive agents primarily inhibit osteoclast-mediated bone resorption with limited effects on bone formation; representative drugs include bisphosphonates (BPs), denosumab, hormone replacement therapy, and raloxifene. Second, anabolic agents mainly stimulate osteoblast activity and new bone formation, although they may also influence bone resorption; examples include teriparatide, abaloparatide, and parathyroid hormone analogues. Third, phytoestrogens are non-steroidal plant-derived compounds with estrogenic or anti-estrogenic properties. Current treatment selection is largely guided by fracture risk stratification. For patients at high fracture risk, first-line therapies such as alendronate, denosumab, ibandronate, and zoledronic acid effectively reduce hip, non-vertebral, and vertebral fractures. For those at very high fracture risk or unable to tolerate oral therapy, agents including abaloparatide, denosumab, romosozumab, teriparatide, and zoledronic acid are recommended (5). However, concerns regarding long-term safety, adverse effects, and medication adherence remain substantial. Oral bisphosphonates are associated with upper gastrointestinal toxicity, including gastroesophageal reflux, esophagitis, and esophageal pain (6). Discontinuation of denosumab may result in severe rebound phenomena, such as marked increases in bone turnover markers (BTMs), rapid BMD loss, and multiple vertebral fractures, with rare but serious complications including osteonecrosis of the jaw, atypical femoral fractures, and renal impairment (7). These limitations highlight the need for safer and more sustainable therapeutic strategies for PMOP.

Acupuncture (Acu), a core modality of traditional medicine, has received increasing attention for its potential benefits in pain relief, regulation of bone metabolism, and improvement of health-related quality of life (8). Previous meta-analyses have demonstrated that acupuncture can improve BMD and estrogen levels in animal models of PMOP, with commonly used acupoints including Zusanli (ST36), Shenshu (BL23), Guanyuan (CV4), and Sanyinjiao (SP6) (9). Mechanistic studies suggest that acupuncture may modulate endocrine function by increasing serum estradiol (E2) levels and suppressing follicle-stimulating hormone (FSH), luteinizing hormone (LH), and gonadotropin-releasing hormone (GnRH), thereby restoring hormonal balance and alleviating depressive-like behaviors in perimenopausal animal models (10). In terms of bone metabolism, acupotomy—a technique integrating acupuncture with minimally invasive surgical principles—has been shown to inhibit abnormal bone resorption by upregulating the BMP2–Smad1 signaling pathway and improving the mechanical properties of subchondral bone in osteoarthritis models (11). Electroacupuncture (ElecAcu) has been reported to promote osteogenesis by activating osteoblasts and osteoclasts, modulating parathyroid hormone–calcium signaling, and stimulating bone regeneration in experimental models (12). In addition, acupuncture exerts anti-inflammatory effects; for example, specific acupuncture techniques can suppress inflammatory signaling pathways and significantly alleviate inflammatory symptoms in animal studies (13). Collectively, these findings suggest that acupuncture may alleviate PMOP by regulating endocrine function, modulating bone remodeling, and reducing inflammation.

Importantly, combining acupuncture with pharmacological therapy may yield synergistic benefits, enhancing efficacy while mitigating drug-related toxicity. The interaction between acupuncture and medications has been conceptualized in four dimensions: acupuncture-enhanced drug efficacy, drug-enhanced acupuncture efficacy, synergistic effects, and antagonistic interactions (14). Acupuncture may influence drug absorption, distribution, and metabolism, increase local or systemic drug concentrations, reduce required dosages, and attenuate adverse effects, thereby improving bioavailability. Conversely, pharmacological agents may potentiate the therapeutic effects of acupuncture, including through medicated acupuncture techniques. Moreover, traditional Chinese medicine (TCM) herbs, such as *Radix Salviae Miltiorrhizae* (Danshen), have been shown to act synergistically with acupuncture by targeting pathways such as RANKL and Wnt signaling, slowing bone loss during early menopause and promoting BMD increases in PMOP women (15).

Despite growing clinical interest, existing studies evaluating acupuncture alone or pharmacotherapy alone for PMOP have yielded inconsistent results, and robust comparative evidence regarding different acupuncture–drug combinations remains limited. Conventional pairwise meta-analyses cannot adequately address this complexity in the absence of comprehensive head-to-head trials. In contrast, network meta-analysis (NMA) enables simultaneous comparison of multiple interventions by integrating direct and indirect evidence, thereby providing higher-level evidence for optimizing treatment strategies. From a clinical perspective, practitioners require clear answers to the question of which pharmacological agents, when combined with acupuncture, offer the greatest benefit with acceptable safety profiles.

Therefore, this study aims to systematically evaluate the efficacy and safety of acupuncture combined with different medications for the treatment of PMOP using a comprehensive systematic review and Bayesian NMA. By ranking integrated treatment strategies across multiple clinically relevant outcomes, this work seeks to provide evidence-based recommendations for personalized, patient-centered management of PMOP and to inform future clinical guidelines and research directions.

## 2 Materials and methods

This systematic review and NMA were conducted in strict accordance with the Preferred Reporting Items for Systematic Reviews and Meta-Analyses for Network Meta-Analyses (PRISMA-NMA) guidelines. A completed PRISMA-NMA checklist is provided in **Supplementary Material 1**. The study protocol was prospectively registered in the International Prospective Register of Systematic Reviews (PROSPERO; registration number CRD420251233374).

### 2.1 Data sources and search strategy

We systematically searched four English-language databases and four Chinese-language databases, including PubMed, Embase, the Cochrane Library, Web of Science, China National Knowledge Infrastructure (CNKI), the China Science and Technology Journal Database (VIP), Wanfang Database, and the China Biomedical Literature Database (CBM). The search covered all records from database inception to April 11, 2026.

The search strategy comprised three core components: (1) population-related terms (e.g., “postmenopausal osteoporosis,” “post-menopausal bone loss,” “post-menopausal osteoporosis”); (2) intervention-related terms (e.g., “acupuncture,” “electroacupuncture,” “moxibustion,” “alendronate,” “calcitonin,” “vitamin D,” “traditional Chinese medicine”); and (3) study design–related terms (e.g., “randomized controlled trial,” “randomized,” “placebo”). Searches were limited to studies published in English or Chinese. The full electronic search strategies for all databases are provided in **Supplementary Material 2**.

### 2.2 Inclusion criteria

Studies were eligible for inclusion if they met the following criteria: (1) Study design: Randomized controlled trials (RCTs). (2) Study population: Women aged ≥40 years diagnosed with PMOP. Diagnostic criteria were required to meet at least one of the following conditions: (i) imaging-confirmed vertebral fractures or non-vertebral fractures within the previous five years, with BMD T-scores between −2.5 and −5.0 in women aged ≤65 years, or between −2.0 and −5.0 in women aged >65 years; (ii) women aged >65 years without a fracture history, with lumbar spine BMD (LS-BMD) or femoral neck BMD (FN-BMD) T-scores between −3.0 and −5.0. (3) Interventions: Interventions consisted of acupuncture combined with at least one pharmacological treatment, including bisphosphonates (BPs), calcitonin (CT), active vitamin D (VD), or traditional Chinese medicine (TCM), etc. Within the evidence network, most nodes represented combination therapies, defined as the concomitant use of two or more medications during the study period. Detailed definitions of all interventions are provided in **Table 1**. Drug Abbreviations (**Table 2**) and Acupuncture and Therapeutic Modalities Abbreviations (**Table 3**) are provided and displayed, respectively. (4) Outcomes: Studies were required to report at least one relevant outcome. Primary outcomes included changes in BMD, clinical efficacy, TCM syndrome scores, and Visual Analog Scale (VAS) scores. Secondary outcomes included procollagen type I N-terminal propeptide (PINP), C-terminal telopeptide (CTX), estradiol (E2), and alkaline phosphatase (ALP) and osteocalcin (OCN). Clinical efficacy was uniformly defined across studies as the proportion of patients achieving markedly effective plus effective. The three categories were: markedly effective—pain disappearing completely with a BMD change rate >2%; effective—significant pain relief with a BMD change rate between –2% and 2%; and ineffective—no significant pain relief with a BMD change rate < –2%.

**Table 1:** Definitions of intervention included in this study.

Treatment	Definitions
Bisphosphonates (BP)	First-line osteoporosis drugs that inhibit bone resorption by osteoclasts to increase bone density.
Calcitonin (CT)	A hormone-based treatment that reduces osteoclast activity and alleviates bone pain in osteoporosis.
Vitamin D (VD)	An essential nutrient that supports bone health by promoting calcium absorption and homeostasis.
Calcitriol (Cal)	The active form of vitamin D, used as a potent drug to directly regulate calcium and phosphate metabolism in bone disorders.
Estrogen (E)	Hormone replacement therapy that prevents postmenopausal bone loss by inhibiting osteoclast activity.
Placebo (Pla)	An inert control substance, physically identical to the active drug, used to benchmark specific treatment effects.
Denosumab (Deno)	A monoclonal antibody that treats osteoporosis by blocking RANKL to potently inhibit bone resorption.
Moxibustion (Mox)	A therapeutic procedure that applies heat generated by the burning of mugwort (Artemisia vulgaris) to specific acupoints, aiming to stimulate local circulation and modulate physiological functions.
Acupuncture (Acu)	A core procedure involving the insertion of fine, sterile needles into defined acupoints to elicit neuromodulatory and systemic therapeutic effects.
Electroacupuncture (ElecAcu)	A modality that enhances traditional needle stimulation by attaching electrodes to deliver a controlled, low-frequency electrical current following needle insertion.
Ginger Moxibustion (GinMox)	An indirect moxibustion technique where a slice of fresh ginger serves as an intermediary between the burning moxa and the skin, combining thermal and pharmacological stimulation.
Warm Acupuncture (WarmAcu)	A combined technique where heat from a burning moxa stick attached to a needle handle is conducted into the acupoint, integrating mechanical and thermal stimulation.
Du-moxibustion (DuMox)	A regional moxibustion protocol concentrated along the Governor Vessel (Du Mai) meridian, primarily intended to tonify yang qi.
Short pricking (ShortPri)	A superficial needling technique performed with a short needle for quick, minimal tissue penetration, commonly used for cutaneous stimulation or bloodletting.
Fire Dragon Moxibustion (FDMox)	An intensive moxibustion method where multiple lit moxa sticks are moved in a linear, "dragon-like" pattern along a meridian or muscle group to create a broad warming effect.
Thunder-Fire Moxibustion (TFMox)	A form of suspended moxibustion employing a large, densely packed, and often medicated moxa stick to deliver concentrated heat for treating pain and cold-type disorders.
Micro-needle Knife (MNKnife)	A needle-shaped instrument with a flattened, knife-like tip, used for percutaneous release of fascial constrictions or adhesions at myofascial trigger points.
Acupoint Injection (AcuInj)	A technique involving the intradermal or subcutaneous injection of a small volume of sterile solution (e.g., vitamin, herbal extract) into an acupoint for combined stimulus.
TCM Sticking (TCMStick)	The topical application of a herbal-medicated patch or plaster onto acupoints to provide sustained transdermal herbal and mechanical stimulation.
Three-edged needle (TENeedle)	A needle with a triangular body and sharp point, designed specifically for swift, shallow puncturing to induce minimal capillary bleeding.
Fire Needle (FNeedle)	A technique involving the rapid insertion of a needle that has been heated to a red-hot glow into a target point or lesion, utilizing thermal shock as the primary stimulus.
Indirect Moxibustion (IMox)	A category of moxibustion where a material (e.g., ginger, garlic, salt) is interposed between the moxa cone and the skin to attenuate and modify the heat stimulus.
Heat-sensitive Moxibustion (HSMoxibustion)	A moxibustion approach where treatment is guided by applying gentle heat to areas that spontaneously elicit radiating or penetrating thermal sensations (heat-sensitive points).
Point Application Therapy (Pat)	A non-invasive external therapy that involves securing a therapeutic agent (e.g., herbal paste, magnetic pellet) to an acupoint for prolonged contact stimulation.
Auricular Point Sticking (Aps)	A form of auriculotherapy where small, hard seeds or magnetic beads are taped to specific points on the external ear for intermittent manual stimulation.

**Table 2:** Drug abbreviations.

Category	Abbreviation
Bisphosphonate	BP
Calcitonin	CT
Vitamin D	VD
Calcitriol	Cal
Traditional Chinese Medicine	TCM
Estrogen	E
Denosumab	Deno
Celecoxib	Cel
Placebo	Pla

**Table 3:** Acupuncture and therapeutic modalities abbreviations.

Category	Abbreviation	Full name
Needling	Acu	Standard acupuncture
	ElecAcu	Electroacupuncture
	WarmAcu	Warm acupuncture
	FNeedle	Fire needle
	TENeedle	Three-edged needle
	MNKnife	Micro-needle knife
	ShortPri	Short pricking
Moxibustion	Mox	Moxibustion
	GinMox	Ginger moxibustion
	DuMox	Du-moxibustion
	FDMox	Fire dragon moxibustion
	TFMox	Thunder-fire moxibustion
	IMox	Indirect moxibustion
	HSMox	Heat-sensitive moxibustion
Other techniques	AcuInj	Acupoint injection
	Pat	Point application
	ACE	Acupoint catgut embedding
	TCMStick	TCM sticking
	Aps	Auricular point sticking
	Pla	Placebo

### 2.3 Exclusion criteria

Studies were excluded if they met any of the following criteria: (1) Reviews, meta-analyses, or other data-mining studies. (2) Case reports, books, conference abstracts, clinical guidelines, letters, editorials, brief surveys, notes, preprints, or retracted publications. (3) Duplicate publications or studies with overlapping data. (4) Studies in which the intervention or target disease did not meet the predefined inclusion criteria. (5) Studies for which the full text was unavailable. (6) Animal experiments or *in vitro* (cell-based) studies. (7) Studies published in languages other than English or Chinese. (8) Ongoing trials or registered studies without published results. (9) Studies lacking relevant outcome measures or extractable data. (10) Cohort studies.

### 2.4 Literature screening and data extraction

All retrieved records were imported into EndNote (version 20), and duplicate citations were removed using the software’s automated and manual deduplication functions. Two reviewers (Jiamei Zhuang and Dan Su) independently screened titles and abstracts to identify potentially eligible studies. Full texts of all studies deemed relevant were subsequently retrieved and assessed in detail.

Two independent reviewers evaluated the full-text articles against the predefined inclusion and exclusion criteria. Studies meeting the eligibility criteria underwent methodological quality assessment and data extraction for inclusion in the NMA. Each included study was assigned a unique identification number. Data were extracted using a standardized Microsoft Excel form and included the following items: first author, year of publication, country, study design, total sample size, participant age, intervention details, sample sizes of intervention and control groups, treatment duration, and reported outcome measures.

Any discrepancies arising during the literature screening or data extraction process were resolved through discussion; if consensus could not be reached, a third senior reviewer was consulted to make the final decision.

### 2.5 Risk of bias assessment

Two reviewers (Zhuang Jiamei and Qian Rui) independently assessed the risk of bias of the RCTs using the revised Cochrane Risk of Bias tool, version 1.0 (16). Each study was evaluated across the following domains: bias arising from the randomization process (including random sequence generation and allocation concealment), bias due to deviations from intended interventions (blinding of participants and personnel), bias in outcome measurement (blinding of outcome assessors), bias due to missing outcome data (incomplete outcome data), bias in selection of the reported result (selective outcome reporting), and other potential sources of bias. Each domain was judged as having a low risk, high risk, or some concerns of bias. Disagreements between reviewers were resolved through discussion and consensus.

In addition, the methodological quality of the included RCTs was evaluated using the modified Jadad scale (17), which assesses random sequence generation, allocation concealment, blinding, and reporting of withdrawals and dropouts. Studies scoring 1–3 points were considered to be of low methodological quality, whereas those scoring 4–7 points were regarded as high quality.

### 2.6 Statistical analysis

All statistical analyses were performed using Stata (version 17.0 with metan and metareg) and RStudio (version 4.3.1 with packages gemtc, rjags, and coda). First, network geometry plots were constructed to visually depict the structure of direct and indirect comparisons among the included interventions and to assess the overall connectivity of the evidence network.

Subsequently, NMAs were conducted within a Bayesian framework using random-effects models. For dichotomous outcomes, relative risks (RRs) with corresponding 95% credible intervals (95% CrIs) were calculated to estimate comparative treatment effects. For continuous outcomes, standardized mean differences (SMDs) with 95% CrIs were used as effect measures.

To evaluate the relative ranking of competing interventions, the surface under the cumulative ranking curve (SUCRA) was calculated for each treatment, and ranking probability plots were generated. Higher SUCRA values indicated a greater likelihood that an intervention is among the most effective options.

The assumption of network consistency was examined using two complementary approaches: (1) a loop-specific method to assess local inconsistency within closed loops of evidence, and (2) a design-by-treatment interaction model to evaluate global inconsistency across the entire network. A P value < 0.05 was considered indicative of statistically significant inconsistency between direct and indirect evidence, and such findings were interpreted with caution. Loop inconsistency resulted from Stata: CrI including 0 indicated non-significant inconsistency (**Supplementary Material 5**). In R, the Deviance Information Criterion (DIC) compared model fit and global consistency; a difference within 5 indicated good consistency, and the corresponding I² value assessed overall network heterogeneity. I² ≤ 50% indicating low heterogeneity and I² > 50% indicating substantial heterogeneity among studies (**Supplementary Material 4**). Stata generated a forest plot of global inconsistency (**Supplementary Material 6**).

League tables were generated to present pairwise effect estimates and corresponding 95% CrIs for all interventions relative to the reference treatment. Potential publication bias was assessed using comparison-adjusted funnel plots, which were visually inspected for asymmetry. Statistical significance was defined *a priori* as follows: results were considered statistically significant if the 95% CrI of an RR did not include 1 or if the 95% CrI of an SMD did not include 0.

## 3 Results

### 3.1 Study selection

A total of 1, 642 records were identified through database searches. After title and abstract screening, 454 articles were considered potentially eligible and were retrieved for full-text assessment. Of these, 342 studies were excluded for the following reasons: meta-analyses, reviews, or other data-mining studies (n = 79); case reports, conference abstracts, guidelines, and similar publications (n = 7); animal experiments (n = 13); cohort studies (n = 6); interventions or target conditions not meeting the predefined inclusion criteria (n = 230); and studies lacking relevant outcome data (n = 7).

**Figure 1:**
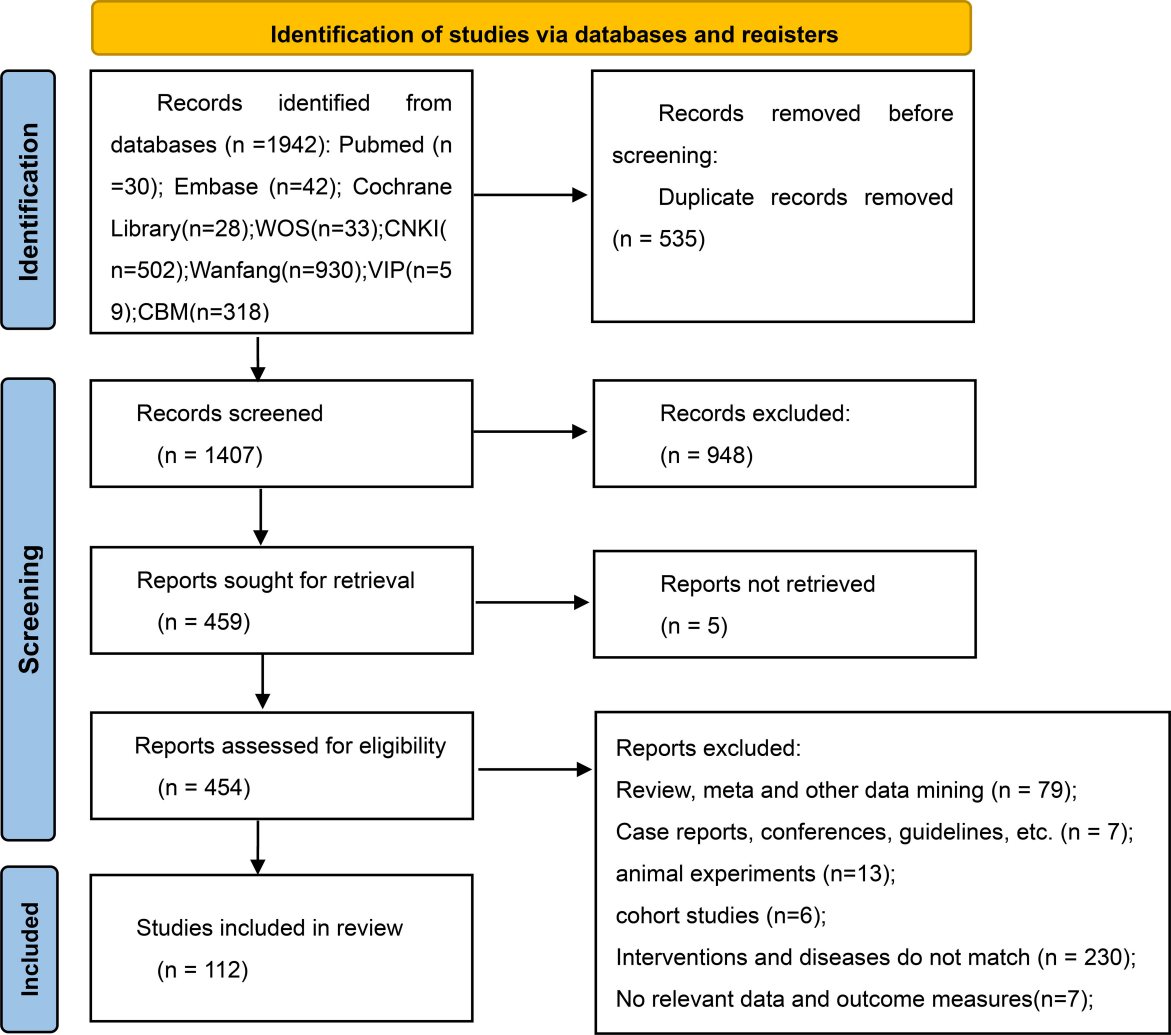
PRISMA flow diagram.

Ultimately, 112 studies (18–129) met the inclusion criteria and were included in the final analysis. The study identification, screening, and selection process is illustrated in **Figure 1**. All included studies were RCTs.

### 3.2 Basic characteristics of included studies

We included 112 RCTs in our analysis. All studies were conducted in China and published between 2000 and 2026. A total of 9, 908 participants were enrolled, all aged ≥40 years, with follow-up durations ranging from 10 days to 12 months. Diagnostic criteria for PMOP were relatively consistent across the included trials.

Intervention groups primarily received acupuncture–drug combination therapies, including but not limited to calcitonin plus vitamin D combined with acupoint embedding (CT_VD_ACE), calcitonin plus vitamin D plus traditional Chinese medicine combined with acupoint embedding (CT_VD_TCM_ACE), calcitriol combined with electroacupuncture (Cal_ElecAcu), and calcitonin plus vitamin D combined with warm acupuncture (CT_VD_WarmAcu). Control interventions included calcitonin plus vitamin D (CT_VD), calcitonin plus vitamin D combined with bisphosphonates (CT_VD_BP), traditional Chinese medicine alone (TCM), acupuncture alone (Acu), and other conventional or adjunctive therapies for PMOP.

With respect to outcome reporting, 86 studies reported BMD outcomes, 76 reported clinical efficacy, 51 reported TCM syndrome score, 53 reported VAS score, 18 reported PINP, 27 reported CTX, 26 reported E2, 20 reported ALP and 18 reported OCN. Only one trial (58) reported fracture outcomes, with hip fracture rates of 2/38 (5.3%) for CT_VD_BP_Point Application Therapy (Pat) and 1/37 (2.7%) for CT_VD_BP. No other study reported fracture events. Given the very few events and short follow−up (≤12 months), a NMA for fracture outcomes was not feasible. Detailed baseline characteristics and outcome distributions of the included studies are summarized in **Supplementary Material 3**.

### 3.3 Risk of bias assessment

For the RCTs, assessment using the modified Jadad scale showed that two studies scored 3 points and were classified as low quality, whereas the remaining 110 studies scored between 4 and 7 points and were considered high quality. According to the revised Cochrane Risk of Bias Tool 1.0, 103 studies adequately reported random sequence generation and were therefore judged to be at low risk of selection bias. 7 studies provided insufficient details regarding the randomization process and were rated as having an unclear risk of bias, while two studies were assessed as high risk due to inappropriate random sequence generation. Allocation concealment was clearly described and judged as low risk in 22 studies; 78 studies did not report allocation concealment and were therefore rated as unclear risk, and 12 studies were considered high risk because allocation concealment was compromised prior to assignment, introducing potential selection bias. With respect to blinding of participants and personnel, 23 studies lacked sufficient methodological detail and were rated as having an unclear risk of performance bias, whereas 89 studies were judged to be at high risk because participants and personnel were aware of the assigned interventions. Blinding of outcome assessment was explicitly reported in nine studies and assessed as low risk. Two studies were rated as high risk due to outcome assessors being aware of intervention allocation, and the remaining studies did not report assessor blinding and were therefore classified as having an unclear risk of detection bias. All included studies reported complete outcome data, with no evidence of selective outcome reporting or other apparent sources of bias; consequently, these domains were judged to be at low risk overall.

**Figure 2:**
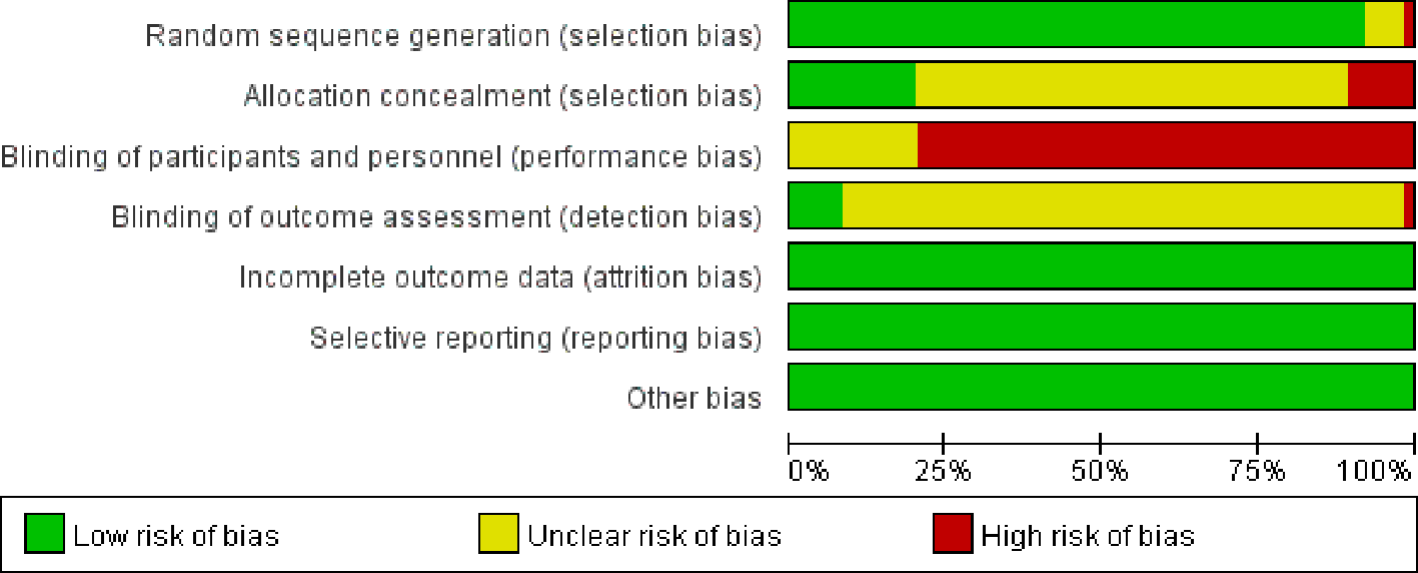
Risk of bias graph.

**Figure 3:**
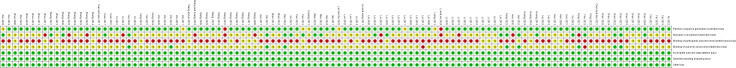
Risk of bias summary.

Detailed risk-of-bias assessments for the RCTs are presented in **Figures 2** and **3**.

### 3.4 Network meta-analysis

#### 3.4.1 BMD

##### 3.4.1.1 Lumbar spine BMD

**Figure 4:**
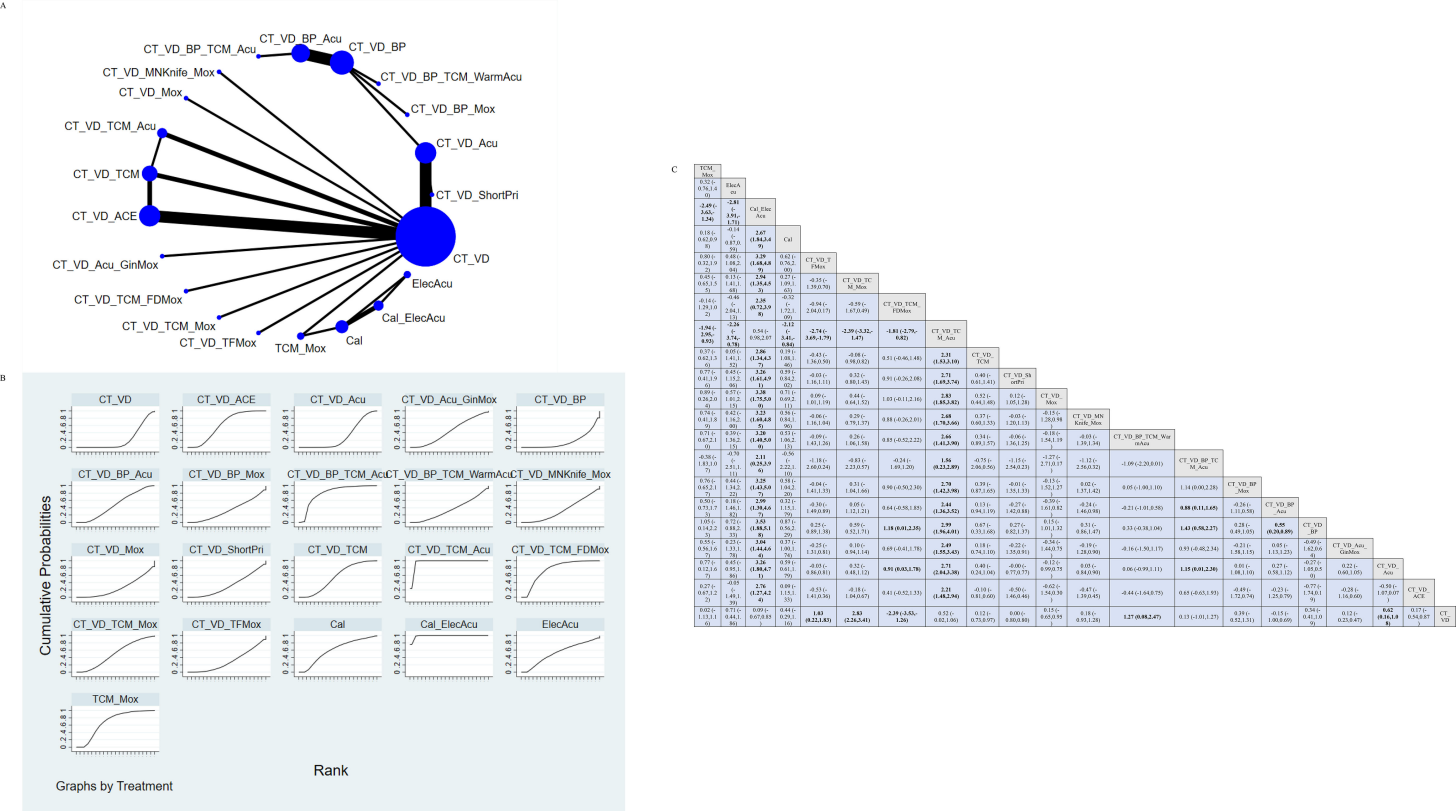
Network meta-analysis of LS-BMD. **(A)** Network plot of the network meta-analysis for LS-BMD. **(B)** Rankogram of overall intervention strategies from the network meta-analysis for LS-BMD. **(C)** League table of the network meta-analysis for LS-BMD.

A total of 56 studies evaluated the effects of acupuncture combined with different pharmacological regimens on LS-BMD, of which 33 were included in the analysis. Among these, CT_VD was the most frequently investigated intervention, followed by CT_VD_BP, CT_VD_Acu, and CT_VD_ACE. The most common direct comparisons were between CT_VD_BP_Acu and CT_VD_BP, CT_VD and CT_VD_ACE, and CT_VD and CT_VD_Acu. Overall, 23 direct treatment comparisons were formed across the included interventions. Closed loops were identified among CT_VD_TCM_Acu, CT_VD_TCM, CT_VD_ACE, and CT_VD, as well as among ElecAcu, Cal and Cal_ElecAcu, indicating the presence of indirect evidence within the network. The detailed network geometry is presented in **Figure 4A**.

Based on SUCRA values, among the 21 interventions evaluated, Cal_ElecAcu ranked highest for improving LS-BMD (SUCRA = 98.7%), followed by CT_VD_TCM_Acu (SUCRA = 96.1%) and CT_VD_BP_TCM_Acu (SUCRA = 80.1%). The overall ranking of interventions is illustrated in **Figure 4B**.

When compared with Cal_ElecAcu, multiple interventions demonstrated significantly smaller improvements in LS-BMD, including Cal (SMD = 2.67, 95% CrI: 1.84~3.49), CT_VD_Thunder−fire moxibustion (TFMox) (SMD = 3.29, 95% CrI: 1.68~4.89), CT_VD_TCM_ Moxibustion (Mox) (SMD = 2.94, 95% CrI: 1.35~4.53), CT_VD_TCM_Fire dragon moxibustion (FDMox) (SMD = 2.35, 95% CrI: 0.72~3.98), CT_VD_TCM (SMD = 2.86, 95% CrI: 1.34~4.37), CT_VD_Short pricking (ShortPri) (SMD = 3.26, 95% CrI: 1.61~4.91), CT_VD_Mox (SMD = 3.38, 95% CrI: 1.75~5.00), CT_VD_Micro−needle knife (MNKnife)_Mox (SMD = 3.23, 95% CrI: 1.60~4.85), CT_VD_BP_TCM_WarmAcu (SMD = 3.20, 95% CrI: 1.40~5.00), CT_VD_BP_TCM_Acu (SMD = 2.11, 95% CrI: 0.25~3.96), CT_VD_BP_Mox (SMD = 3.25, 95% CrI: 1.43~5.07), CT_VD_BP_Acu (SMD = 2.99, 95% CrI: 1.30~4.67), CT_VD_BP (SMD = 3.53, 95% CrI: 1.88~5.18), CT_VD_Acu_ Ginger moxibustion (GinMox) (SMD = 3.04, 95% CrI: 1.44~4.64), CT_VD_Acu (SMD = 3.26, 95% CrI: 1.80~4.71), and CT_VD_ACE (SMD = 2.76, 95% CrI: 1.27~4.24). Similarly, when compared with CT_VD_TCM_Acu, several interventions were associated with significantly smaller increases in LS-BMD, including CT_VD_TCM (SMD = 2.31, 95% CrI: 1.52~3.10), CT_VD_ShortPri (SMD = 2.71, 95% CrI: 1.69~3.74), CT_VD_Mox (SMD = 2.83, 95% CrI: 1.85~3.82), CT_VD_Micro−needle knife (MNKnife)_Mox (SMD = 2.68, 95% CrI: 1.70~3.66), CT_VD_BP_TCM_WarmAcu (SMD = 2.66, 95% CrI: 1.41~3.90), CT_VD_BP_TCM_Acu (SMD = 1.56, 95% CrI: 0.23~2.89), CT_VD_BP_Mox (SMD = 2.70, 95% CrI: 1.42~3.98), CT_VD_BP_Acu (SMD = 2.44, 95% CrI: 1.36~3.52), CT_VD_BP (SMD = 2.99, 95% CrI: 1.96~4.01), CT_VD_Acu_GinMox (SMD = 2.49, 95% CrI: 1.55~3.43), CT_VD_Acu (SMD = 2.71, 95% CrI: 2.04~3.38), and CT_VD_ACE (SMD = 2.21, 95% CrI: 1.48~2.94). When compared with CT_VD_BP, both CT_VD_TCM_FDMox (SMD = 1.18, 95% CrI: 0.01~2.35) and CT_VD_BP_Acu (SMD = 0.55, 95% CrI: 0.20~0.89) led to superior gains in LS−BMD. Relative to CT_VD_Acu, CT_VD_TCM_FDMox (SMD = 0.91, 95% CrI: 0.03~1.78), CT_VD_BP_TCM_Acu (SMD = 1.15, 95% CrI: 0.01~2.30) demonstrated superior efficacy. Additionally, CT_VD_ Thunder−fire moxibustion (TFMox) (SMD = 1.03, 95% CrI: 0.22~1.83), CT_VD_TCM_Mox (SMD = 2.83, 95% CrI: 2.26~3.41), CT_VD_TCM_FDMox (SMD = -2.39, 95% CrI: -3.52~-1.26), CT_VD_BP_TCM_WarmAcu (SMD = 1.27, 95% CrI: 0.08~2.47)and CT_VD_Acu (SMD = 0.62, 95% CrI: 0.16~1.08) outperformed CT_VD in terms of LS−BMD improvement. Relative to CT_VD_TCM_Acu, several interventions yielded significantly less improvement in LS−BMD, namely TCM_Mox (SMD = -1.94, 95% CrI: -2.95~-0.93), ElecAcu (SMD = -2.26, 95% CrI: -3.74~-0.78), Cal (SMD = -2.12, 95% CrI: -3.41~-0.84), CT_VD_TFMox (SMD = -2.74, 95% CrI: -3.69~-1.79), CT_VD_TCM_Mox (SMD = -2.39, 95% CrI: -3.32~-1.47) and CT_VD_TCM_FDMox (SMD = -1.81, 95% CrI: -2.79~-0.82). When Cal_ElecAcu served as the reference, a significantly smaller LS-BMD gain was observed for multiple interventions, including TCM_Mox (SMD = -2.49, 95% CrI: -3.63~-1.34) and ElecAcu (SMD = -2.81, 95% CrI: -3.91~-1.71). A significantly smaller LS-BMD response was observed for multiple interventions relative to CT_VD_BP_TCM_Acu, including CT_VD_BP_Acu (SMD = 0.88, 95% CrI: 0.11–1.65) and CT_VD_BP (SMD = 1.43, 95% CrI: 0.58–2.27). Detailed effect estimates are presented in the league table (**Figure 4C**).

The global inconsistency test indicated statistically significant inconsistency (P < 0.001), whereas overall heterogeneity was low (I² = 3%). Three closed loops were identified within the evidence network. The 95% CrIs for the loops CT_VD–CT_VD_ACE–CT_VD_TCM and Cal–Cal_ElecAcu–ElecAcu crossed zero, suggesting no significant loop inconsistency. The forest plot for global inconsistency is provided in **Supplementary Material 6** (**Supplementary Figure 1.1**), whereas the forest plot for LS-BMD is displayed in **Supplementary Material 7** (**Supplementary Figure 2.1**).

##### 3.4.1.2 Femoral neck BMD

We divided the interventions into two groups: one containing BP and the other without BP. A total of 28 studies evaluated the effects of acupuncture combined with different pharmacological regimens on FN-BMD. After screening, 17 studies were included in the analysis: 9 studies did not contain BP, and 8 studies contained BP.

**Figure 5:**
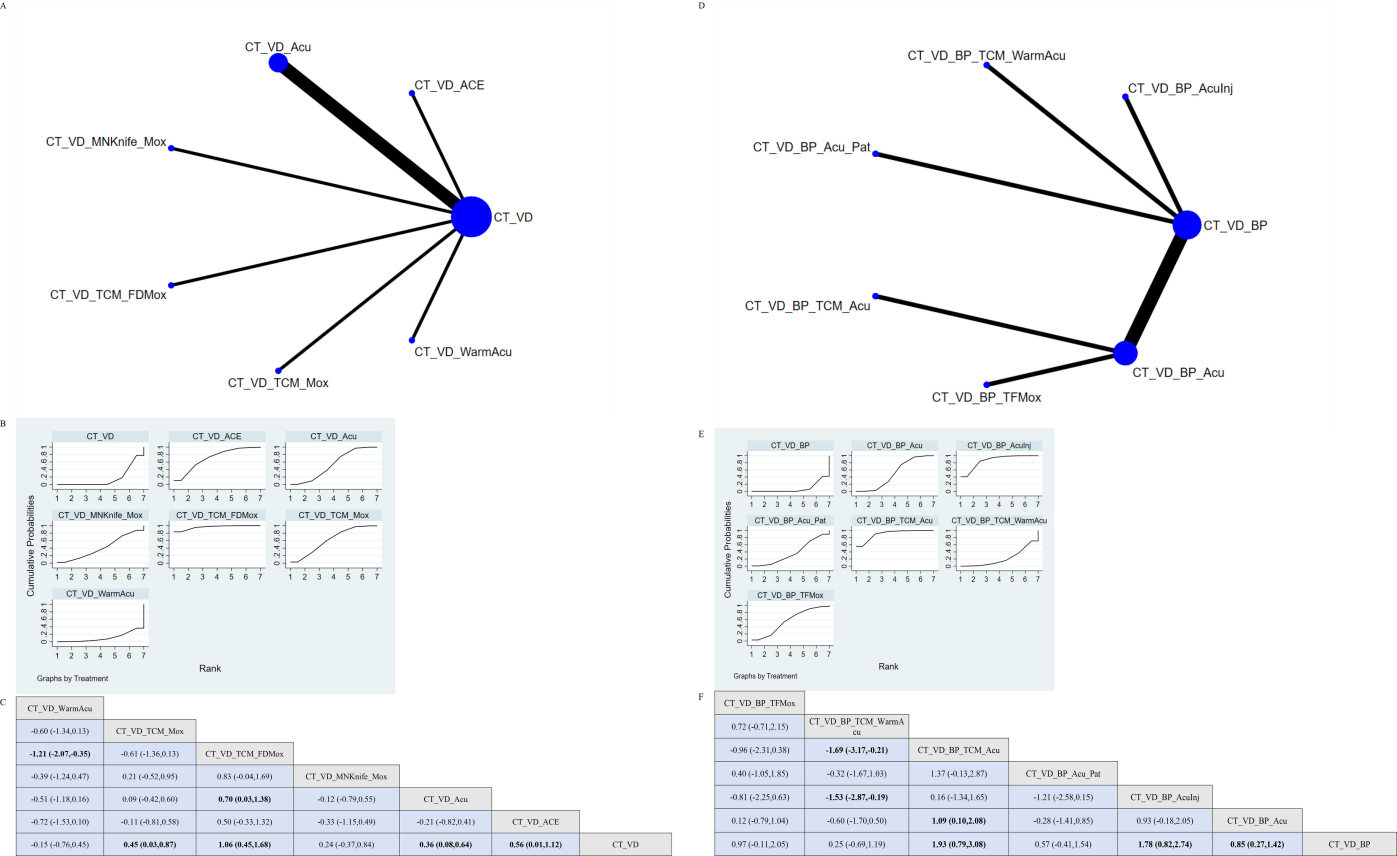
Network meta-analysis of FN-BMD. **(A)** Network plot of the network meta-analysis for the BP‑free FN-BMD. **(B)** Rankogram of overall intervention strategies from the network meta-analysis for the BP‑free FN-BMD. **(C)** League table of the network meta-analysis for the BP‑free FN-BMD. **(D)** Network plot of the network meta-analysis for the BP‑containing FN-BMD. **(E)** Rankogram of overall intervention strategies from the network meta-analysis for the BP‑ containing FN-BMD. **(F)** League table of the network meta-analysis for the BP‑ containing FN-BMD.

For the BP−free interventions, CT_VD was the most frequently studied intervention, with CT_VD_Acu ranking second. The most common direct comparison was between CT_VD and CT_VD_Acu. A total of six direct pairwise comparisons were generated among the included interventions. No closed loops were found in the evidence network, implying the absence of indirect comparisons. The detailed structure of the network is illustrated in **Figure 5A**. According to the SUCRA values, CT_VD_TCM_FDMox was the highest−ranked intervention for improving FN−BMD among the 7 evaluated (SUCRA = 96.1%), followed by CT_VD_ACE (70.4%) and CT_VD_TCM_Mox (61.9%). The overall ranking is displayed in **Figure 5B**.

Compared with CT_VD_TCM_FDMox, the following interventions were associated with markedly smaller improvements in FN-BMD: CT_VD_WarmAcu (SMD = -1.21, 95% CrI: -2.07~-0.35), CT_VD (SMD = 1.06, 95% CrI: 0.45~1.68), CT_VD_Acu (SMD = 0.70, 95% CrI: 0.03~1.38). When set against CT_VD, CT_VD_TCM_Mox (SMD = 0.45, 95% CrI: 0.03~0.87), CT_VD_Acu (SMD = 0.36, 95% CrI: 0.08~0.64) and CT_VD_ACE (SMD = 0.56, 95% CrI: 0.01~1.12) proved more effective at increasing FN- BMD. Detailed SMDs and corresponding 95% CrIs are provided in **Figure 5C**. The global inconsistency test showed no significant inconsistency (P = 0.623), and overall heterogeneity was low (I² = 0%). As no closed loops were formed within the network, loop-specific inconsistency was not assessed. The forest plot for global inconsistency is presented in **Supplementary Material 6** (**Supplementary Figure 1.2**), whereas the forest plot for the BP-free LS BMD is shown in **Supplementary Material 7** (**Supplementary Figure 2.2**).

For the BP−containing interventions, the most frequently investigated intervention was CT_VD_BP, followed by CT_VD_BP_Acu. Direct comparisons most often involved CT_VD_BP and CT_VD_BP_Acu. Across the included interventions, 6 direct pairwise comparisons were identified. The evidence network contained no closed loops, indicating that no indirect comparisons were present. **Figure 5D** shows the detailed network geometry. With SUCRA values of 90.4%, 86.3% and 56%, respectively, CT_VD_BP_TCM_Acu ranked first for BP−containing FN-BMD improvement among the 7 interventions, followed by CT_VD_BP_Acupoint injection (AcuInj) and CT_VD_BP_TFMox. The complete ranking is presented in **Figure 5E**.

A higher FN-BMD increment was observed for CT_VD_BP_AcuInj (SMD = 1.78, 95% CrI: 0.82~2.74), CT_VD_BP_Acu (SMD = 0.85, 95% CrI: 0.27~1.42) and CT_VD_BP_TCM_Acu (SMD = 1.93, 95% CrI: 0.79~3.08) than for CT_VD_BP. CT_VD_BP_TCM_WarmAcu served as the comparator; CT_VD_BP_AcuInj (SMD = -1.53, 95% CrI: -2.87~-0.19) and CT_VD_BP_TCM_Acu (SMD = -1.69, 95% CrI: -3.17~-0.21) achieved a better FN-BMD response. CT_VD_BP_TCM_Acu (SMD = 1.09, 95% CrI: 0.10~2.08) conferred a greater FN-BMD benefit than CT_VD_BP_Acu. The league table of is presented in **Figure 5F**. No significant inconsistency was detected by the global inconsistency test (P = 0.789), and overall heterogeneity was low (I² = 5%). Because no closed loops were formed within the network, loop−specific inconsistency was not assessed. **Supplementary Material 6** (**Supplementary Figure 1.3**) provides the forest plot for global inconsistency, while **Supplementary Material 7** (**Supplementary Figure 2.3**) displays the forest plot for the BP−containing FN-BMD.

#### 3.4.2 Clinical efficacy

76 studies assessed the effects of acupuncture combined with different pharmacological regimens on clinical efficacy. Among them, 42 were included in the analysis.

**Figure 6:**
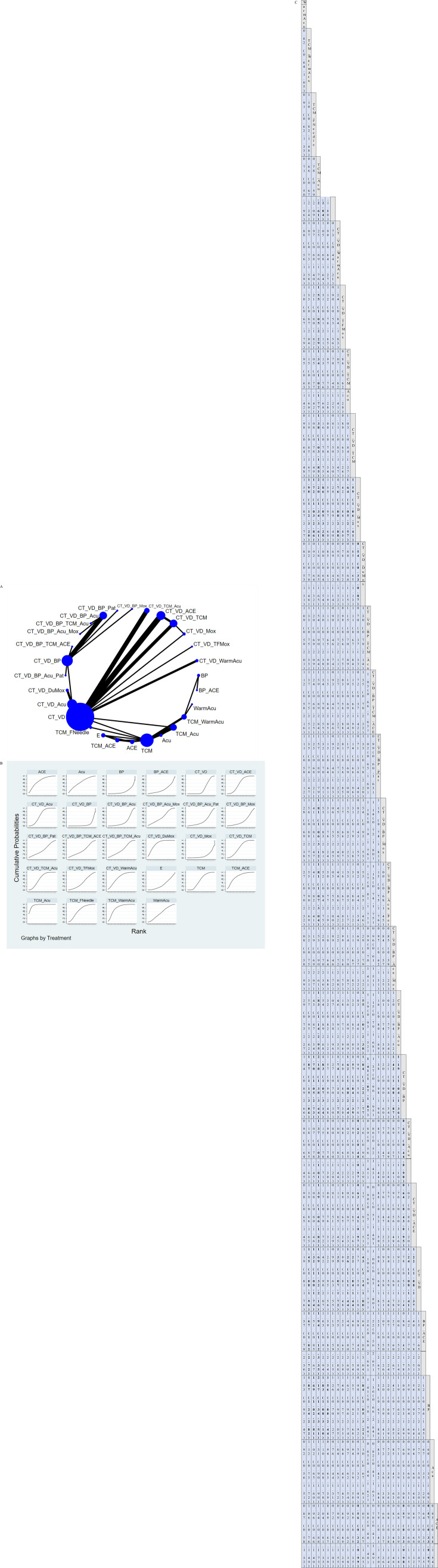
Network meta-analysis of clinical efficacy. **(A)** Network plot of the network meta-analysis for clinical efficacy. **(B)** Rankogram of overall intervention strategies from the network meta-analysis for clinical efficacy. **(C)** League table of the network meta-analysis for clinical efficacy.

CT_VD was the intervention investigated most often, TCM was the next most common, and CT_VD_BP was the third most common. The direct comparison between CT_VD_BP_Acu and CT_VD_BP, as well as between CT_VD and CT_VD_ACE was the most frequent. In total, 34 direct pairwise comparisons were generated from the included interventions. Closed loops were detected among CT_VD, CT_VD_ACE, CT_VD_TCM, CT_VD_Mox, as well as among CT_VD, TCM, Acu, TCM_Acu, suggesting the existence of indirect evidence. See **Figure 6A** for the detailed network geometry.

SUCRA values placed TCM_Acu at the top for clinical efficacy (SUCRA = 95.7%), ahead of TCM_WarmAcu (SUCRA = 82%) and CT_VD_Du−moxibustion (DuMox) (SUCRA = 80.9%) among the 28 interventions assessed. **Figure 6B** illustrates the overall ranking.

Relative to CT_VD_Mox, greater clinical efficacy improvements were observed for TCM_WarmAcu (RR = 1.9, 95% CrI: 1.12~3.2), TCM_ Fire needle (FNeedle) (RR = 1.72, 95% CrI: 1.03~2.86), TCM_Acu (RR = 2.2, 95% CrI: 1.34~3.61), TCM_ACE (RR = 1.86, 95% CrI: 1.05~3.28), CT_VD_WarmAcu (RR = 1.76, 95% CrI: 1.08~2.88), CT_VD_TCM_Acu (RR = 1.64, 95% CrI: 1.05~2.57), CT_VD_TCM (RR = 1.59, 95% CrI: 1.06~2.41), CT_VD_DuMox (RR = 0.54, 95% CrI: 0.33~0.87), CT_VD_Acu (RR = 0.62, 95% CrI: 0.4~0.97), CT_VD_ACE (RR = 0.63, 95% CrI: 0.41~0.97), ACE (RR = 0.56, 95% CrI: 0.32~0.99). Relative to CT_VD_BP, a greater improvement in clinical efficacy was noted for TCM_WarmAcu (RR = 1.87, 95% CrI: 1.15~3.06), TCM_FNeedle (RR = 1.7, 95% CrI: 1.05~2.73), TCM_Acu (RR = 2.18, 95% CrI: 1.38~3.44), TCM_ACE (RR = 1.83, 95% CrI: 1.07~3.14), CT_VD_WarmAcu (RR = 1.74, 95% CrI: 1.11~2.75), CT_VD_TCM_Acu (RR = 1.62, 95% CrI: 1.08~2.44), CT_VD_TCM (RR = 1.57, 95% CrI: 1.04~2.39), CT_VD_DuMox (RR = 1.84, 95% CrI: 1.22~2.77), CT_VD_BP_TCM_Acu (RR = 1.41, 95% CrI: 1.07~1.87), CT_VD_BP_Acu_Mox (RR = 1.42, 95% CrI: 1.08~1.87), CT_VD_BP_Acu (RR = 1.19, 95% CrI: 1.04~1.36), CT_VD_Acu (RR = 0.63, 95% CrI: 0.44~0.9), CT_VD_ACE (RR = 0.64, 95% CrI: 0.43~0.95), ACE (RR = 0.57, 95% CrI: 0.33~0.98). Using CT_VD as the reference, TCM_WarmAcu (RR = 1.45, 95% CrI: 1.08~1.96), TCM_FNeedle (RR = 1.32, 95% CrI: 1~1.74), TCM_Acu (RR = 1.69, 95% CrI: 1.32~2.16), CT_VD_WarmAcu (RR = 1.35, 95% CrI: 1.07~1.72), CT_VD_TCM_Acu (RR = 1.26, 95% CrI: 1.11~1.44), CT_VD_TCM (RR = 1.22, 95% CrI: 1.04~1.44), CT_VD_DuMox (RR = 1.43, 95% CrI: 1.14~1.8), CT_VD_Acu (RR = 1.24, 95% CrI: 1.08~1.41), CT_VD_ACE (RR = 1.22, 95% CrI: 1.08~1.37) showed greater improvements in clinical efficacy. Relative to BP_ACE, a greater improvement in clinical efficacy was noted for TCM_WarmAcu (RR = 1.67, 95% CrI: 1.02~2.72), TCM_Acu (RR = 1.94, 95% CrI: 1.12~3.35). Relative to BP, TCM_WarmAcu (RR = 1.87, 95% CrI: 1.24~2.82), TCM_FNeedle (RR = 1.69, 95% CrI: 1.02~2.81), TCM_Acu (RR = 2.17, 95% CrI: 1.34~3.51), TCM_ACE (RR = 1.83, 95% CrI: 1.08~3.1), TCM (RR = 1.56, 95% CrI: 1~2.44), CT_VD_DuMox (RR = 1.84, 95% CrI: 1.05~3.21), ACE (RR = 0.57, 95% CrI: 0.33~0.97) led to greater improvements in clinical efficacy. A significantly lesser degree of clinical efficacy was seen for several interventions versus TCM_Acu, specifically TCM (RR = 1.39, 95% CrI: 1.17~1.66), E (RR = 1.72, 95% CrI: 1.13~2.61), CT_VD_TFMox (RR = 1.55, 95% CrI: 1.05~2.29), CT_VD_TCM_Acu (RR = 1.34, 95% CrI: 1.02~1.77), CT_VD_TCM (RR = 1.38, 95% CrI: 1.03~1.85), CT_VD_BP_Mox (RR = 1.81, 95% CrI: 1.06~3.12), CT_VD_BP_Acu_Pat (RR = 1.81, 95% CrI: 1.07~3.04), CT_VD_BP_Acu (RR = 1.83, 95% CrI: 1.14~2.95), CT_VD_Acu (RR = 1.37, 95% CrI: 1.03~1.81), CT_VD_ACE (RR = 1.39, 95% CrI: 1.06~1.82). TCM_ACE (RR = 1.44, 95% CrI: 1.02~2.04) produced greater improvements in clinical efficacy relative to E. Compared to CT_VD_BP_Acu, CT_VD_DuMox (RR = 1.55, 95% CrI: 1.01~2.38) yielded greater clinical efficacy improvements. Detailed effect estimates with corresponding 95% CrIs are presented in **Figure 6C**.

The global inconsistency test demonstrated no significant inconsistency (P = 0.517), and heterogeneity was absent (I² = 4%). For the four identified closed loops (CT_VD–CT_VD_Mox–CT_VD_TCM; CT_VD–CT_VD_ACE–CT_VD_TCM; Acu–TCM–TCM_Acu; and CT_VD–TCM–TCM_Acu), all 95% CrIs crossed zero, indicating no significant loop-specific inconsistency. The forest plot for global inconsistency can be found in **Supplementary Material 6** (**Supplementary Figure 1.4**), whereas the forest plot for clinical efficacy is located in **Supplementary Material 7** (**Supplementary Figure 2.4**).

#### 3.4.3 TCM syndrome score

We identified 51 studies examining the effects of acupuncture combined with different pharmacological regimens on TCM syndrome score. Among them, 23 met our criteria for the total score analysis, and 16 met the criteria for the low back pain score analysis.

##### 3.4.3.1 The total score of TCM syndrome

**Figure 7:**
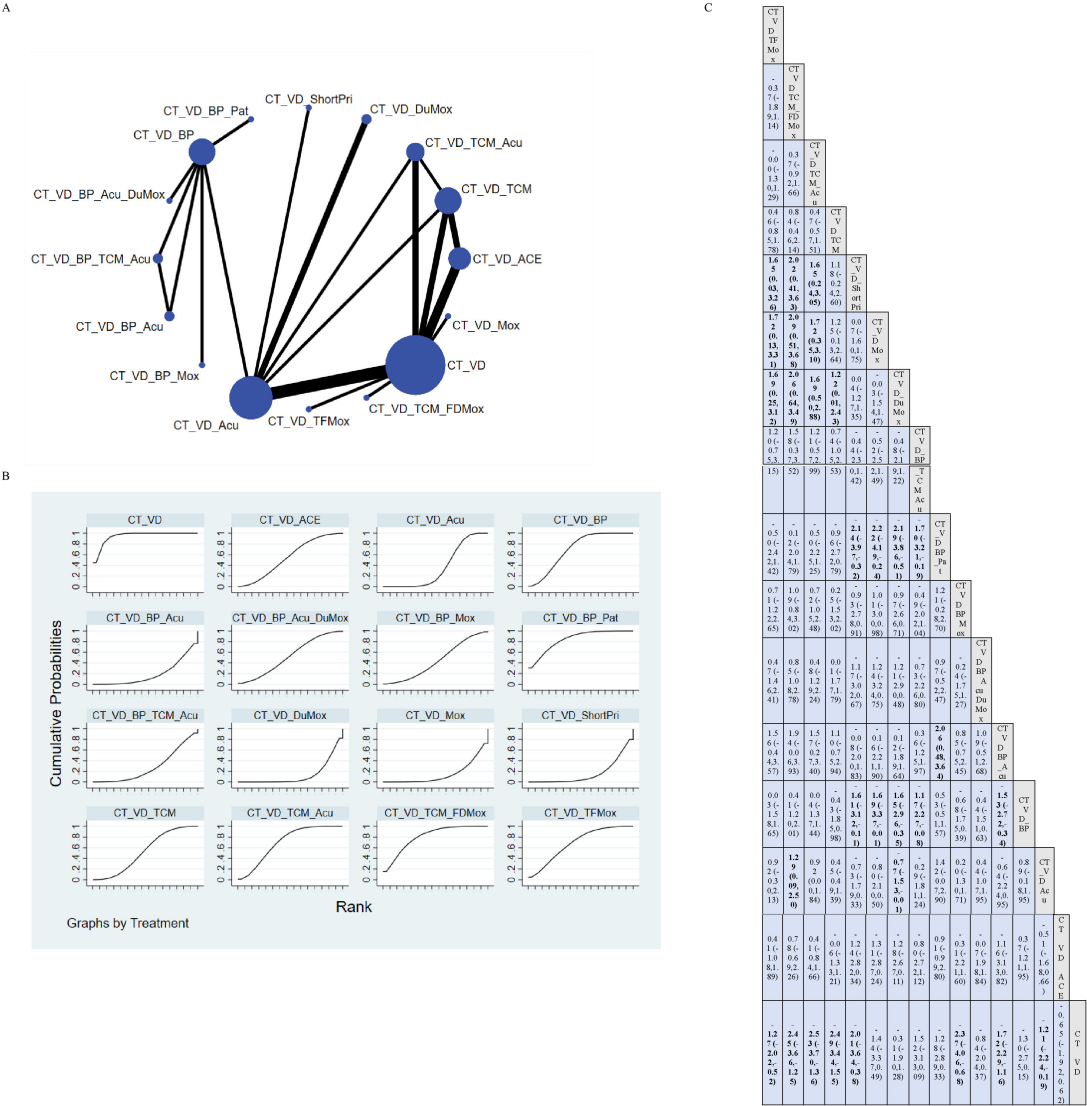
Network meta-analysis of TCM syndrome score. **(A)** Network plot of the network meta-analysis for the total TCM syndrome score. **(B)** Rankogram of overall intervention strategies from the network meta-analysis for the total TCM syndrome score. **(C)** League table of the network meta-analysis for the total TCM syndrome score.

Among all interventions for total TCM syndrome score, CT_VD had the highest frequency of investigation, followed by CT_VD_Acu, CT_VD_TCM and CT_VD_BP. The most common direct comparison was that between CT_VD and CT_VD_Acu, followed by that between CT_VD and CT_VD_ACE. Overall, 20 direct pairwise comparisons were produced across the included interventions. Indirect evidence was present, as closed loops were found both among CT_VD, CT_VD_Acu, CT_VD_TCM, CT_VD_ACE, and CT_VD_TCM_Acu. In **Figure 7A** lies the detailed network structure.

Based on SUCRA values, CT_VD_DuMox proved to be the most effective intervention in reducing total TCM syndrome score among the 16 evaluated (SUCRA = 13.5%), followed by CT_VD_Mox (SUCRA = 15%) and CT_VD_ShortPri (SUCRA = 16.2%). Refer to **Figure 7B** for the complete ranking.

The total TCM syndrome score was significantly higher with CT_VD_TFMox (SMD = 1.65, 95% CrI: 0.03~3.26), CT_VD_TCM_FDMox (SMD = 2.02, 95% CrI: 0.41~3.63), CT_VD_TCM_Acu (SMD = 1.65, 95% CrI: 0.24~3.05), CT_VD_BP_Pat (SMD = -2.14, 95% CrI: -3.97~-0.31), CT_VD_BP (SMD = -1.61, 95% CrI: -3.12~-0.11) and CT_VD (SMD = -2.01, 95% CrI: -3.64~-0.38) than with CT_VD_ShortPri. When set against CT_VD_Mox, CT_VD_TFMox (SMD = 1.72, 95% CrI: 0.13~3.31), CT_VD_TCM_FDMox (SMD = 2.09, 95% CrI: 0.51~3.68), CT_VD_TCM_Acu (SMD = 1.72, 95% CrI: 0.35~3.10), CT_VD_BP_Pat (SMD = -2.22, 95% CrI: -4.19~-0.24), CT_VD_BP (SMD = -1.69, 95% CrI: -3.37~-0.01) proved less effective at decreasing the total TCM syndrome score. Taking CT_VD_DuMox as the comparator, all other listed interventions showed significantly smaller total TCM syndrome score improvements: CT_VD_TFMox (SMD = 1.69, 95% CrI: 0.25~3.12), CT_VD_TCM_FDMox (SMD = 2.06, 95% CrI: 0.64~3.49), CT_VD_TCM_Acu (SMD = 1.69, 95% CrI: 0.50~2.88), CT_VD_TCM (SMD = 1.22, 95% CrI: 0.01~2.43), CT_VD_BP_Pat (SMD = -2.19, 95% CrI: -3.86~-0.51), CT_VD_BP (SMD = -1.65, 95% CrI: -2.96~-0.35) and CT_VD_Acu (SMD = -0.77, 95% CrI: -1.53~-0.01). In contrast to CT_VD, several interventions produced significantly less pronounced total TCM syndrome score, namely CT_VD_TFMox (SMD = -1.27, 95% CrI: -2.02~-0.52), CT_VD_TCM_FDMox (SMD = -2.45, 95% CrI: -3.66~-1.25), CT_VD_TCM_Acu (SMD = -2.53, 95% CrI: -3.70~-1.36), CT_VD_TCM (SMD = -2.49, 95% CrI: -3.44~-1.55), CT_VD_BP_Mox (SMD = -2.37, 95% CrI: -4.06~-0.68), CT_VD_BP_Acu (SMD = -1.72, 95% CrI: -2.29~-1.16) and CT_VD_Acu (SMD =-1.21, 95% CrI: -2.24~-0.19). A significantly smaller total TCM syndrome score response was observed for multiple interventions relative to CT_VD_BP_TCM_Acu, including CT_VD_BP_Pat (SMD =-1.70, 95% CrI: -3.21~-0.19) and CT_VD_BP (SMD =-1.17, 95% CrI: -2.27~-0.08). When benchmarked against CT_VD_BP_Acu, the following interventions each yielded significantly smaller improvements in total TCM syndrome score: CT_VD_BP_Pat (SMD = 2.06, 95% CrI: 0.48~3.64) and CT_VD_BP (SMD =-1.53, 95% CrI: -2.72~-0.34). Compared with CT_VD_TCM_FDMox, CT_VD_Acu (SMD = 1.29, 95% CrI: 0.09~2.50) was associated with significantly lower TCM syndrome score. All effect estimates are summarized in **Figure 7C**.

The global inconsistency test showed significant inconsistency (P = 0.001), and heterogeneity was low (I² = 2%). Five closed loops were identified within the network. Among these, two loops (CT_VD–CT_VD_Acu–CT_VD_TCM_Acu and CT_VD–CT_VD_Acu–CT_VD_TCM) had 95% CrIs that included zero, indicating no significant loop inconsistency. The forest plot for global inconsistency for the total TCM syndrome score and the forest plot are given in **Supplementary Material 6** (**Supplementary Figure 1.6**) and depicted in **Supplementary Material 7** (**Supplementary Figure 2.6**), respectively.

##### 3.4.3.2 The low back pain score of TCM syndrome

For the outcome of low back pain, interventions in 9 studies included BP, while those in 7 studies did not include BP. We analyzed them separately.

**Figure 8:**
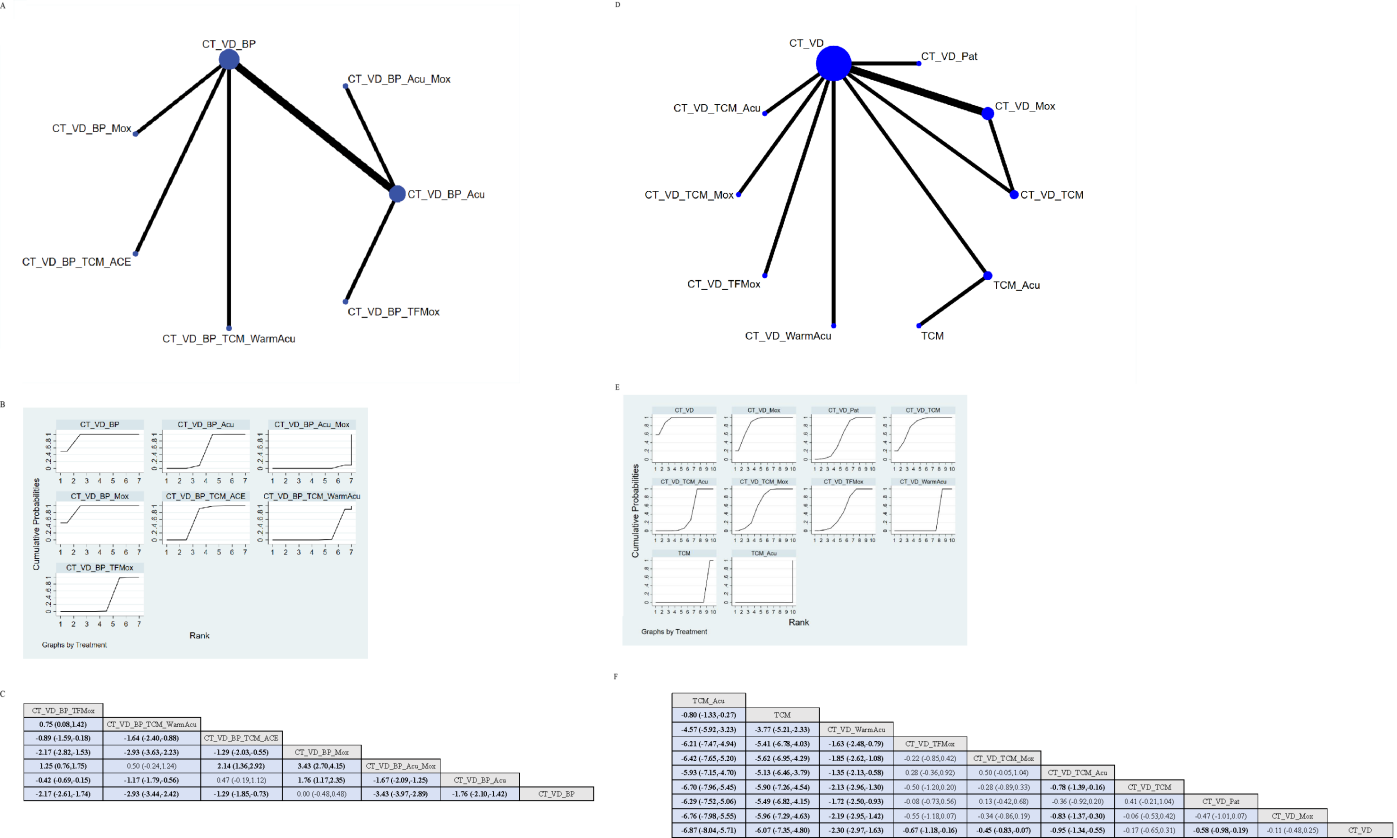
Network meta-analysis of low back pain score of TCM syndrome score. **(A)** Network plot of the network meta-analysis for low back pain score of TCM syndrome score (include BP). **(B)** Rankogram of overall intervention strategies from the network meta-analysis for low back pain score of TCM syndrome score (include BP). **(C)** League table of the network meta-analysis for low back pain score of TCM syndrome score (include BP). **(D)** Network plot of the network meta-analysis for low back pain score of TCM syndrome score (exclude BP). **(E)** Rankogram of overall intervention strategies from the network meta-analysis for low back pain score of TCM syndrome score (exclude BP). **(F)** League table of the network meta-analysis for low back pain score of TCM syndrome score (exclude BP).

For the BP-containing interventions, the most frequently investigated intervention was CT_VD_BP, followed by CT_VD_BP_Acu. Direct comparisons most often involved CT_VD_BP and CT_VD_BP_Acu. Across the included interventions, six direct pairwise comparisons were identified. The evidence network contained no closed loops, indicating that no indirect comparisons were present. **Figure 8A** shows the detailed network geometry. Of the seven interventions evaluated, CT_VD_BP_Acu_Mox was ranked first for improving low back pain score (SUCRA = 13.5%), followed by CT_VD_BP_TCM_WarmAcu (15.2%) and CT_VD_BP_TFMox (33.3%), as per SUCRA values. **Figure 8B** displays the overall ranking.

Compared with CT_VD_BP_TFMox, lower low back pain scores were seen with CT_VD_BP_TCM_WarmAcu (SMD = 0.75, 95% CrI: 0.08~1.42), CT_VD_BP_Acu_Mox (SMD = 1.25, 95% CrI: 0.76~1.75); in contrast, CT_VD_BP_TCM_ACE (SMD = -0.89, 95% CrI: -1.59~-0.18), CT_VD_BP_Mox (SMD = -2.17, 95% CrI: -2.82~-1.53), CT_VD_BP_Acu (SMD = -0.42, 95% CrI: -0.69~-0.15) and CT_VD_BP (SMD = -2.17, 95% CrI: -2.61~-1.74) were associated with a higher scores. Compared with CT_VD_BP_TCM_WarmAcu, the following interventions were associated with markedly smaller improvements in low back pain scores: CT_VD_BP_TCM_ACE (SMD = -1.64, 95% CrI: -2.40~-0.88), CT_VD_BP_Mox (SMD = -2.93, 95% CrI: -3.63~-2.23), CT_VD_BP_Acu (SMD =-1.17, 95% CrI: -1.79~-0.56) and CT_VD_BP (SMD = -2.93, 95% CrI: -3.44~-2.42). CT_VD_BP_Mox (SMD = -1.29, 95% CrI: -2.03~-0.55) and CT_VD_BP (SMD = -1.29, 95% CrI: -1.85~-0.73) were associated with a smaller decrease in low back pain scores than CT_VD_BP_TCM_ACE, while CT_VD_BP_Acu_Mox (SMD = 2.14, 95% CrI: 1.36~2.92) produced an even smaller decrease. CT_VD_BP_Acu_Mox (SMD = 3.43, 95% CrI: 2.70~4.15) and CT_VD_BP_Acu (SMD = 1.76, 95% CrI: 1.17~2.35) each led to larger low back pain scores reduction compared with CT_VD_BP_Mox. A significantly lesser degree of low back pain scores reduction was seen for several interventions versus CT_VD_BP_Acu_Mox, specifically CT_VD_BP_Acu (SMD = -1.67, 95% CrI: -2.09~-1.25), CT_VD_BP (SMD = -3.43, 95% CrI: -3.97~-2.89). In terms of low back pain scores decrease, CT_VD_BP_Acu (SMD = -1.76, 95% CrI: -2.10~-1.42) both ranked above CT_VD_BP. All detailed effect estimates are displayed in **Figure 8C**. Significant global inconsistency was detected (P = 0.002), and heterogeneity was low (I² = 33%), while no closed loop was formed in the network; consequently, loop−specific inconsistency was not evaluated. **Supplementary Material 6** (**Supplementary Figure 1.7**) gives the forest plot for global inconsistency, whereas the forest plot for low back pain score can be accessed in **Supplementary Material 7** (**Supplementary Figure 2.7**).

For the BP-free interventions, CT_VD ranked first in terms of investigation frequency. The direct pairwise comparison most frequently observed was between CT_VD and CT_VD_Mox. A total of 10 such direct comparisons were generated among the included interventions. A closed loop was observed, including among CT_VD, CT_VD_Mox, and CT_VD_TCM, pointing to the presence of indirect evidence. **Figure 8D** provides the detailed network geometry. TCM_Acu ranked highest for reducing low back pain among the 10 interventions (SUCRA = 0%), followed by CM (SUCRA = 11.1%) and CT_VD_WarmAcu (SUCRA = 22.2%), as determined by SUCRA values. The overall ranking is depicted in **Figure 8E**.

TCM (SMD = −0.80, 95% CrI: −1.33~−0.27), CT_VD_WarmAcu (SMD = −4.57, 95% CrI: −5.92~−3.23), CT_VD_TFMox (SMD = −6.21, 95% CrI: −7.47~−4.94), CT_VD_TCM_Mox (SMD = −6.42, 95% CrI: −7.65~−5.20), CT_VD_TCM_Acu (SMD = −5.93, 95% CrI: −7.15~−4.70), CT_VD_TCM (SMD = −6.70, 95% CrI: −7.96~−5.45), CT_VD_Pat (SMD = −6.29, 95% CrI: −7.52~−5.06), CT_VD_Mox (SMD = −6.76, 95% CrI: −7.98~−5.55), and CT_VD (SMD = −6.87, 95% CrI: −8.04~−5.71) conferred higher low back pain score than TCM_Acu. CT_VD_WarmAcu (SMD = −3.77, 95% CrI: −5.21~−2.33), CT_VD_TFMox (SMD = −5.41, 95% CrI: −6.78~−4.03), CT_VD_TCM_Mox (SMD = −5.62, 95% CrI: −6.95~−4.29), CT_VD_TCM_Acu (SMD = −5.13, 95% CrI: −6.46~−3.79), CT_VD_TCM (SMD = −5.90, 95% CrI: −7.26~−4.54), CT_VD_Pat (SMD = −5.49, 95% CrI: −6.82~−4.15), CT_VD_Mox (SMD = −5.96, 95% CrI: −7.29~−4.63), and CT_VD (SMD = −6.07, 95% CrI: −7.35~−4.80) each led to higher low back pain score compared with TCM. When compared with CT_VD_WarmAcu, CT_VD_TFMox (SMD = −1.63, 95% CrI: −2.48~−0.79), CT_VD_TCM_Mox (SMD = −1.85, 95% CrI: −2.62~−1.08), CT_VD_TCM_Acu (SMD = −1.35, 95% CrI: −2.13~−0.58), CT_VD_TCM (SMD = −2.13, 95% CrI: −2.96~−1.30), CT_VD_Pat (SMD = −1.72, 95% CrI: −2.50~−0.93), CT_VD_Mox (SMD = −2.19, 95% CrI: −2.95~−1.42), and CT_VD (SMD = −2.30, 95% CrI: −2.97~−1.63) were associated with higher low back pain scores. Relative to the effect of CT_VD, the SMDs for low back pain score favored CT_VD_TFMox (SMD = −0.67, 95% CrI: −1.18~−0.16), CT_VD_TCM_Mox (SMD = −0.45, 95% CrI: −0.83~−0.07), CT_VD_TCM_Acu (SMD = −0.95, 95% CrI: −1.34~−0.55), and CT_VD_Pat (SMD = −0.58, 95% CrI: −0.98~−0.19). Compared to CT_VD_TCM_Acu, several interventions underperformed significantly in terms of low back pain score decrease, specifically CT_VD_TCM (SMD = −0.78, 95% CrI: −1.39~−0.16) and CT_VD_Mox (SMD = −0.83, 95% CrI: −1.37~−0.30). All effect estimates are detailed in **Figure 8F**. The global inconsistency test indicated no significant inconsistency (P = 0.532), and heterogeneity was low (I² = 4%). One closed loop (CT_VD–CT_VD_Mox–CT_VD_TCM) was identified in the network, and the corresponding 95% CrI crossed zero, indicating no significant loop-specific inconsistency. **Supplementary Material 6** (**Supplementary Figure 1.8**) offers the forest plot for global inconsistency, whereas the forest plot for the low back pain score is illustrated in **Supplementary Material 7** (**Supplementary Figure 2.8**).

#### 3.4.4 VAS score

**Figure 9:**
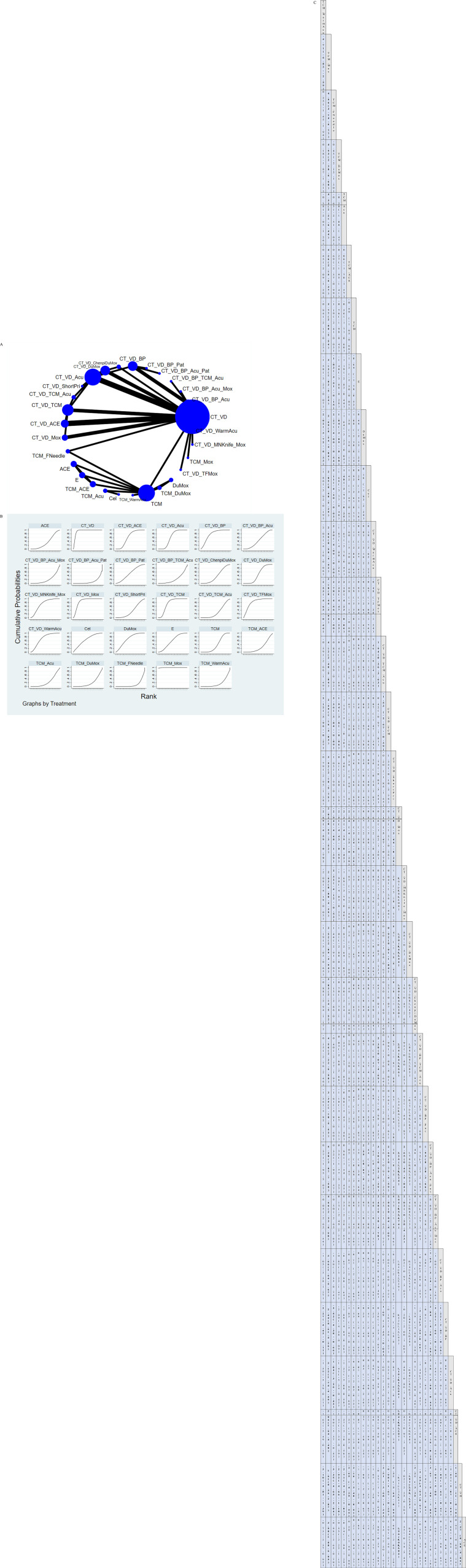
Network meta-analysis of the VAS score. **(A)** Network plot of the network meta-analysis for the VAS score. **(B)** Rankogram of overall intervention strategies from the network meta-analysis for the VAS score. **(C)** League table of the network meta-analysis for the VAS score.

Of the 53 studies that evaluated the effects of acupuncture combined with different pharmacological regimens on VAS score, 31 were included in the analysis. The intervention most often investigated was CT_VD, CT_VD_Acu and TCM were the next most often investigated. The direct comparison most commonly seen was CT_VD and CT_VD_Acu and comparisons between CT_VD and CT_VD_ACE. In total, 39 direct pairwise comparisons were generated across the set of included interventions. Closed loops existed in four sets of interventions: one comprising CT_VD, CT_VD_Mox, CT_VD_ACE, CT_VD_TCM, CT_VD_TCM_Acu, CT_VD_Acu, CT_VD_DuMox, CT_VD_ChenpiDuMox, the second comprising CT_VD, TCM, TCM_FNeedle, the third comprising TCM, DuMox, TCM_DuMox and the fourth containing TCM, E, ACE, TCM_ACE. In **Figure 9A** is shown the detailed network geometry.

The top three interventions for VAS score were CT_VD_BP_Acu_Pat (SUCRA = 9.7%), TCM_FNeedle (SUCRA = 12.3%) and TCM_WarmAcu (SUCRA = 17.4%), according to SUCRA values among the 29 evaluated. **Figure 9B** provides the overall ranking.

A clear advantage in VAS score was seen for TCM_WarmAcu (SMD = -4.71, 95% CrI: -6.83~-2.59), TCM_FNeedle (SMD = 4.96, 95% CrI: 3.14~6.77), TCM_DuMox (SMD = 4.53, 95% CrI: 2.38~6.68), TCM_Acu (SMD = 4.33, 95% CrI: 2.21~6.44), TCM_ACE (SMD = 4.26, 95% CrI: 2.15~6.37), TCM (SMD = 3.77, 95% CrI: 2~5.54), E (SMD = 3.13, 95% CrI: 1.02~5.24), DuMox (SMD = 2.69, 95% CrI: 0.54~4.84), Celecoxib capsules (Cel) (SMD = 2.85, 95% CrI: 0.4~5.29), CT_VD_WarmAcu (SMD = 2.66, 95% CrI: 0.9~4.42), CT_VD_TFMox (SMD = 2.18, 95% CrI: 0.44~3.92), CT_VD_TCM_Acu (SMD = 4.12, 95% CrI: 2.4~5.84), CT_VD_TCM (SMD = 2.59, 95% CrI: 1.16~4.03), CT_VD_ShortPri (SMD = 3.92, 95% CrI: 2.1~5.75), CT_VD_Mox (SMD = 2.02, 95% CrI: 0.52~3.52), CT_VD_MNKnife_Mox (SMD = 2.63, 95% CrI: 0.88~4.39), CT_VD_DuMox (SMD = 3.52, 95% CrI: 2.09~4.94), CT_VD_ChenpiDuMox (SMD = 3.83, 95% CrI: 2.14~5.52), CT_VD_BP_TCM_Acu (SMD = 4.56, 95% CrI: 2.22~6.9), CT_VD_BP_Pat (SMD = 3.35, 95% CrI: 1.18~5.53), CT_VD_BP_Acu_Pat (SMD = 5.28, 95% CrI: 3.07~7.5), CT_VD_BP_Acu_Mox (SMD = 4.66, 95% CrI: 2.33~6.98), CT_VD_BP_Acu (SMD = 3.66, 95% CrI: 1.64~5.67), CT_VD_BP (SMD = 2.44, 95% CrI: 0.61~4.26), CT_VD_Acu (SMD = 3.12, 95% CrI: 1.74~4.49), CT_VD_ACE (SMD = 3.06, 95% CrI: 1.64~4.48), CT_VD (SMD = 1.61, 95% CrI: 0.36~2.87), ACE (SMD = 3.98, 95% CrI: 1.88~6.09) over TCM_Mox. In terms of decreasing the VAS score, TCM_WarmAcu (SMD = -2.69, 95% CrI: -4.59~-0.79), TCM_FNeedle (SMD = -2.94, 95% CrI: -4.48~-1.39), TCM_DuMox (SMD = -2.51, 95% CrI: -4.43~-0.59), TCM_Acu (SMD = -2.31, 95% CrI: -4.19~-0.42), TCM_ACE (SMD = -2.24, 95% CrI: -4.13~-0.36), TCM (SMD = -1.75, 95% CrI: -3.24~-0.26), CT_VD_TCM_Acu (SMD = -2.1, 95% CrI: -3.47~-0.73) and CT_VD_ShortPri (SMD = -1.9, 95% CrI: -3.44~-0.36) all ranked above CT_VD_Mox. A clear advantage in lowering the VAS score was seen for TCM_WarmAcu (SMD = -3.1, 95% CrI: -4.81~-1.39), TCM_FNeedle (SMD = -3.35, 95% CrI: -4.65~-2.04), TCM_DuMox (SMD = -2.92, 95% CrI: -4.66~-1.18), TCM_Acu (SMD = -2.71, 95% CrI: -4.41~-1.01), TCM_ACE (SMD = -2.65, 95% CrI: -4.35~-0.95), TCM (SMD = -2.16, 95% CrI: -3.4~-0.91), CT_VD_TCM_Acu (SMD = -2.51, 95% CrI: -3.68~-1.34), CT_VD_TCM (SMD = -0.98, 95% CrI: -1.67~-0.29), CT_VD_ShortPri (SMD = -2.31, 95% CrI: -3.64~-0.99), CT_VD_DuMox (SMD = -1.91, 95% CrI: -2.58~-1.23), CT_VD_ChenpiDuMox (SMD = -2.21, 95% CrI: -3.35~-1.08), CT_VD_BP_TCM_Acu (SMD = -2.95, 95% CrI: -4.92~-0.97), CT_VD_BP_Acu_Pat (SMD = -3.67, 95% CrI: -5.49~-1.85), CT_VD_BP_Acu_Mox (SMD = -3.04, 95% CrI: -5~-1.09), CT_VD_BP_Acu (SMD = -2.04, 95% CrI: -3.62~-0.47), CT_VD_Acu (SMD = -1.51, 95% CrI: -2.07~-0.94), CT_VD_ACE (SMD = -1.45, 95% CrI: -2.11~-0.78) over CT_VD. TCM_WarmAcu (SMD = -2.27, 95% CrI: -4.43~-0.11), TCM_FNeedle (SMD = -2.52, 95% CrI: -4.38~-0.66), CT_VD_TCM_Acu (SMD = -1.69, 95% CrI: -3.33~-0.04), CT_VD_BP_TCM_Acu (SMD = -2.12, 95% CrI: -3.59~-0.66), CT_VD_BP_Acu_Pat (SMD = -2.84, 95% CrI: -4.1~-1.59), CT_VD_BP_Acu_Mox (SMD = -2.22, 95% CrI: -3.66~-0.78), CT_VD_BP_Acu (SMD = -1.22, 95% CrI: -2.07~-0.36) surpassed CT_VD_BP in their ability to lower VAS score. Multiple interventions proved significantly inferior to TCM_WarmAcu in lowering VAS score, including DuMox (SMD = -2.02, 95% CrI: -3.72~-0.33), CT_VD_TFMox (SMD = -2.53, 95% CrI: -4.63~-0.44), CT_VD_TCM (SMD = -2.12, 95% CrI: -3.96~-0.27). Taking TCM_FNeedle as the comparator, all other listed interventions showed significantly smaller VAS score improvements: E (SMD = -1.83, 95% CrI: -3.51~-0.15), DuMox (SMD = -2.27, 95% CrI: -4~-0.54), Cel (SMD = -2.11, 95% CrI: -4.2~-0.03), CT_VD_WarmAcu (SMD = -2.3, 95% CrI: -4.09~-0.5), CT_VD_TFMox (SMD = -2.78, 95% CrI: -4.56~-1), CT_VD_TCM (SMD = -2.36, 95% CrI: -3.84~-0.89), CT_VD_MNKnife_Mox (SMD = -2.32, 95% CrI: -4.12~-0.53), CT_VD_Acu (SMD = -1.84, 95% CrI: -3.26~-0.42). In contrast to TCM_DuMox, several interventions produced significantly less pronounced VAS score improvements, namely DuMox (SMD = -1.84, 95% CrI: -3.09~-0.6), CT_VD_TFMox (SMD = -2.35, 95% CrI: -4.47~-0.23), CT_VD_TCM (SMD = -1.94, 95% CrI: -3.81~-0.06). When benchmarked against TCM_Acu, the following interventions each yielded significantly smaller improvements in VAS score: Cel (SMD = -1.48, 95% CrI: -2.71~-0.25), CT_VD_TFMox (SMD = -2.15, 95% CrI: -4.23~-0.06). Relative to CT_VD_Acu, several interventions decreased VAS score to a greater extent: TCM_FNeedle (SMD = -1.84, 95% CrI: -3.26~-0.42), CT_VD_BP_Acu_Pat (SMD = -2.16, 95% CrI: -3.9~-0.43). By contrast, CT_VD_Mox (SMD = 1.1, 95% CrI: 0.13~2.06) resulted in a smaller decrease. When benchmarked against CT_VD_BP_Acu_Pat, the following interventions each yielded significantly smaller improvements in VAS score: DuMox (SMD = 2.59, 95% CrI: 0.07~5.12), CT_VD_TFMox (SMD = 3.1, 95% CrI: 0.92~5.29), CT_VD_TCM (SMD = 2.69, 95% CrI: 0.79~4.59), CT_VD_Mox (SMD = 3.26, 95% CrI: 1.28~5.24), CT_VD_MNKnife_Mox (SMD = 2.65, 95% CrI: 0.45~4.84), CT_VD_BP_Pat (SMD = 1.93, 95% CrI: 0.2~3.66), CT_VD_BP_Acu (SMD = -1.63, 95% CrI: -3.14~-0.11), CT_VD_Acu (SMD = -2.16, 95% CrI: -3.9~-0.43), CT_VD_ACE (SMD = -2.22, 95% CrI: -4.16~-0.29). Compared to CT_VD_TFMox, the three interventions —— CT_VD_TCM_Acu (SMD = 1.94, 95% CrI: 0.26~3.63), CT_VD_BP_TCM_Acu (SMD = 2.38, 95% CrI: 0.07~4.69), CT_VD_BP_Acu_Mox (SMD = 2.48, 95% CrI: 0.18~4.78) resulted in greater VAS score reduction. CT_VD_DuMox (SMD = 0.92, 95% CrI: 0.01~1.84), CT_VD_BP_Acu_Mox (SMD = 2.06, 95% CrI: 0.04~4.09), surpassed CT_VD_TCM in their ability to improve VAS score. Relative to CT_VD_Mox, CT_VD_DuMox (SMD = 1.5, 95% CrI: 0.45~2.54), CT_VD_ChenpiDuMox (SMD = 1.8, 95% CrI: 0.41~3.2), CT_VD_BP_TCM_Acu (SMD = 2.54, 95% CrI: 0.41~4.66), CT_VD_BP_Acu_Mox (SMD = 2.64, 95% CrI: 0.53~4.74), CT_VD_ACE (SMD = 1.04, 95% CrI: 0.01~2.07), ACE (SMD = 1.96, 95% CrI: 0.08~3.84) yielded more favorable VAS score changes. The data indicated that CT_VD_BP_Acu_Mox (SMD = 2.02, 95% CrI: -0.28~4.33) produced a superior VAS score response relative to CT_VD_MNKnife_Mox. When using CT_VD as the baseline, ACE (SMD = 2.37, 95% CrI: 0.68~4.06) stood out with greater improvements in VAS score. **Figure 9C** gives the detailed league table.

The global inconsistency test indicated no significant inconsistency (P = 0.071), and heterogeneity was low (I² = 2%). Five closed loops were identified within the network evidence structure, of which three loops (CT_VD–CT_VD_Acu–CT_VD_DuMox, CT_VD–CT_VD_Mox–CT_VD_TCM, and CT_VD–CT_VD_ACE–CT_VD_TCM) had 95% CrIs that included zero, indicating no significant loop inconsistency. The forest plot for global inconsistency is supplied in **Supplementary Material 6** (**Supplementary Figure 1.9**), while **Supplementary Material 7** (**Supplementary Figure 2.9**) features the forest plot for the VAS score.

#### 3.4.5 PINP

**Figure 10:**
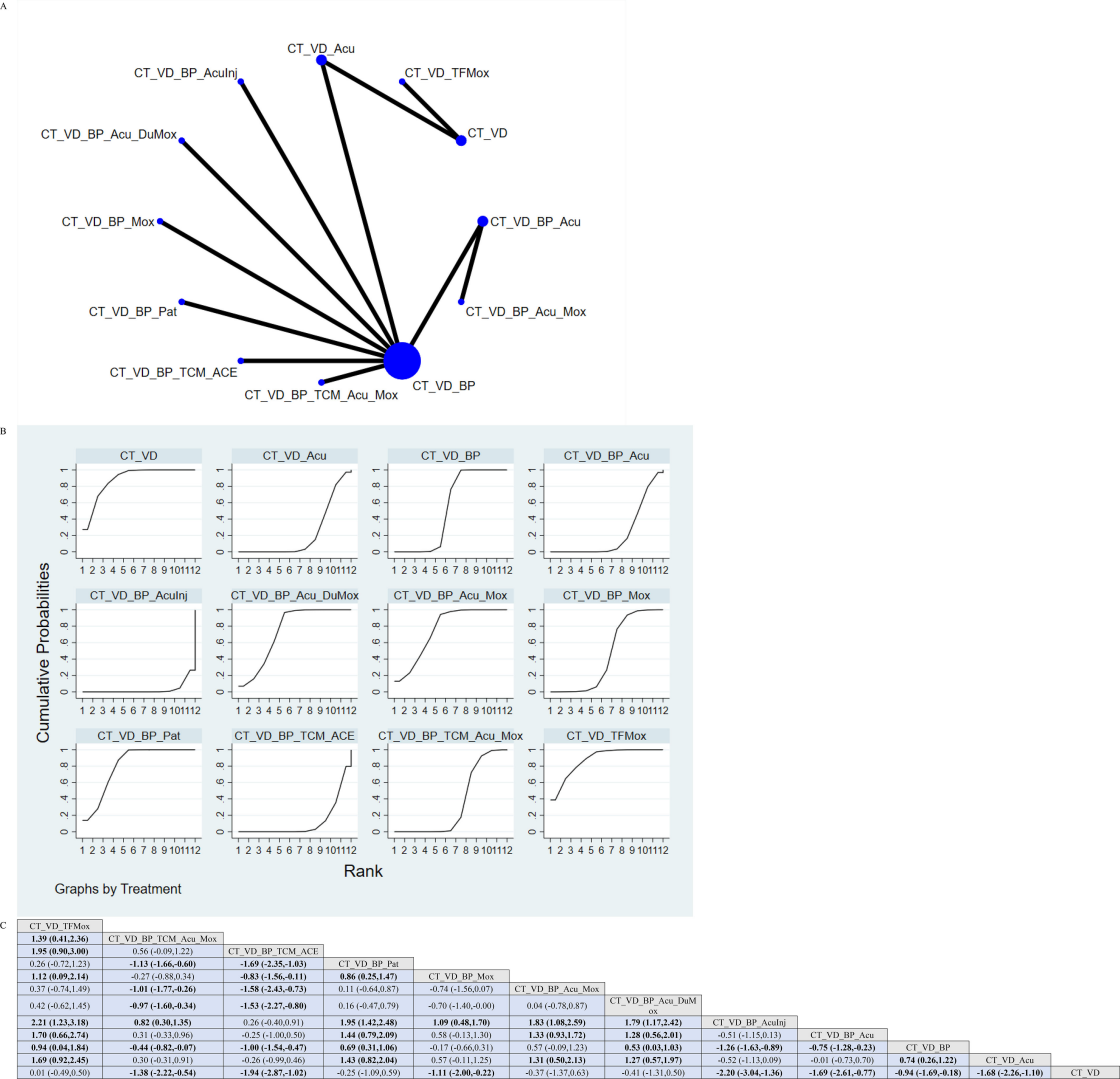
Network meta-analysis of PINP **(A)** Network plot of the network meta-analysis for PINP. **(B)** Rankogram of overall intervention strategies from the network meta-analysis for PINP. **(C)** League table of the network meta-analysis for PINP.

Eighteen studies investigated the effects of acupuncture combined with different pharmacological regimens on PINP, with 11 of them being included in the analysis. CT_VD_BP was the intervention with the highest investigation frequency, followed by CT_VD_Acu, CT_VD_BP _Acu and CT_VD. Each pairwise comparison was informed by a single study, resulting in 11 direct comparisons across the included interventions. The evidence network lacked any closed loops, meaning that no indirect comparisons existed. **Figure 10A** depicts the detailed network geometry.

When ranked by SUCRA values, CT_VD_BP_AcuInj emerged as the best intervention for PINP among the 12 assessed (SUCRA = 2.9%), followed by CT_VD_BP_TCM_ACE (SUCRA = 12%) and CT_VD_BP_Acu (SUCRA = 22.1%). The complete ranking can be found in **Figure 10B**.

In the league table, the data indicated that CT_VD_BP_TCM_Acu_Mox (SMD = -1.13, 95% CrI: -1.66~-0.60), CT_VD_BP_TCM_ACE (SMD = -1.69, 95% CrI: -2.35~-1.03), CT_VD_BP_Mox (SMD = 0.86, 95% CrI: 0.25~1.47), CT_VD_BP_AcuInj (SMD = 1.95, 95% CrI: 1.42~2.48), CT_VD_BP_Acu (SMD = 1.44, 95% CrI: 0.79~2.09), CT_VD_BP (SMD = 0.69, 95% CrI: 0.31~1.06) and CT_VD_Acu (SMD = 1.43, 95% CrI: 0.82~2.04) each produced a superior PINP reduction relative to CT_VD_BP_Pat. Relative to CT_VD_BP, a greater decrease in PINP was observed with CT_VD_BP_TCM_Acu_Mox (SMD = -0.44, 95% CrI: -0.82~-0.07), CT_VD_BP_TCM_ACE (SMD = -1.00, 95% CrI: -1.54~-0.47), CT_VD_BP_AcuInj (SMD = -1.26, 95% CrI: -1.63~-0.89), CT_VD_BP_Acu (SMD = -0.75, 95% CrI: -1.28~-0.23) and CT_VD_Acu (SMD = 0.74, 95% CrI: 0.26~1.22), whereas CT_VD_TFMox (SMD = 0.94, 95% CrI: 0.04~1.84), CT_VD_BP_Acu_DuMox (SMD = 0.53, 95% CrI: 0.03~1.03), CT_VD (SMD = -0.94, 95% CrI: -1.69~-0.18), exhibited a smaller decrease. When using CT_VD as the baseline, CT_VD_BP_TCM_Acu_Mox (SMD = -1.38, 95% CrI: -2.22~-0.54), CT_VD_BP_TCM_ACE (SMD = -1.94, 95% CrI: -2.87~-1.02), CT_VD_BP_Mox (SMD = -1.11, 95% CrI: -2~-0.22), CT_VD_BP_AcuInj (SMD = -2.20, 95% CrI: -3.04~-1.36), CT_VD_BP_Acu (SMD = -1.69, 95% CrI: -2.61~-0.77) and CT_VD_Acu (SMD = -1.68, 95% CrI: -2.26~-1.10) stood out with greater reduction in the PINP level. When compared with CT_VD_TFMox, CT_VD_BP_TCM_Acu_Mox (SMD = 1.39, 95% CrI: 0.41~2.36), CT_VD_BP_TCM_ACE (SMD = 1.95, 95% CrI: 0.90~3.00), CT_VD_BP_Mox (SMD = 1.12, 95% CrI: 0.09~2.14), CT_VD_BP_AcuInj (SMD = 2.21, 95% CrI: 1.23~3.18), CT_VD_BP_Acu (SMD = 1.70, 95% CrI: 0.66~2.74) and CT_VD_Acu (SMD = 1.69, 95% CrI: 0.92~2.45) led to superior reductions in PINP. Relative to CT_VD_BP_AcuInj, a significantly inferior PINP decrease was noted for CT_VD_BP_TCM_Acu_Mox (SMD = 0.82, 95% CrI: 0.3~1.35), CT_VD_BP_Mox (SMD = 1.09, 95% CrI: 0.48~1.7), CT_VD_BP_Acu_Mox (SMD = 1.83, 95% CrI: 1.08~2.59), CT_VD_BP_Acu_DuMox (SMD = 1.79, 95% CrI: 1.17~2.42). When using CT_VD_BP_Acu_Mox as the baseline, CT_VD_BP_TCM_Acu_Mox (SMD = -1.01, 95% CrI: -1.77~-0.26), CT_VD_BP_TCM_ACE (SMD = -1.58, 95% CrI: -2.43~-0.73), CT_VD_BP_Acu (SMD = 1.33, 95% CrI: 0.93~1.72), CT_VD_Acu (SMD = 1.31, 95% CrI: 0.5~2.13) stood out with greater reductions in PINP. When compared with CT_VD_BP_Acu_DuMox, CT_VD_BP_TCM_Acu_Mox (SMD = -0.97, 95% CrI: -1.6~-0.34), CT_VD_BP_TCM_ACE (SMD = -1.53, 95% CrI: -2.27~-0.8), CT_VD_BP_Acu (SMD = 1.28, 95% CrI: 0.56~2.01), CT_VD_Acu (SMD = 1.27, 95% CrI: 0.57~1.97) led to superior decrease in PINP. Compared to CT_VD_BP_TCM_ACE, CT_VD_BP_Mox (SMD = -0.83, 95% CrI: -1.56~-0.11) underperformed significantly in terms of PINP reduction. All effect estimates are summarized in **Figure 10C**.

The global inconsistency test indicated significant inconsistency within the network (P <0.001), although heterogeneity was low (I² = 4%). As no closed loops were formed in the evidence network, loop-specific inconsistency analyses were not performed. The forest plot for global inconsistency appears in **Supplementary Material 6** (**Supplementary Figure 1.10**); as for the forest plot for PINP, it is available in **Supplementary Material 7** (**Supplementary Figure 2.10**).

#### 3.4.6 CTX

**Figure 11:**
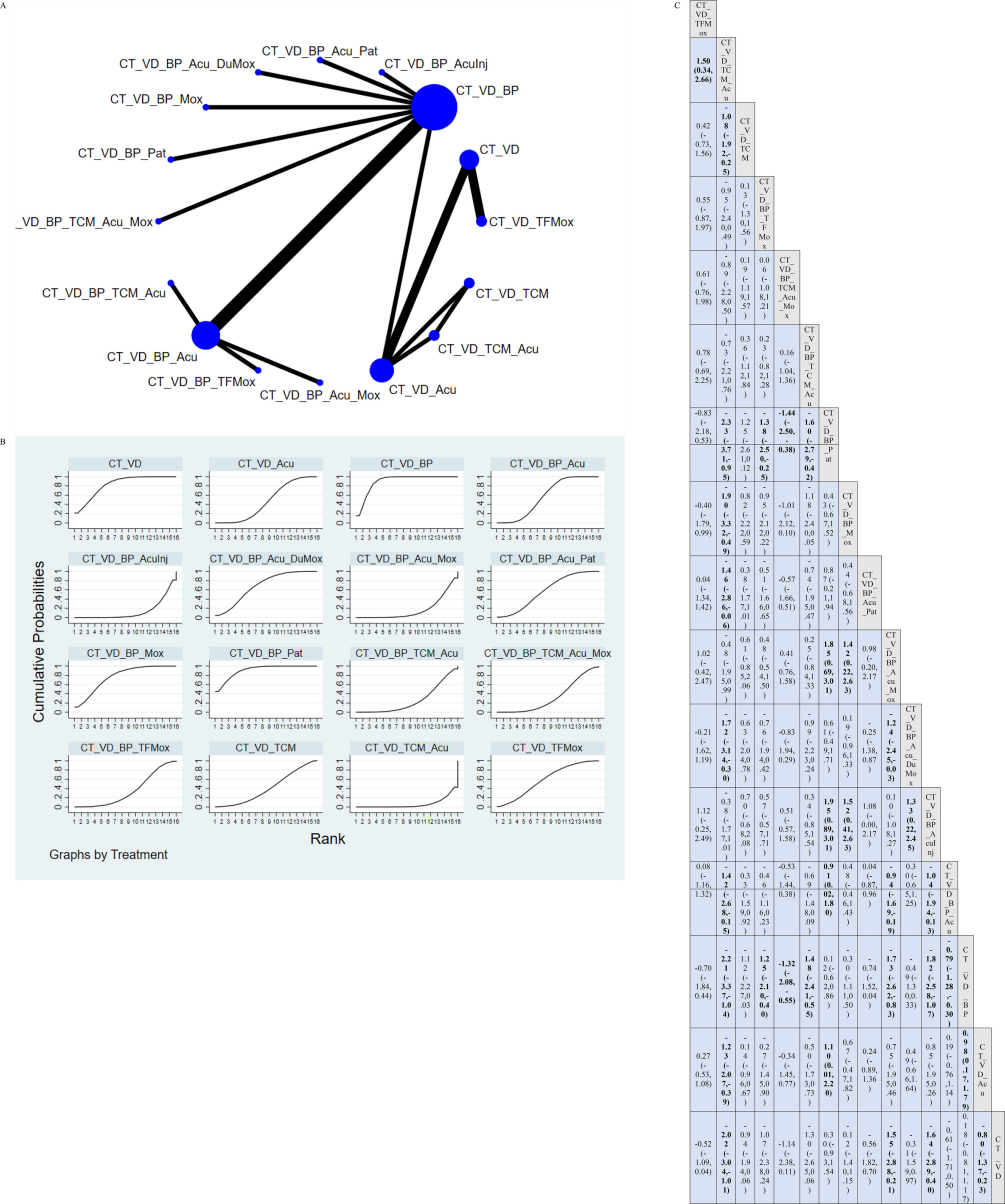
Network meta-analysis of CTX **(A)** Network plot of the network meta-analysis for CTX. **(B)** Rankogram of overall intervention strategies from the network meta-analysis for CTX. **(C)** League table of the network meta-analysis for CTX.

Eighteen out of 27 studies that assessed the effects of acupuncture combined with different pharmacological regimens on CTX were ultimately included in the analysis. In terms of how often interventions were investigated, CT_VD_BP was first, CT_VD_BP_Acu was second and CT_VD_Acu was third. The direct comparison that occurred most frequently was between CT_VD_BP and CT_VD_BP_Acu, followed by comparisons between CT_VD and CT_VD_TFMox, as well as between CT_VD and CT_VD_Acu. Overall, 16 direct pairwise comparisons arose from the included interventions. A Closed loop existed in CT_VD_Acu, CT_VD_TCM, and CT_VD_TCM_Acu, but it came from a single multi-arm study. The detailed network geometry is shown in **Figure 11A**.

When ranked by SUCRA values, CT_VD_TCM_Acu emerged as the best intervention for CTX among the 16 assessed (SUCRA = 6.5%), followed by CT_VD_BP_AcuInj (SUCRA = 13.6%) and CT_VD_BP_Acu_Mox (SUCRA = 17.2%). The complete ranking can be found in **Figure 11B**.

**Figure 12:**
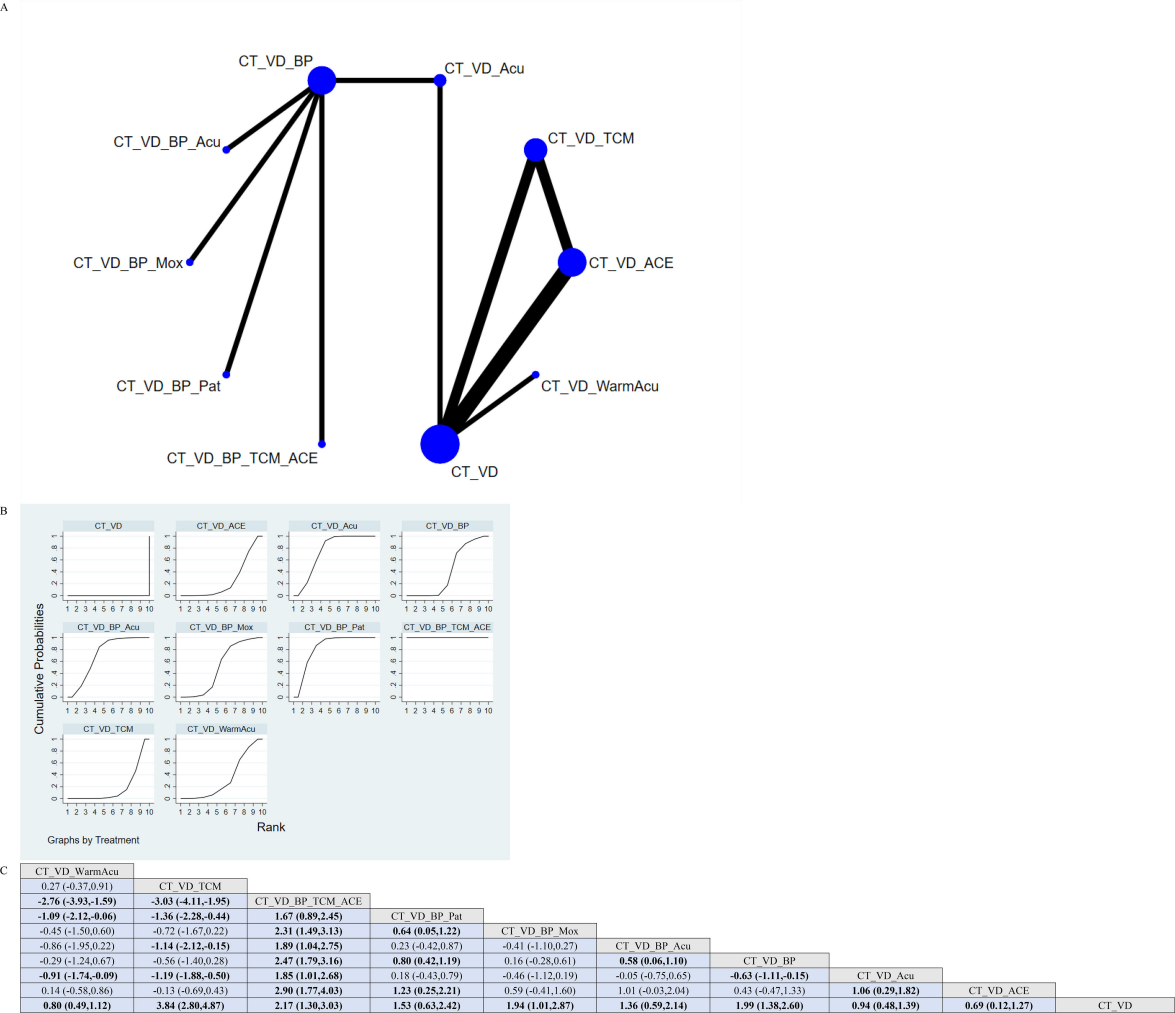
Network meta-analysis of E2 **(A)** Network plot of the network meta-analysis for E2. **(B)** Rankogram of overall intervention strategies from the network meta-analysis for E2. **(C)** League table of the network meta-analysis for E2.

When placed against CT_VD_TCM_Acu, the remaining interventions demonstrated significantly less reduction in CTX: CT_VD_TFMox (SMD = 1.5, 95% CrI: 0.34~2.66), CT_VD_TCM (SMD = -1.08, 95% CrI: -1.92~-0.25), CT_VD_BP_Pat (SMD = -2.33, 95% CrI: -3.71~-0.95), CT_VD_BP_Mox (SMD = -1.9, 95% CrI: -3.32~-0.49), CT_VD_BP_Acu_Pat (SMD = -1.46, 95% CrI: -2.86~-0.06), CT_VD_BP_Acu_DuMox (SMD = -1.72, 95% CrI: -3.14~-0.3), CT_VD_BP_Acu (SMD = -1.42, 95% CrI: -2.68~-0.15), CT_VD_BP (SMD = -2.21, 95% CrI: -3.37~-1.04), CT_VD_Acu (SMD = -1.23, 95% CrI: -2.07~-0.39), CT_VD (SMD = -2.02, 95% CrI: -3.04~-1.01). CT_VD_BP_TFMox (SMD = -1.38, 95% CrI: -2.5~-0.25), CT_VD_BP_TCM_Acu_Mox (SMD = -1.44, 95% CrI: -2.5~-0.38), CT_VD_BP_TCM_Acu (SMD = -1.6, 95% CrI: -2.79~-0.42), CT_VD_BP_Acu_Mox (SMD = 1.85, 95% CrI: 0.69~3.01), CT_VD_BP_AcuInj (SMD = 1.95, 95% CrI: 0.89~3.01), CT_VD_BP_Acu (SMD = 0.91, 95% CrI: 0.02~1.8), CT_VD_Acu (SMD = 1.1, 95% CrI: 0.01~2.2) all outperformed CT_VD_BP_Pat in terms of CTX decrease. Relative to CT_VD_BP, CT_VD_BP_TFMox (SMD = -1.25, 95% CrI: -2.1~-0.4), CT_VD_BP_TCM_Acu_Mox (SMD = -1.32, 95% CrI: -2.08~-0.55), CT_VD_BP_TCM_Acu (SMD = -1.48, 95% CrI: -2.41~-0.55), CT_VD_BP_Acu_Mox (SMD = -1.73, 95% CrI: -2.62~-0.83), CT_VD_BP_AcuInj (SMD = -1.82, 95% CrI: -2.58~-1.07), CT_VD_BP_Acu (SMD = -0.79, 95% CrI: -1.28~-0.3), CT_VD_Acu (SMD = 0.98, 95% CrI: 0.17~1.79) showed greater effectiveness in lowering CTX. Relative to CT_VD_BP_Acu_Mox, a significantly inferior CTX outcome was noted for multiple interventions, including CT_VD_BP_Mox (SMD = 1.42, 95% CrI: 0.22~2.63), CT_VD_BP_Acu_DuMox (SMD = -1.24, 95% CrI: -2.45~-0.03), CT_VD_BP_Acu (SMD = -0.94, 95% CrI: -1.69~-0.19), CT_VD (SMD = -1.55, 95% CrI: -2.88~-0.21). When placed against CT_VD_BP_AcuInj, the remaining interventions demonstrated significantly less improvement in CTX: CT_VD_BP_Mox (SMD = 1.52, 95% CrI: 0.41~2.63), CT_VD_BP_Acu_DuMox (SMD = 1.33, 95% CrI: 0.22~2.45), CT_VD_BP_Acu (SMD = -1.04, 95% CrI: -1.94~-0.13), CT_VD (SMD = -1.64, 95% CrI: -2.89~-0.4). A higher CTX decrease was observed for CT_VD_Acu (SMD = -0.80, 95% CrI: -1.37~-0.23) than for CT_VD. **Figure 12C** presents al effect estimates.

The global inconsistency test indicated no significant inconsistency across the network (P = 0.192) and heterogeneity was negligible (I² = 25%). Although one closed loop was identified, it originated from a single multi-arm study; therefore, loop-specific inconsistency analysis was not conducted. The forest plot for global inconsistency is reported in **Supplementary Material 6** (**Supplementary Figure 1.11**). The forest plot for CTX can be seen in **Supplementary Material 7** (**Supplementary Figure 2.11**).

#### 3.4.7 E2

A total of 26 studies were conducted to evaluate the effects of acupuncture combined with different pharmacological regimens on E2, and of these, 10 were included in the analysis. CT_VD was the most commonly investigated intervention, with CT_VD_BP and CT_VD_ACE being the second most common. The most frequent direct comparison involved CT_VD and CT_VD_ACE, followed by CT_VD versus CT_VD_TCM and CT_VD_TCM versus CT_VD_ACE. A total of 10 direct pairwise comparisons were generated from the included interventions. A closed loop was identified among CT_VD, CT_VD_ACE, CT_VD_TCM, which indicates the existence of indirect evidence. Refer to **Figure 12A** for the detailed network geometry.

Among the 10 interventions evaluated, CT_VD_BP_TCM_ACE exhibited the highest SUCRA value for E2 improvement (SUCRA = 100%), ahead of CT_VD_BP_Pat (SUCRA = 82.5%) and CT_VD_Acu (SUCRA = 74.7%). See **Figure 12B** for the overall ranking.

A significantly smaller E2 increase was achieved by several interventions compared with CT_VD_BP_TCM_ACE, namely CT_VD_WarmAcu (SMD = -2.76, 95% CrI: -3.93~-1.59), CT_VD_TCM (SMD = -3.03, 95% CrI: -4.11~-1.95), CT_VD_BP_Pat (SMD = 1.67, 95% CrI: 0.89~2.45), CT_VD_BP_Mox (SMD = 2.31, 95% CrI: 1.49~3.13), CT_VD_BP_Acu (SMD = 1.89, 95% CrI: 1.04~2.75), CT_VD_BP (SMD = 2.47, 95% CrI: 1.79~3.16), CT_VD_Acu (SMD = 1.85, 95% CrI: 1.01~2.68), CT_VD_ACE (SMD = 2.90, 95% CrI: 1.77~4.03) and CT_VD (SMD = 2.17, 95% CrI: 1.30~3.03). In head to head comparisons with CT_VD_BP_Pat, multiple interventions yielded significantly smaller E2 gains, including CT_VD_WarmAcu (SMD = -1.09, 95% CrI: -2.12~-0.06), CT_VD_TCM (SMD = -1.36, 95% CrI: -2.28~-0.44), CT_VD_BP_Mox (SMD = 0.64, 95% CrI: 0.05~1.22), CT_VD_BP (SMD = 0.80, 95% CrI: 0.42~1.19), CT_VD_ACE (SMD = 1.23, 95% CrI: 0.25~2.21) and CT_VD (SMD = 1.53, 95% CrI: 0.63~2.42). Relative to the effect of CT_VD_BP_Acu, the following interventions each produced a significantly smaller E2 improvement: CT_VD_TCM (SMD = -1.14, 95% CrI: -2.12~-0.15), CT_VD_BP (SMD = 0.58, 95% CrI: 0.06~1.10) and CT_VD (SMD = 1.36, 95% CrI: 0.59~2.14). Several interventions showed significantly poorer E2 outcomes than CT_VD_Acu, specifically CT_VD_WarmAcu (SMD = -0.91, 95% CrI: -1.74~-0.09), CT_VD_TCM (SMD = -1.19, 95% CrI: -1.88~-0.50), CT_VD_BP (SMD = -0.63, 95% CrI: -1.11~-0.15), CT_VD_ACE (SMD = 1.06, 95% CrI: 0.29~1.82) and CT_VD (SMD = 0.94, 95% CrI: 0.48~1.39). Compared with the reference (CT_VD), CT_VD_WarmAcu (SMD = 0.80, 95% CrI: 0.49~1.12), CT_VD_TCM (SMD = 3.84, 95% CrI: 2.80~4.87), CT_VD_BP_Mox (SMD = 1.94, 95% CrI: 1.01~2.87), CT_VD_BP (SMD = 1.99, 95% CrI: 1.38~2.60) and CT_VD_ACE (SMD = 0.69, 95% CrI: 0.12~1.27) yielded more pronounced improvements in E2.

The global inconsistency test indicated significant inconsistency across the network (P = 0.017), and heterogeneity was low (I² = 6%). Additionally, one closed loop (CT_VD–CT_VD_TCM–CT_VD_ACE) demonstrated significant loop-specific inconsistency, as the corresponding 95% CrI did not include zero; therefore, results involving this loop should be interpreted with caution. Two forest plots are presented in the **Supplementary Materials**: one for global inconsistency (**Supplementary Material 6**; **Supplementary Figure 1.12**) and another for E2 (**Supplementary Material 7**; **Supplementary Figure 2.12**).

#### 3.4.8 ALP

The effects of acupuncture combined with different pharmacological regimens on ALP were evaluated in 20 studies. Of these, 15 were included in the analysis: 7 studies did not contain BP, and 8 studies contained BP.

**Figure 13:**
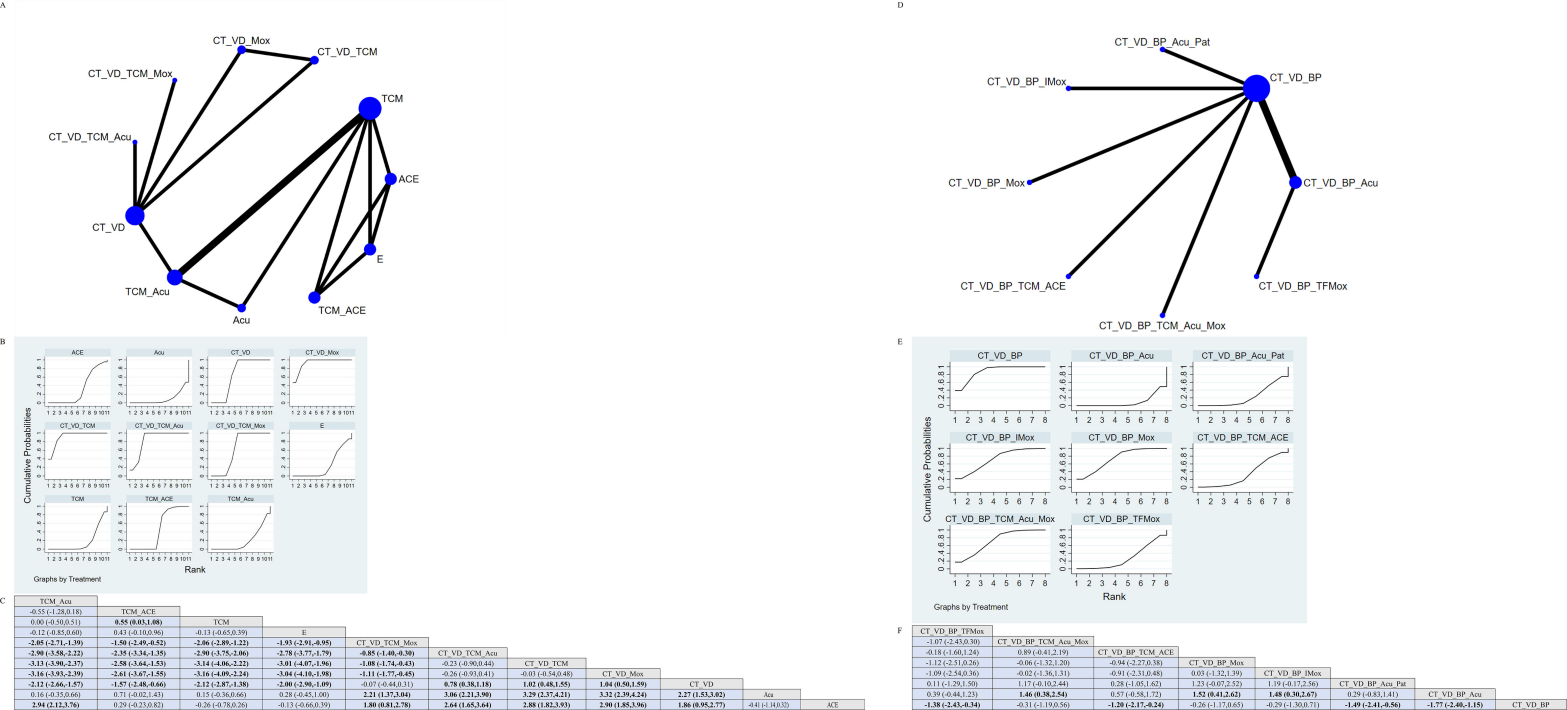
Network meta-analysis of ALP **(A)** Network plot of the network meta-analysis for ALP (exclude BP). **(B)** Rankogram of overall intervention strategies from the network meta-analysis for ALP (exclude BP). **(C)** League table of the network meta-analysis for ALP (exclude BP). **(D)** Network plot of the network meta-analysis for ALP (include BP). **(E)** Rankogram of overall intervention strategies from the network meta-analysis for ALP (include BP). **(F)** League table of the network meta-analysis for ALP (include BP).

When interventions did not include BP, the most frequently studied intervention was TCM, followed in frequency by CT_VD and TCM_Acu. The direct comparison that appeared most often was that between TCM and TCM_Acu. In total, 15 direct pairwise comparisons were generated across the included interventions. The presence of indirect evidence was indicated by closed loops identified among CT_VD–CT_VD_TCM–CT_VD_Mox, TCM–TCM_Acu–Acu, and TCM–ACE–E–TCM_ACE. Detailed network geometry is presented in **Figure 13A**. In terms of SUCRA values, CT_VD_Mox was the top ranked intervention for enhancing ALP among the 11 evaluated (SUCRA = 93.2%), with CT_VD_TCM (SUCRA = 92.2%) and CT_VD_TCM_Acu (SUCRA = 84.6%) following. The overall ranking is illustrated in **Figure 13B**.

We observed a greater increase in ALP with CT_VD_TCM_Acu (SMD =-0.85, 95% CrI: -1.40~-0.30), CT_VD_TCM (SMD = -1.08, 95% CrI: -1.74~-0.43), CT_VD_Mox(SMD = -1.11, 95% CrI: -1.77~-0.45) relative to CT_VD_TCM_Mox, while TCM_Acu (SMD = -2.05, 95% CrI: -2.71~-1.39), TCM_ACE (SMD =-1.50, 95% CrI: -2.49~-0.52), TCM (SMD = -2.06, 95% CrI: -2.89~-1.22), E (SMD = -1.93, 95% CrI: -2.91~-0.95), Acu (SMD = 2.21, 95% CrI: 1.37~3.04) and ACE (SMD = 1.08, 95% CrI: 0.81~2.78) showed a smaller increase. When using CT_VD_TCM_Acu as the baseline, multiple interventions resulted in significantly smaller ALP enhancements, namely TCM_Acu (SMD = -2.90, 95% CrI: -3.58~-2.22), TCM_ACE (SMD = -2.35, 95% CrI: -3.34~-1.35), TCM (SMD = -2.90, 95% CrI: -3.75~-2.06), E (SMD = -2.78, 95% CrI: -3.77~-1.79), CT_VD (SMD = 0.78, 95% CrI: 0.38~1.18), Acu (SMD = 3.06, 95% CrI: 2.21~3.90) and ACE (SMD = 2.64, 95% CrI: 1.65~3.64). TCM_ACE (SMD = 0.55, 95% CrI: 0.03~1.08) was associated with larger ALP increases relative to TCM. In head to head comparisons against ACE, TCM_Acu (SMD = 2.94, 95% CrI: 2.12~3.76), CT_VD_TCM (SMD = 2.88, 95% CrI: 1.82~3.93), CT_VD_Mox (SMD = 2.90, 95% CrI: 1.85~3.96), and CT_VD (SMD = 1.86, 95% CrI: 0.95~2.77) demonstrated superior ALP outcomes. A significantly less favorable ALP response was observed for several interventions relative to CT_VD_TCM, including TCM_Acu (SMD =-3.13, 95% CrI: -3.90~-2.37), TCM_ACE (SMD =-2.58, 95% CrI: -3.64~-1.53), TCM (SMD =-3.14, 95% CrI: -4.06~-2.22), E (SMD =-3.01, 95% CrI: -4.07~-1.96), CT_VD (SMD = 1.02, 95% CrI: 0.48~1.55) and Acu (SMD = 3.29, 95% CrI: 2.37~4.21). Compared with CT_VD_Mox, the ALP improvement was significantly smaller for multiple interventions, namely TCM_Acu (SMD = -3.16, 95% CrI: -3.93~-2.39), TCM_ACE (SMD = -2.61, 95% CrI: -3.67~-1.55), TCM (SMD = -3.16, 95% CrI: -4.09~-2.24), E (SMD = -3.04, 95% CrI: -4.10~-1.98), CT_VD (SMD = 1.04, 95% CrI: 0.50~1.59) and Acu (SMD = 3.32, 95% CrI: 2.39~4.24). In contrast to CT_VD, a significantly smaller ALP gain was observed for the following interventions: TCM_Acu (SMD =-2.12, 95% CrI: -2.66~-1.57), TCM_ACE (SMD =-1.57, 95% CrI: -2.48~-0.66), TCM (SMD =-2.12, 95% CrI: -2.87~-1.38), E (SMD =-2.00, 95% CrI: -2.90~-1.09) and Acu (SMD = 2.27, 95% CrI: 1.53~3.02). All effect estimates are summarized in **Figure 13C**. The global inconsistency test revealed significant inconsistency across the network (P < 0.001), although heterogeneity was low (I² = 7%). One loop (TCM_Acu–Acu–TCM) exhibited significant loop-specific inconsistency, as the corresponding 95% CrI did not include zero. The **Supplementary Materials** include two forest plots: the former (global inconsistency) is found in **Supplementary Material 6** (**Supplementary Figure 1.13**), and the latter (BP-containing ALP) is provided in **Supplementary Material 7** (**Supplementary Figure 2.13**).

When interventions include BP, CT_VD_BP was the most frequently studied intervention, with CT_VD_BP_Acu ranking second. The most common direct comparison was between CT_VD_BP and CT_VD_BP_Acu. A total of seven direct pairwise comparisons were generated among the included interventions. No closed loops were found in the evidence network, implying the absence of indirect comparisons. The detailed structure of the network is illustrated in **Figure 13D**. The overall ranking of the eight interventions for ALP improvement is presented in **Figure 13E**. Based on SUCRA values, CT_VD_BP_Acu ranked highest (SUCRA = 9.3%), followed by CT_VD_BP_Acu_Pat (SUCRA = 22.6%) and CT_VD_BP_TFMox (SUCRA = 28.1%).

Relative to CT_VD_BP_Acu, several interventions yielded significantly less decrease in ALP, namely CT_VD_BP_TCM_Acu_Mox (SMD = 1.46, 95% CrI: 0.38~-2.54), CT_VD_BP_Mox (SMD = 1.52, 95% CrI: 0.41~2.62), CT_VD_BP_Indirect moxibustion (IMox) (SMD = 1.48, 95% CrI: 0.30~2.67). The ALP reduction was significantly greater with CT_VD_BP_TFMox (SMD =-1.38, 95% CrI: -2.46~-0.34), CT_VD_BP_TCM_ACE (SMD =-1.20, 95% CrI: -2.17~-0.24), CT_VD_BP_Acu_Pat (SMD =-1.49, 95% CrI: -2.41~-0.56) and CT_VD_BP_Acu (SMD =-1.77, 95% CrI: -2.40~-1.15) than with CT_VD_BP. All effect estimates are summarized in **Figure 13F**. The global inconsistency test indicated no significant inconsistency (P = 0.356), and heterogeneity was low (I² = 5%). As no closed loops were present in the evidence network, loop-specific inconsistency testing was not performed. The **Supplementary Materials** include two forest plots: the former (global inconsistency) is found in **Supplementary Material 6** (**Supplementary Figure 1.14**), and the latter (the BP-containing ALP) is provided in **Supplementary Material 7** (**Supplementary Figure 2.14**).

#### 3.4.9 OCN

Following evaluation of the effects of acupuncture combined with different pharmacological regimens on OCN in 18 studies, 14 were included in the analysis. Among them, 7 studies did not contain BP, 7 studies contain BP.

**Figure 14:**
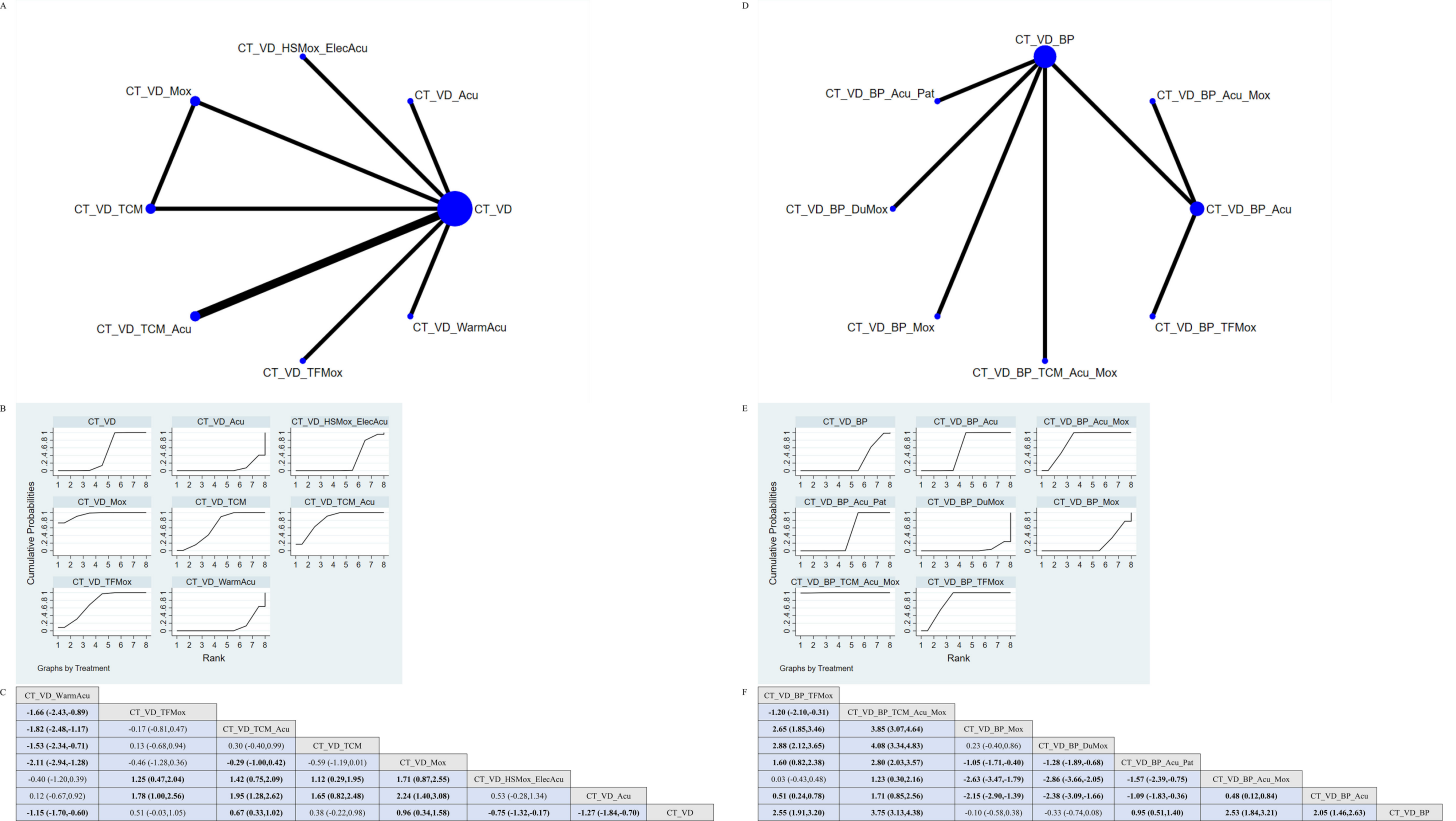
Network meta-analysis of OCN **(A)** Network plot of the network meta-analysis for ALP (exclude BP). **(B)** Rankogram of overall intervention strategies from the network meta-analysis for ALP (exclude BP). **(C)** League table of the network meta-analysis for ALP (exclude BP). **(D)** Network plot of the network meta-analysis for ALP (include BP). **(E)** Rankogram of overall intervention strategies from the network meta-analysis for ALP (include BP). **(F)** League table of the network meta-analysis for ALP (include BP).

For the BP-free interventions, CT_VD was the intervention investigated most often. The direct comparison between CT_VD and CT_VD_TCM_Acu was the most frequent. In total, eight direct pairwise comparisons were generated from the included interventions. Because no closed loops were detected in the evidence network except a multi-arm trial, indirect comparisons were absent. See **Figure 14A** for the detailed network geometry. The highest SUCRA value for OCN improvement among the 8 interventions was obtained by CT_VD_Mox (94.6%), followed by CT_VD_TCM_Acu (81.5%) and CT_VD_TFMox (72.1%). The overall ranking is shown in **Figure 14B**.

CT_VD_TFMox (SMD = -1.66, 95% CrI: -2.43~-0.89), CT_VD_TCM_Acu (SMD = -1.82, 95% CrI: -2.48~-1.17), CT_VD_TCM (SMD = -1.53, 95% CrI: -2.34~-0.71), CT_VD_Mox (SMD = -2.11, 95% CrI: -2.94~-1.28), CT_VD (SMD = -1.15, 95% CrI: -1.7~-0.6) all outperformed CT_VD_BP in terms of OCN improvement. Relative to CT_VD_Heat−sensitive moxibustion (HSMox)_ElecAcu, CT_VD_TFMox (SMD = 1.25, 95% CrI: 0.47~2.04), CT_VD_TCM_Acu (SMD = 1.42, 95% CrI: 0.75~2.09), CT_VD_TCM (SMD = 1.12, 95% CrI: 0.29~1.95), CT_VD_Mox (SMD = 1.71, 95% CrI: 0.87~2.55), CT_VD (SMD = -0.75, 95% CrI: -1.32~-0.17) showed greater effectiveness in enhancing OCN. A higher OCN increment was observed for CT_VD_TFMox (SMD = 1.78, 95% CrI: 1~2.56), CT_VD_TCM_Acu (SMD = 1.95, 95% CrI: 1.28~2.62), CT_VD_TCM (SMD = 1.65, 95% CrI: 0.82~2.48), CT_VD_Mox (SMD = 2.24, 95% CrI: 1.4~3.08), CT_VD (SMD = -1.27, 95% CrI: -1.84~-0.7) than for CT_VD_Acu. Compared with the reference (CT_VD), CT_VD_TCM_Acu (SMD = 0.67, 95% CrI: 0.33~1.02), CT_VD_Mox (SMD = 0.96, 95% CrI: 0.34~1.58) yielded more pronounced improvements in OCN. CT_VD_Mox (SMD = -0.29, 95% CrI: -1~0.42) was associated with larger OCN increases relative to CT_VD_TCM_Acu. **Figure 14C** offers the league table. The global inconsistency test yielded a significant result (P<0.001). Overall heterogeneity was negligible (I² = 7%). Loop specific inconsistency was not evaluated because the network contained no closed loops. In **Supplementary Material 6** (**Supplementary Figure 1.15**) is shown the forest plot for global inconsistency, and in **Supplementary Material 7** (**Supplementary Figure 2.15**) is presented the forest plot for OCN (exclude BP).

For the BP-containing interventions, among all interventions, CT_VD_BP had the highest frequency of investigation, followed by CT_VD_BP_Acu. Overall, seven direct pairwise comparisons were produced across the included interventions. The absence of closed loops in the evidence network suggested that there were no indirect comparisons. Detailed network geometry is presented in **Figure 14D**. A ranking based on SUCRA values placed CT_VD_BP_TCM_Acu_Mox first for OCN improvement (99.9%), CT_VD_BP_TFMox second (79.3%), and CT_VD_BP_Acu_Mox third (77.9%) among the eight interventions evaluated. See **Figure 14E** for the overall ranking.

In comparison to CT_VD_BP_TFMox, CT_VD_BP_TCM_Acu_Mox (SMD = -1.2, 95% CrI: -2.1~-0.31) showed greater OCN increases, whereas CT_VD_BP_Mox (SMD = 2.65, 95% CrI: 1.85~3.46), CT_VD_BP_DuMox (SMD = 2.88, 95% CrI: 2.12~3.65), CT_VD_BP_Acu_Pat (SMD = 1.6, 95% CrI: 0.82~2.38), CT_VD_BP_Acu (SMD = 0.51, 95% CrI: 0.24~0.78), CT_VD_BP (SMD = 2.55, 95% CrI: 1.91~3.2) showed a lesser increase. A significantly smaller OCN increase was achieved by several interventions compared with CT_VD_BP_TCM_Acu_Mox, namely CT_VD_BP_Mox (SMD = 3.85, 95% CrI: 3.07~4.64), CT_VD_BP_DuMox (SMD = 4.08, 95% CrI: 3.34~4.83), CT_VD_BP_Acu_Pat (SMD = 2.8, 95% CrI: 2.03~3.57), CT_VD_BP_Acu_Mox (SMD = 1.23, 95% CrI: 0.3~2.16), CT_VD_BP_Acu (SMD = 1.71, 95% CrI: 0.85~2.56), CT_VD_BP (SMD = 3.75, 95% CrI: 3.13~4.38). While a greater increase in OCN relative to CT_VD_BP_Acu_Pat was observed for CT_VD_BP_Acu_Mox (SMD = -1.57, 95% CrI: -2.39~-0.75), CT_VD_BP_Acu (SMD = -1.09, 95% CrI: -1.83~-0.36), a smaller increase was observed for CT_VD_BP_Mox (SMD = -1.05, 95% CrI: -1.71~-0.4), CT_VD_BP_DuMox (SMD = -1.28, 95% CrI: -1.89~-0.68), CT_VD_BP (SMD = 0.95, 95% CrI: 0.51~1.4). In head to head comparisons with CT_VD_BP_Acu_Mox, multiple interventions yielded significantly smaller OCN gains, including CT_VD_BP_Mox (SMD = -2.63, 95% CrI: -3.47~-1.79), CT_VD_BP_DuMox (SMD = -2.86, 95% CrI: -3.66~-2.05), CT_VD_BP_Acu (SMD = 0.48, 95% CrI: 0.12~0.84), CT_VD_BP (SMD = 2.53, 95% CrI: 1.84~3.21). Relative to the effect of CT_VD_BP_Acu, the following interventions each produced a significantly smaller OCN improvement: CT_VD_BP_Mox (SMD = -2.15, 95% CrI: -2.9~-1.39), CT_VD_BP_DuMox (SMD = -2.38, 95% CrI: -3.09~-1.66), CT_VD_BP (SMD = 2.05, 95% CrI: 1.46~2.63). All effect estimates are summarized in **Figure 14F**. Given that no closed loops existed within the network, we did not assess loop specific inconsistency. The global inconsistency test revealed significant inconsistency (P <0.001), and overall heterogeneity was low (I² = 7%). The forest plot for global inconsistency is detailed in **Supplementary Material 6** (**Supplementary Figure 1.16**), whereas **Supplementary Material 7** (**Supplementary Figure 2.16**) outlines the forest plot for OCN.

### 3.5 Comparison-adjusted funnel plots

**Figure 15:**
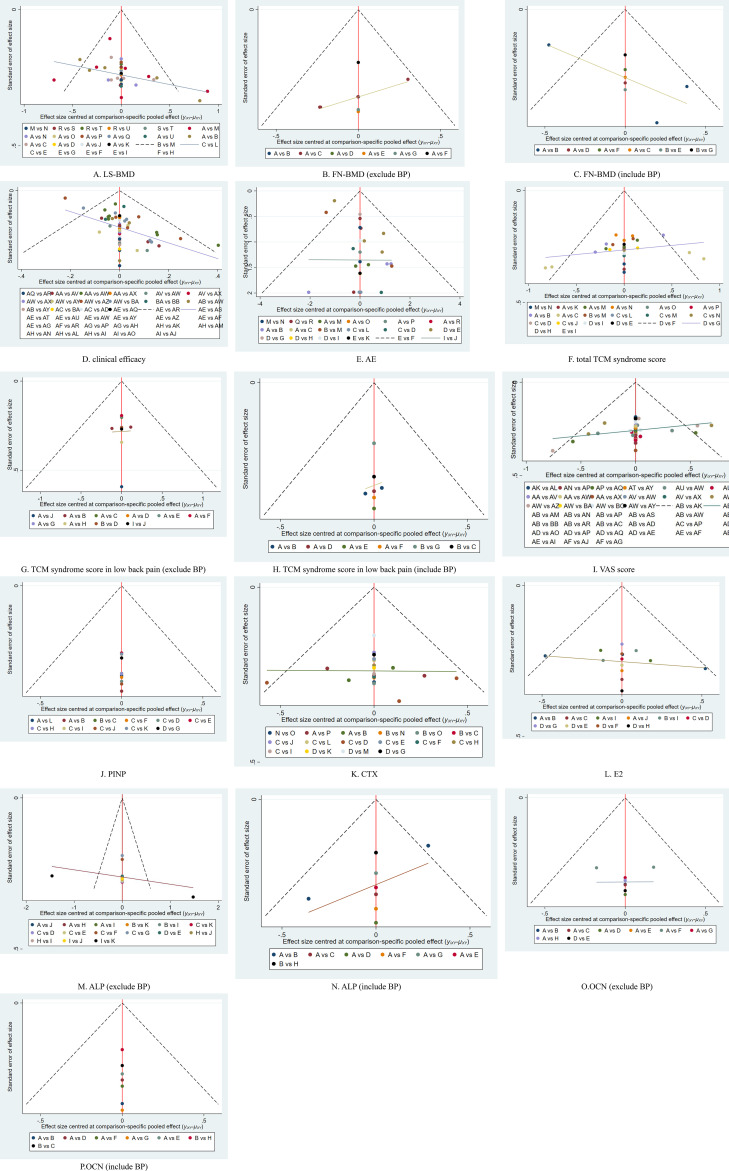
Adjusted funnel plots.

The risk of small-study effects and potential publication bias was assessed using comparison-adjusted funnel plots (**Figure 15**). Visual inspection showed that the funnel plots for all included outcomes were approximately symmetrical around the vertical axis, with no evident asymmetry. These findings suggest that there was no substantial evidence of small-study effects or publication bias among the included studies.

## 4 Discussion

This study included a total of 112 RCTs. We found that the optimal regimen for PMOP using acupuncture combined with various medications varies depending on the skeletal site, BP use, and outcome measure. Cal_ElecAcu demonstrated outstanding efficacy in improving LS-BMD. When interventions included or excluded BP, CT_VD_TCM_FDMox and CT_VD_BP_TCM_Acu, respectively, showed the greatest advantage in increasing FN-BMD. This suggests that the optimal physical stimulation (electroacupuncture, fire moxibustion, and traditional acupuncture) combined with a pharmacological foundation may differ by skeletal site. In terms of improving clinical symptoms and pain, TCM_Acu, TCM_WarmAcu, and CT_VD_DuMox led in increasing the clinical efficacy rate, TCM_Acu and CT_VD_BP_Acu_Mox stood out in alleviating low back pain symptoms, while CT_VD_BP_Acu_Pat demonstrated significant efficacy in reducing VAS scores. These findings highlight the core value of non-pharmacological therapies (such as warm acupuncture, Du meridian moxibustion, and acupoint plaster application) in symptom management. In terms of regulating bone metabolism and endocrine levels, CT_VD_TCM_Acu, CT_VD_BP_Acupoint injection (AcuInj), and CT_VD_BP_TCM_ACE were most effective in reducing CTX, lowering PINP, and increasing E2, respectively. Additionally, CT_VD_Mox and CT_VD_BP_TCM_Acu_Mox showed the strongest effects in elevating OCN levels, regardless of whether the intervention included BP or not.

Notably, in the reduction of serum PINP levels, the top-ranked interventions were all centered on CT_VD_BP and combined with adjunctive modalities such as acupoint injection, Chinese herbal medicine, or conventional acupuncture (e.g., CT_VD_BP_AcuInj). This finding is closely related to the potent anti-osteoporotic effects of bisphosphonates. Under the physiological “coupling” mechanism of bone remodeling, substantial inhibition of bone resorption is typically accompanied by a compensatory reduction in bone formation, which is reflected by decreased PINP levels (130).

In this study, the results for ALP revealed an intriguing and clinically meaningful pattern. When interventions included bisphosphonates, acupuncture-based combination therapies (e.g., CT_VD_BP_Acu_Pat) were associated with lower SUCRA values. In contrast, among interventions that did not include bisphosphonates, regimens such as CT_VD_Mox achieved notably higher SUCRA values. Within bisphosphonate-containing regimens, a reduction in ALP represents an expected pharmacodynamic outcome. Bisphosphonates exert potent inhibitory effects on bone turnover; by strongly suppressing osteoclastic bone resorption, they concurrently downregulate osteoblastic activity, resulting in decreased serum ALP levels (131). Accordingly, lower SUCRA values in this context indicate that these combined interventions effectively achieved the intended pharmacological endpoint. Conversely, in regimens without bisphosphonates, relative stability or elevation of ALP may reflect alternative therapeutic mechanisms. Interventions represented by CT_VD_Mox, which ranked highly according to SUCRA and were associated with increased post-treatment ALP levels, may exert mild stimulatory effects on bone formation through thermal-based therapies such as moxibustion. From the perspective of TCM, PMOP, also referred to as “bone atrophy (Gu Wei), “ is primarily attributed to impaired microcirculation and insufficient nourishment of the skeletal system. Moxibustion is believed to enhance systemic blood circulation, nourish bone tissue, and promote osteocyte growth (20). Supporting evidence from modern experimental studies has demonstrated that moxibustion can delay extracellular matrix degradation and chondrocyte apoptosis, increase medial osteophyte formation and subchondral bone plate thickness, and alleviate pathological changes associated with osteoarthritis (132), findings that are consistent with the results of the present NMA. Importantly, ALP is not exclusively derived from bone tissue but also originates from organs such as the liver. The near-infrared radiation generated by moxibustion may provide bioenergetic stimulation to the organism as a whole (133), which aligns with the holistic therapeutic concept emphasized in TCM. Consequently, higher SUCRA values may reflect the capacity of moxibustion-based therapies to mildly activate osteoblasts and potentially enhance systemic cellular activity, thereby facilitating bone repair and remodeling. Furthermore, moxibustion was a component of the most effective intervention for elevating OCN levels, providing NMA evidence to support its use as an adjunctive osteogenic therapy for osteoporosis.

LS and FN-BMD showed differential treatment responses, which were also influenced by BP use. The optimal physical stimulation (electroacupuncture vs. moxibustion) differed across skeletal sites, potentially reflecting site−specific differences in blood flow, mechanical loading, or neural regulation. In terms of improving LS-BMD, Cal_ElecAcu demonstrated particularly prominent efficacy. Calcitriol, the active metabolite of vitamin D, promotes intestinal, renal, and skeletal calcium–phosphate absorption and utilization via vitamin D receptor–mediated pathways, thereby exerting a pivotal influence on bone metabolism (134). Electroacupuncture, a distinctive therapeutic modality that integrates traditional acupuncture with pulsed electrical stimulation, has been shown to confer additional skeletal benefits. Experimental studies have reported that electroacupuncture at CV4 (Guanyuan) significantly ameliorates osteoporotic morphological changes in ovariectomized rats, increases serum ALP and bone Gla protein (BGP) levels, enhances fracture load, improves BMD, and activates the Wnt/β-catenin signaling pathway. It has therefore been proposed that electroacupuncture at CV4 promotes bone formation and bone metabolism in postmenopausal osteoporotic models, potentially through activation of the Wnt/β-catenin pathway (135). Taken together, this combined regimen may represent an effective alternative for patients who cannot tolerate, or are unwilling to use, more potent anti-osteoporotic agents such as bisphosphonates or teriparatide. Fire Dragon Moxibustion (FDMox) is an intensive form of moxibustion in which multiple lit moxa sticks are moved linearly along a meridian or muscle group in a “dragon−like” pattern, producing a broad thermal effect. Accordingly, FDMox delivers stronger thermal stimulation than conventional moxibustion, which may enhance microcirculation and bone metabolism in the femoral neck region. Notably, other moxibustion techniques with potent thermal effects—such as Thunder Fire Moxibustion (TFMox)—have been shown to improve lower limb balance function in female patients with PMOP and low muscle mass, an effect attributed to the alleviation of lower back pain and the enhancement of core lower−limb stabilizing muscle groups (136).

With respect to symptom relief, TCM_Acu and TCM_WarmAcu exhibited particularly favorable effects. Oral Chinese herbal medicine may intervene in the pathophysiological basis of PMOP at a systemic level through mechanisms such as phytoestrogen-like effects, regulation of bone turnover, and improvement of calcium and phosphorus metabolism. *In vitro* experiments have shown that *Herba Epimedium* enhances osteogenic differentiation of bone marrow mesenchymal stem cells (BMSCs) via the PI3K/Akt/mTOR pathway (137). Additionally, the traditional Chinese herbal formula Er-Zhi-Wan increases serum calcium and phosphorus levels in ovariectomized rats and exerts clear anti-osteoporotic effects (138). Acupuncture—particularly heat−generating techniques such as warm needling and fire needling—exerts anti−inflammatory and analgesic effects by increasing local blood flow and modulating inflammatory cytokines. Evidence suggests that acupuncture at specific acupoints, such as ST36 (Zusanli), exerts anti-inflammatory effects by modulating inflammation-related gene expression across multiple organ systems, including the humoral, digestive, and nervous systems. These effects are mediated through several pathways, including vagal nerve activity, the splenic nerve, the mitogen-activated protein kinase (MAPK) signaling pathway, and the TLR4/NF-κB pathway (139). From a modern biomedical perspective, these combined interventions may operate through multisystem and multitarget regulatory mechanisms, thereby comprehensively alleviating pain and systemic symptoms, which is highly consistent with the findings of the present NMA.

This study has several notable strengths. First, we systematically compared and quantitatively synthesized the multidimensional effects of more than 50 integrative Chinese and Western medicine strategies for the treatment of PMOP, encompassing a comprehensive evidence continuum ranging from patient-reported symptoms to biochemical and molecular biomarkers. In real-world clinical practice, PMOP management involves diverse pharmacological options alongside a broad spectrum of nonpharmacological TCM therapies, which enhances the clinical representativeness and external validity of our findings. Second, we provided a refined classification of external TCM therapies. Rather than treating “acupuncture” as a single, homogeneous intervention, we differentiated among conventional acupuncture, electroacupuncture, warm acupuncture, fire acupuncture, and multiple forms of moxibustion (e.g., Du meridian moxibustion, thunder-fire moxibustion, and fire-dragon moxibustion), as well as acupoint injection and acupoint application. This granular classification allows for more precise comparisons and offers clearer, practice-oriented guidance for selecting specific techniques. Third, by applying NMA, we were able to indirectly compare a large number of treatment regimens that have not been directly contrasted in conventional head-to-head RCTs, thereby constructing a comprehensive “efficacy landscape” of integrative therapies for PMOP.

However, several limitations of this study should be acknowledged. First, all 112 RCTs included in this NMA were conducted in China, and the study populations were almost exclusively Chinese. This geographical and ethnic homogeneity limits the generalizability of our findings to other populations, such as Africans. Furthermore, acupuncture and TCM enjoy deep sociocultural acceptance in China, which may enhance the placebo effect and treatment adherence compared to populations unfamiliar with these therapies. Consequently, the effect sizes observed in this analysis may not be directly transferable to settings where acupuncture has shallow cultural roots. Furthermore, some interventions combined acupuncture with Chinese herbal formulas, which are less standardized outside of China. Therefore, caution is warranted when extrapolating our results to other populations or healthcare systems. Future international, multicenter, RCTs involving diverse populations are needed to assess the external validity of acupuncture-based combination therapies for PMOP. Second, the follow-up periods in the studies included in this NMA were relatively short (10 days to 12 months), and the outcome measures analyzed were predominantly short- to mid-term indicators, such as TCM syndrome scores and VAS scores, whereas long-term clinically meaningful endpoints—most notably vertebral and non-vertebral fracture incidence—were lacking. Although one included trial reported hip fracture events, the overall evidence for fracture prevention remains extremely limited. As a result, the long-term benefits of combined acupuncture–pharmacotherapy strategies for PMOP remain uncertain. Third, the overall methodological quality of the included studies may have constrained the strength of the evidence. Limitations related to randomization procedures, allocation concealment, and blinding were frequently observed. For example, most included trials did not blind patients or outcome assessors for subjective outcomes (VAS scores, TCM syndrome scores, clinical efficacy). Lack of blinding is known to inflate effect sizes, particularly for patient reported outcomes. Therefore, the results for these subjective endpoints should be interpreted with caution. Although objective outcomes (BMD, bone turnover markers, estradiol) broadly supported the subjective findings, the potential for detection bias cannot be excluded. Future RCTs should incorporate blinding of outcome assessors and, where feasible, patients to minimize this bias. Fourth, statistically significant global inconsistency was observed for multiple outcome measures: LS-BMD, total TCM syndrome score, TCM syndrome low back pain score (including BP), PINP, E2, ALP (excluding BP), OCN (excluding BP), and OCN (including BP). The I² values for all these outcomes were low (<50%), ruling out statistical heterogeneity as the primary cause. The sources of inconsistency varied by outcome. For LS-BMD, total TCM syndrome scores, E2, and ALP (excluding BP), loop-based inconsistency tests identified closed loops with 95% CrIs that did not include 0, indicating significant loop inconsistency. The comparisons involved in these loops exhibited marked methodological heterogeneity (e.g., differences in electroacupuncture parameters, treatment duration, and combined interventions) and baseline differences (e.g., initial BMD or biomarker levels). Furthermore, insufficient standardization of TCM interventions—including variations in herbal formulas, acupoint selection, and operational parameters—may have significantly contributed to loop inconsistency, while also undermining the clinical reproducibility of the optimal regimens identified by this NMA. For the total TCM syndrome score, E2, ALP (excluding BP), and OCN (excluding BP), the presence of multi-arm studies within the network may have generated global inconsistencies; even in the absence of closed loops, multi-arm studies automatically create indirect comparisons, thereby introducing inconsistencies into the model. Data sparsity is another contributing factor. The network for the TCM syndrome-based low back pain score (including BP) included only 7 studies. For PINP, OCN (excluding BP), and OCN (including BP), most direct comparisons were supported by ≤2 studies, leading to unstable estimates and increasing the risk of global inconsistency. Given the sources of inconsistency described above, the SUCRA rankings and effect estimates for these affected outcomes should be considered exploratory findings rather than definitive conclusions until validated by higher-quality evidence. Future research should prioritize well-designed, large-scale RCTs with standardized intervention protocols and long-term follow-up to strengthen the evidence base and clarify the sustained clinical benefits of integrative therapies for PMOP.

## 5 Conclusion

There is no single “best” therapeutic regimen for the management of PMOP. Rather, optimized treatment combinations can be tailored to specific therapeutic goals—such as increasing BMD, alleviating pain, regulate bone metabolism markers—and the integration of pharmacotherapy with acupuncture-based interventions may yield synergistic benefits. Cal_ElecAcu was most effective for LS−BMD. For FN−BMD, CT_VD_TCM_FDMox and CT_VD_BP_TCM_Acu ranked best. For clinical symptoms, TCM_Acu led in clinical efficacy; TCM_Acu and CT_VD_BP_Acu_Mox improved low back pain; and CT_VD_BP_Acu_Pat reduced VAS scores. For bone metabolism, CT_VD_TCM_Acu (CTX reduction), CT_VD_BP_AcuInj (PINP reduction), and CT_VD_BP_TCM_ACE (E2 elevation) were most effective. CT_VD_Mox and CT_VD_BP_TCM_Acu_Mox showed the strongest OCN elevation. Overall, this NMA provides clinicians with an evidence-based framework to guide individualized treatment selection and supports a paradigm shift in PMOP management from a traditional “single-drug–oriented” strategy toward a personalized, patient-centered, integrative approach that combines conventional pharmacotherapy with TCM interventions. Nevertheless, as some comparisons were subject to moderate risk of bias and limited evidence, these findings should be interpreted cautiously and warrant confirmation in future large-scale, rigorously designed RCTs with long-term follow-up and direct head-to-head comparisons.

## Data Availability

The original contributions presented in the study are included in the article/**Supplementary Material**. Further inquiries can be directed to the corresponding author.

## Glossary

BMD: Bone mineral density
LS-BMD: Lumbar spine BMD
FN-BMD: Femoral neck BMD
PINP: Procollagen I N−terminal propeptide
PICP: C−terminal propeptide of type I procollagen
CTX/β−CTX: C−terminal telopeptide/β−isomerized C−terminal telopeptide
NTX: Type I collagen N−terminal peptide
OCN/BGP: Osteocalcin/bone glutamyl protein
N−MID: Molecular fragment of osteocalcin N−terminal
ALP: Alkaline phosphatase
BNALP/NBAP: Bone−specific alkaline phosphatase
TRAP−P/TRACP/TRAP−5b: Tartrate−resistant acid phosphatase (5b)
CICP: Collagen type I carboxyterminal elongation peptide
PYR: Pyridine (cross−links)
Hyp/Cr: Urine hydroxyproline/creatinine ratio
UCa/Cr: Urine calcium/creatinine ratio
E2: Estradiol
T: Testosterone
GH: Growth hormone
FSH: Follicle−stimulating hormone
LH: Luteinizing hormone
PGF2α: Prostaglandin F2α
PGE2: Prostaglandin E2
VIP: Vasoactive intestinal peptide
CGRP: Calcitonin gene−related peptide
NPY: Neuropeptide Y
SP: Substance P
OPG: Osteoprotegerin
OPN: Osteopontin
RANKL: Receptor activator of NF−κB ligand
IGF−1: Insulin−like growth factor−1
IGFBP−3: Insulin−like growth factor binding protein−3
TGF−β1: Transforming growth factor−β1
TNF−α: Tumor necrosis factor−α
VEGF: Vascular endothelial growth factor
AOPP: Advanced oxidation protein products
T−AOC: Total antioxidant capacity
SOD: Superoxide dismutase
MDA: Malondialdehyde
GSH: Glutathione
GSSG: Glutathione disulfide
VAS: Visual analogue scale
NRS: Numerical rating scale
ODI: Oswestry disability index
BPI: Brief pain inventory
PRI: Pain rating index
PPI: Present pain intensity
BBS: Berg balance scale
BMT: Biomechanical tension test
SF−36: 36−item short form health survey
PCS: Physical component summary
MCS: Mental component summary
OQOLS: Osteoporosis quality of life scale
COQOL: Chinese osteoporosis−targeted quality of life questionnaire
AE: Adverse event
ESR: Erythrocyte sedimentation rate
HCT: Hematocrit
Fib: Fibrinogen
PGC−1α: Peroxisome proliferator−activated receptor γ
SRC−3: Steroid receptor coactivator 3
ERRα: Estrogen−related receptor α
MAO−A: Monoamine oxidase A
MMSE: Mini−mental state examination
HAMA: Hamilton anxiety scale
PHQ−9: Patient health questionnaire−9
HMOFP: Hip and major osteoporotic fracture probability
PVP: Percutaneous vertebroplasty
BP: Bisphosphonate
CT: Calcitonin
VD: Vitamin D
Cal: Calcitriol
TCM: Traditional Chinese Medicine
Deno: Denosumab
Cel: Celecoxib capsules
Pla: Placebo
Acu: Standard acupuncture
ElecAcu: Electroacupuncture
WarmAcu: Warm acupuncture
FNeedle: Fire needle
TENeedle: Three−edged needle
MNKnife: Micro−needle knife
ShortPri: Short pricking
Mox: Moxibustion
GinMox: Ginger moxibustion
DuMox: Du−moxibustion (governor vessel)
FDMox: Fire dragon moxibustion
TFMox: Thunder−fire moxibustion
IMox: Indirect moxibustion
HSMox: Heat−sensitive moxibustion
AcuInj: Acupoint injection
Pat: Point Application Therapy
ACE: Acupoint catgut embedding
TCMStick: TCM sticking
Aps: Auricular point sticking
