# Supplementary material for: Evaluating combined acupuncture and antiresorptive therapy in Chinese women with postmenopausal osteoporosis: a systematic review and network meta-analysis
Source: Front Endocrinol (Lausanne). 2026 Jul 1;17:1784394. doi: 10.3389/fendo.2026.1784394 (PMC13368563; doi:10.3389/fendo.2026.1784394)
Supplement: Supplementary file 2 [file DataSheet2.docx]

# English-language databases

## Intervention-related terms

'**acupuncture therapy**' **OR** '**acupuncture**'

'acupuncture treatment' OR 'acupuncture treatments' OR 'pharmacoacupuncture treatment' OR 'pharmacoacupuncture therapy' OR 'acupotomy' OR 'acupotomies' OR 'shonishin' OR 'electroacupuncture' OR 'electro-acupuncture' OR 'auricular acupuncture' OR 'pharmacoacupuncture' OR 'acupressure' OR 'zhen jiu' OR 'traditional chinese medicine acupuncture' OR 'manual acupuncture' OR 'moxibustion' OR 'point embedding' OR 'transcutaneous electrical acupoint stimulation' OR 'auricular point' OR 'thumb-tack acupuncture' OR 'wrist-ankle acupuncture' OR 'warm acupuncture'

**Drugs：**

'Alendronate' OR 'Risedronate' OR 'Ibandronate' OR 'Zoledronic acid' OR 'Bisphosphonates'

'biphosphonate' OR 'biphosphonates' OR 'bisphosphonate' OR 'bisphosphonate derivative' OR 'bisphosphonates' OR 'diphosphonate derivative' OR 'diphosphonate series' OR 'diphosphonates' OR 'diphosphonic acid derivative' OR 'bisphosphonic acid derivative' OR 'alendronate' OR 'risedronate' OR 'ibandronate' OR 'zoledronic acid'

'Raloxifene' OR 'Bazedoxifene' OR 'Selective Estrogen Receptor Modulators'

'estrogen receptor modulators' OR 'oestrogen receptor modulators' OR 'selective estrogen receptor modulators' OR 'selective oestrogen receptor modulator' OR 'selective oestrogen receptor modulators' OR 'SERM' OR 'SERMs' OR 'selective estrogen receptor modulator' OR 'raloxifene' OR 'bazedoxifene'

calcitonin

'alpha calcitonin' OR 'alpha thyrocalcitonin' OR 'beta thyrocalcitonin' OR 'calcitar' OR 'calcitocin' OR 'calcitonia' OR 'calcitonin binding protein' OR 'calcitonin human' OR 'calcitonin m' OR 'calcitonin monomer' OR 'calcitonin release' OR 'calcitonin secretion' OR 'calcitonin (1-32)' OR 'calcitonine' OR 'cibacalcin' OR 'cibalcin' OR 'human calcitonin' OR 'porcine calcitonin' OR 'staporos' OR 'thyreocalcitonine' OR 'thyrocalcitonin' OR 'thyrocalcitonine' OR 'calcitonin' OR 'Calcitrin' OR 'Eel Calcitonin' OR 'Ciba 47175-BA' OR 'Callrate D600' OR 'Calcium Carbonate' OR 'Calcium'

'Teriparatide' OR 'Parathyroid Hormone'

'bovine parathyroid hormone' OR 'human parathyroid hormone' OR 'parathorm' OR 'parathormon' OR 'parathormone' OR 'parathyrin' OR 'parathyroid hormone 1-84' OR 'parathyroid hormones' OR 'PTH' OR 'parathyroid hormone' OR 'PTH (1-84)' OR 'Parathyroid Hormone Peptide (1-34)' OR 'PTH(1-34)' OR 'Parathyroid Hormone (1-34)' OR 'Natpara'

Denosumab

'Denosumab' OR 'AMG 162' OR 'Xgeva' OR 'Prolia'

romosozumab

'amg 785' OR 'amg785' OR 'amg-785' OR 'cdp 7851' OR 'cdp7851' OR 'cdp-7851' OR 'evenity' OR 'romosozumab aqqg' OR 'romosozumab-aqqg' OR 'sclerostin ab' OR 'romosozumab'

'Calcitriol' OR 'α-D3'

'Vitamin D' OR 'calcitriol' OR 'alfacalcidol'

'Chinese medicine' OR 'Medicine, Chinese Traditional'

'Xianlinggubao' OR 'Gushukang' OR 'Qianggu Capsule' OR 'Bushen Huoxue' OR 'Jianpi Bushen' OR 'bushen' OR 'Drugs' OR 'Western Medicine' OR ' Traditional Chinese Medicine' OR 'Combination of Traditional Chinese and Western Medicine' OR ' Chinese Medicine' OR 'Combination of Traditional Chinese and Western Medicine' OR ' Drug Therapy' OR 'Combination of Traditional Chinese and Western Medicine Therapy' OR 'Chinese herbal medicine' OR 'Chinese traditional medicine' OR 'medicine, Chinese traditional'

## Population

Osteoporosis, Postmenopausal

'post-menopausal bone loss' OR 'post-menopausal osteoporosis' OR 'post-menopause bone loss' OR 'post-menopause osteoporosis' OR 'type 1 osteoporosis' OR 'type i osteoporosis' OR 'Postmenopausal Osteoporoses' OR 'Perimenopausal Bone Loss' OR 'Postmenopausal Bone Losses' OR 'Perimenopausal Bone Losses' OR 'osteoporosis in postmenopausal women'

## Study design–related terms

'randomized controlled trial' OR 'random' OR 'placebo'

# Chinese-language databases

主题词：'针灸疗法'

'针'+'针灸疗法'+'针刺'+'电针'+'穴位埋线'+'温针灸'+'艾灸'+'穴位贴敷'+'耳针'+'针法'+'艾灸疗法'+'艾灸'+'大灸疗法'+'针灸'+'针灸治疗'+'温针'+'火针'+'毫针'+'体针'+'手针'+'灸法'+'隔物灸'+'雷火灸'+'麦粒灸'+'针刀'+'浮针'+'头针'

主题词：'骨质疏松, 绝经后'

'骨质疏松，绝经后'+'绝经期骨丢失'+'骨质丢失，绝经后'+'绝经后骨质疏松'+'更年期骨质疏松'+'围绝经期骨质疏松'+'闭经后骨质疏松'+'绝经后骨质丢失'+'绝经妇女骨质疏松症'+'I型骨质疏松'+'骨痿'+'骨枯'

主题词：'药物疗法'+'中西医结合疗法'+'二膦酸盐类'+'选择性雌激素受体调节剂'+'降钙素'+'甲状旁腺激素'+'狄诺塞麦'+'维生素D'+'骨化三醇'

'药物'+'西药'+'中药'+'中西药结合'+'中成药'+'联合用药'+'药物治疗'+'药物疗法'+'化学疗法'+'中西医结合疗法'+'TCM WM Therapy'+'双磷酸盐类'+'二膦酸盐类'+'双膦酸盐'+'利塞膦酸'+'唑来膦酸'+'帕米膦酸钠'+'伊班膦酸'+'氯膦酸'+'羟乙磷酸'+'99m锝美罗酸盐'+'阿屈膦酸盐'+'阿仑膦酸钠'+'选择性雌激素受体调节剂'+'SERMs'+'SERM'+'雷洛昔芬'+'雌激素'+'降钙素'+'甲状腺降钙素'+'降钙素原'+'降钙素-1'+'降钙素相关多肽α'+'降钙素病毒多蛋白前体'+'鳗鱼降钙素'+'苯乙哌啶酮47175-BA'+'苯乙哌啶酮47175BA'+'钙剂'+'钙尔奇D'+'碳酸钙'+'甲状旁腺激素'+'特立帕肽'+'PTH'+'甲状旁腺激素肽'+'甲状旁腺素'+'地舒单抗'+'狄诺塞麦'+'AMG162'+'保骼丽'+'癌骨瓦'+'维生素D'+'骨化三醇'+'阿法骨化醇'+'仙灵骨葆'+'骨疏康'+'强骨胶囊'+'补肾活血'+'健脾补肾'

主题词: '随机对照试验'

'随机'+'RCT'+'随机分组'+'对照'+'临床研究'+'安慰剂'
