# Supplementary material for: Evaluating combined acupuncture and antiresorptive therapy in Chinese women with postmenopausal osteoporosis: a systematic review and network meta-analysis
Source: Front Endocrinol (Lausanne). 2026 Jul 1;17:1784394. doi: 10.3389/fendo.2026.1784394 (PMC13368563; doi:10.3389/fendo.2026.1784394)
Supplement: Supplementary file 3 [file DataSheet3.docx]

| Study | Country | type | Patients | | treatment | | | duration | outcome | Jadad |
| --- | --- | --- | --- | --- | --- | --- | --- | --- | --- | --- |
|  |  |  | Sample size | Age | Intervention | Sample size | Age |  |  |  |
| Pan 2015 | china | RCT | 90 | ＜65 | CT_VD_BP; CT_VD_BP_Mox | 59;  27 | 54.30(5.70);  53.06(5.53) | 6 months | BMD/E2/ALP/TRAP-5b/OQOLS | 5 |
| Zou 2025 | china | RCT | 110 |  | CT_VD;  CT_VD_TCM_Mox | 55;  55 | 55.64(14.10)  54.30(5.00) | 3 months | clinical efficacy/TCM syndrome score/BMD/bone formation markers/hemorheological indexes/adverse reaction | 6 |
| Zheng 2021 | china | RCT | 80 | 45-65 | CT_VD_BP;  CT_VD_BP_Mox | 37;  38 | 55.92(3.93)  56.71(4.86) | 1 month | clinical efficacy/safety | 7 |
| Liang 2013 | china | RCT | 120 |  | CT_VD;  CT_VD_ACE | 60  60 | 66.90(5.80);  65.70(6.90) | 6 months | VAS score/BMD/quality of life score/clinical efficacy/TCM syndrome score/AE/E2 | 5 |
| Liu 2011 | china | RCT | 135 | 50-70 | CT_VD;  CT_VD_TCM  CT_VD_ACE | 45  45  45 | 59.8(8.6)  60.3(10.2)  62.5(9.7) | 6 months | VAS score/BMD/E2/quality of life score/TCM syndrome score/clinical efficacy/AE | 5 |
| Lai 2019 | china | RCT | 82 | ≥50 | CT_VD;  TCM_Acu | 41;  41 | 70.12(15.34);  70.09(15.28) | 18 days | clinical efficacy/TCM syndrome score/BMD/Serum Ca/Serum P/ALP/AE | 6 |
| Mao 2022 | china | RCT | 104 |  | CT_VD;  CT_VD_TCM_Acu | 52  52 | 66.4(6.7)  67.7(7.3) | 3 months | TCM syndrome score/bone pain score/ALP/N-terminal osteocalcin/osteocalcin/clinical efficacy | 6 |
| Li 2011 | china | RCT | 60 | 50-70 | CT_VD;  CT_VD_TCM_Acu_Mox | 29  31 | 61.00(5.50)  60.00(4.80) | 3 months | clinical efficacy/BMD | 5 |
| Zhang 2024 | china | RCT | 116 |  | CT_VD;  CT_VD_TCM_Acu | 58  58 | 57.56(4.32)  57.71(4.42) | 3 months | clinical efficacy/BMD/OQOLS | 6 |
| Zhou 2019 | china | RCT | 96 | 48-70 | CT_VD_GinMox;  CT_VD_TCM_GinMox | 48  48 | 59.08(3.78)  59.41(3.96) | 2 months | BMD/clinical efficacy | 6 |
| Ding 2022 | china | RCT | 112 | 60-85 | CT_VD;  CT_VD_TCM_Acu | 56  56 | 67.52(8.54)  66.91（7.96） | 3 months | TCM syndrome score/clinical efficacy/BMD/bone metabolic markers/BBS score | 6 |
| Huang 2016 | china | RCT | 130 | 60-88 | CT_VD;  CT_VD_Acu | 65  65 | 71.00(11.00)  72.00(10.00) | 3 months | VAS score/BMD/clinical efficacy | 5 |
| Yan 2021 | china | RCT | 66 | 60-79 | TCM_TENeedle;  CT_VD | 33  33 | 67.6;  65.5 | 6 weeks | rest pain score/walking pain score/clinical efficacy | 6 |
| Huang 2014 | china | RCT | 57 | 49-65 | Pat_FNeedle;  CT_VD | 29  28 | 58.62  57.98 | 1 month | VAS score/clinical efficacy | 7 |
| Wang 2023 | china | RCT | 112 | 50-70 | CT_Cal  CT_Cal_TCM_WarmAcu | 56  56 | 56.38(2.85)  56.49(2.73) | 3 months | clinical efficacy/VAS score/ODI/BMD/GH/IGF-1/PGF2α/SP | 6 |
| Wang 2023 | china | RCT | 80 | 50-70 | CT_Cal  CT_Cal_TCM_WarmAcu | 40  40 | 57.10(2.79)  56.86(2.85) | 3 months | clinical efficacy/BMD/VAS score/CTX/OPG/PGE2/NPY | 6 |
| Li 2016 | china | RCT | 92 | 43-70 | CT_VD_BP;  CT_VD_BP_DuMox | 46  46 | 56.73(4.72)  56.82(4.63) | 6 months | AST/ALT/SCR/serum Ca/OCN/BMD/clinical efficacy | 5 |
| Li 2017 | china | RCT | 90 | 45-75 | DuMox  TCM  TCM_DuMox | 30  30  30 | 63.6(8.7)  60.9(9.3)  61.7(7.9) | 1 month | VAS score/SF-36 | 5 |
| Shan 2023 | china | RCT | 72 | 45-65 | CT_VD_DuMox  CT_VD_Acu | 32  32 | 56.66(5.21)  56.16(5.01) | 3 months | TCM syndrome score/VAS score/BMD/clinical efficacy/AE | 6 |
| Lin 2013 | china | RCT | 70 | 45-75 | CT_VD  CT_VD_DuMox | 35  35 |  | 3 months | VAS score/ODI | 6 |
| Miao 2023 | china | RCT | 72 | 45-65 | CT_VD_DuMox  CT_VD_Acu | 36  36 | 54.94(5.25)  54.16(5.55) | 3 months | BMD/TCM syndrome score/VAS score/clinical efficacy/AE | 7 |
| Fan 2023 | china | RCT | 64 | 40-90 | CT_VD_BP;  CT_VD_BP_TCM_Acu | 30  30 | 60.5(9.89)  59.63(10.93) | 10 days | VAS score/ODI/TCM syndrome score/clinical efficacy/AE | 7 |
| Yuan 2017 | china | RCT | 143 | 55-85 | CT;  CT_TCM_ WarmAcu | 71;  72 | 62.90(5.79)  64.00(5.99) | 4 months | PINP/OCN/VEGF/TGF-β1/TNF-α/TCM syndrome score/clinical efficacy | 6 |
| Yuan 2017 | china | RCT | 60 | 55-90 | TCM_ WarmAcu;  BP | 30  30 | 72.53(16.15)  75.13(12.56) | 2 months | PINP/OCN/VEGF/TGF-β1/TNF-α/TCM syndrome score/VAS score/ODI/AE/clinical efficacy | 7 |
| Li 2024 | china | RCT | 70 | 50-75 | CT_VD_ShortPri;  CT_VD_Acu | 35  35 | 61.20(6.26)  60.51(5.60) | 3 months | VAS score/BMD/TCM syndrome score/clinical efficacy | 6 |
| Yuan 2001 | china | RCT | 90 |  | CT_VD  CT_VD_Aps | 45  45 |  | 3 months | BMD | 6 |
| Liu 2016 | china | RCT | 124 | 50-60 | CT_VD_BP;  CT_VD_BP_TCM_WarmAcu | 62  62 | 56.15(6.77)  55.86(6.92) | 6 months | TCM syndrome score/BMD/PGC-1α/SRC-3/ERRα/OPN/clinical efficacy | 6 |
| Liu 2011 | china | RCT | 105 | 53-70 | CT_VD  CT_VD_TCM  CT_VD_ACE | 35  35  35 | 62.8(5.9)  61.8(8.3)  63.7(3.8) | 6 months | VAS score/BMD/E2/clinical efficacy/AE | 6 |
| Yu 2015 | china | RCT | 40 | 45-70 | CT_VD_BP  CT_VD_BP_IMox | 20  20 | 62.01(7.02)  62.27(8.73) | 12 months | BMD/serum Ca/Serum P/ALP/clinical efficacy | 5 |
| Zhang 2022 | china | RCT | 100 | 45-65 | CT  CT_TCM_IMox | 50  50 | 54  55 | 10 weeks | clinical efficacy/VAS score/BMD | 5 |
| Shen 2012 | china | RCT | 60 | 55-70 | TCM  TCM_Acu | 30  30 |  | 3 months | clinical efficacy/TCM syndrome score/Back pain score/BMD/AE | 6 |
| Xiong 2024 | china | RCT | 66 | 60-76 | CT_VD  CT_VD_TCM_FDMox | 33  33 | 68.15(2.14)  68.24(2.20) | 1 month | TCM syndrome score/BMD | 6 |
| Sun 2017 | china | RCT | 60 | 45-70 | CT_VD  CT_VD_Acu | 25  23 | 59.88(6.18)  59.88(6.18) | 3 months | PINP/CTX/OPG/BMD/VAS score/SF-36/TCM syndrome score | 6 |
| Ren 2022 | china | RCT | 72 | 45-70 | CT_VD_BP  CT_VD_BP_Acu | 36  34 | 64.97(1.50)  65.19(1.05) | 6 months | BMD/TCM syndrome score/AE/E2/CTX/TRACP/OCN/ALP/AOPP/TAOC/SOD/MAOA | 7 |
| Chen 2021 | china | RCT | 70 | 45-70 | CT_VD;  CT_VD_Acu | 31  32 | 63.56(4.86)  63.61(4.60) | 3 months | clinical efficacy/Serum GH/Serum IGF-1/E2/FSH/LH/PINP/CTX/BMD/TCM syndrome score/Quality of life score/AE | 7 |
| Hu 2023 | china | RCT | 120 | 53-77 | TCM  TCM_Acu | 60  60 | 65.29(3.24)  66.55(3.82) | 6 months | TCM syndrome score/OCN/BMD/PTH/ALP/VAS score | 6 |
| You 2020 | china | RCT | 66 | 50-70 | CT_VD  CT_VD_TFMox | 33  33 | 62.23(5.17)  60.22(5.81) | 3 months | AE/PINP/CTX/BMD/VAS score/clinical efficacy/TCM syndrome score | 7 |
| Yang 2022 | china | RCT | 80 |  | CT_VD  CT_VD_TFMox | 40  40 | 66.28(6.02)  66.72(5.93) | 1 month | PRI/PPI/VAS score/CTX/OCN/CICP/BMD | 6 |
| Chen 2022 | china | RCT | 70 | 52-69 | CT_VD;  CT_VD_Acu | 32  31 | 64(5)  64(5) | 3 months | BMD/GH/IGF-1/TCM syndrome score/SF-36/clinical efficacy/AE | 7 |
| Lu 2014 | china | RCT | 47 |  | CT_VD  CT_VD_ACE | 22  25 | 62.14(6.34)  60.84(6.96) | 6 months | pain score/clinical efficacy/BMD | 6 |
| Liu 2020 | china | RCT | 60 | 45-70 | CT_VD_BP_Pla;  CT_VD_BP_Pat | 30  30 | 61.47(5.18)  61.53(3.95) | 6 months | BMD/MMSE/VAS score/osteoporosis symptom score/TCM symptom efficacy/AE | 7 |
| Chen 2021 | china | RCT | 126 | 50-68 | CT  CT_Acu | 63  63 | 56.50(3.20)  55.40(3.10) | 1.5 months | VAS score/HAMA/TCM syndrome score/clinical efficacy/E2/OCN/CTX/AE | 6 |
| Hui 2022 | china | RCT | 72 | 53-65 | CT_VD_ElecAcu;  CT_VD _TCM_ElecAcu | 33  34 | 58.00(5.31);  56.00(4.52) | 3 months | clinical efficacy/TCM syndrome score/BMD/AE | 7 |
| Wu 2018 | china | RCT | 56 | 45-65 | CT_VD  TCM_Mox | 28  28 | 59.25(2.91)  58.04(3.20) | 4 months | VAS score/BMD/E2 | 6 |
| Wu 2024 | china | RCT | 82 | 55-61 | Cal  Cal_TCM_ACE | 41  41 | 58.11(2.55)  58.13(2.56) | 3 months | clinical efficacy/clinical symptom score/BMD/ALP/TRACP-5b/E2/FSH/LH | 6 |
| Wang 2022 | china | RCT | 80 | 48-65 | CT;  CT_TCM_Acu | 40  40 | 58.43(1.03)  58.40(1.06) | 3 months | TCM syndrome score/PINP/CTX/BMD | 3 |
| Pan 2022 | china | RCT | 62 | 45-65 | CT_VD  CT_VD_MNKnife_Mox | 31  31 | 45-65  45-65 | 1 month | clinical efficacy/BMD/VAS score/clinical symptoms score/COQOL | 6 |
| Ouyang 2012 | china | RCT | 90 | 62.45(7.68) | CT_VD  CT_VD_TCM  CT_VD_Mox | 28  30  29 | 62.29(7.18)  63.87(7.71)  61.14(7.68) | 3 months | syndrome score of kidney-yang deficiency/ VAS score/infrared thermogram temperature value/TCM syndrome score/clinical efficacy/BMD/OCN/ALP/TRACP-5b/OPG/Serum Ca/Serum P | 5 |
| Chen 2004 | china | RCT | 60 |  | CT_VD  CT_VD_WarmAcu | 30  30 | 57.63(7.68)  58.12(7.25) | 6 months | clinical efficacy/BMD/E2/T | 7 |
| Luo 2019 | china | RCT | 80 | 50-70 | TCM  TCM_WarmAcu | 40  40 | 59.27(5.08)  58.30(5.27) | 3 months | clinical efficacy/VAS score/E2/FSH/LH/MDA/SOD/GSH/GSSG/AE | 6 |
| Zhu 2022 | china | RCT | 80 | ≥45 | WarmAcu  TCM_WarmAcu | 40  40 | 56.93(5.54)  57.61(5.67) | 1 month | clinical efficacy/TCM syndrome score/ALP/OCN/AE | 6 |
| Cai 2015 | china | RCT | 85 | 46-60 | CT_VD  CT_VD_WarmAcu | 42  43 | 50(6)  51(7) | 12 months | BMD/OCN/Hyp/Cr | 6 |
| Zhang 2023 | china | RCT | 60 | 45-75 | CT_VD  CT_VD_WarmAcu | 30  30 | 60.32(1.63)  60.54(1.52) | 2 months | clinical efficacy/VAS score/BMD/TCM syndrome score/E2/FSH/LH/TNF-α/IL-6/IGF-1 | 6 |
| Lin 2020 | china | RCT | 90 | 50-70 | CT_VD  CT_VD_DuMox  CT_VD_ChenpiDuMox | 30  30  30 | 60.75(5.09)  59.50(5.92)  60.85(7.93) | 3 months | VAS score/SF-36 | 6 |
| Zhao 2015 | china | RCT | 100 | 55-65 | CT_VD_BP  CT_VD_BP_Acu_Cup | 50  50 | 59.10(2.03)  61.50(2.27) | 1 month | VAS score/BPI score/BMT | 6 |
| Gu 2018 | china | RCT | 60 | 68-52 | BP  BP_ACE | 30  30 | 62(5)  62(5) | 6 months | Syndrome integral of postmenopausal osteoporosis/BMD/E2/clinical efficacy | 7 |
| Chen 2010 | china | RCT | 120 | 50-80 | ElecAcu _Mox  CT_ElecAcu _Mox | 60  60 | 71  72 | 20 days | clinical efficacy | 6 |
| Chen 2011 | china | RCT | 105 | 50-70 | CT_VD  CT_VD_TCM  CT_VD_ACE | 35  35  35 | 62.2(2.8) | 6 months | TCM syndrome score/IOF Quality of Life score/AE | 6 |
| Li 2024 | china | RCT | 72 | 45-85 | Cal  Cal_TCM_ACE | 36  36 | 64.53(7.58)  64.22(9.10) | 2 months | TCM syndrome score/clinical efficacy/VAS score/CTX/BMD/AE | 3 |
| Shi 2021 | china | RCT | 90 | 50-65 | CT_VD  CT_VD_ACE | 45  45 | 57.45(3.94)  58.03(2.80) | 6 months | BMD/OPG/RANKL/E2/ALT/TG/TC/Cr/BUN/clinical efficacy/VAS score/Spleen and stomach function assessment /OP syndrome score | 7 |
| Wang 2016 | china | RCT | 120 | ≥50 | ACE  TCM  TCM_ACE  E | 30  30  30  30 | 62.43(8.13)  61.18(8.86)  62.21(7.99)  60.06(9.02) | 3 months | OCN/CT/ALP/E2/BMD/VAS score/clinical efficacy | 6 |
| Li 2022 | china | RCT | 104 | 50-70 | CT_VD  CT_VD_Pat | 52  52 | 56.63(3.19)  57.92(3.46) | 6 months | BMD/TCM syndrome score/SF-36/ODI score | 6 |
| Kang 2022 | china | RCT | 90 | 45-70 | CT_VD_BP  CT_VD_BP_Pat | 45  45 | 62.02(3.79)  61.27(4.07) | 3 months | VAS score/TCM syndrome score/clinical efficacy/ODI score/BMD/AE | 7 |
| Ma 2017 | china | RCT | 80 | 75.38(2.38) | CT_VD_BP  CT_VD_BP_Pat | 40  40 | 74.97(2.38）  75.81(2.34) | 12 months | OPG/RANKL/BMD/fracture | 6 |
| Chen 2024 | china | RCT | 110 | 55-85 | CT_VD_Deno;  CT_VD_Deno_Pat | 55  55 | 58.1(4.0)  59.7(6.1) | 6 months | BMD/PINP/CTX/VAS score/TCM syndrome score | 6 |
| Zou 2019 | china | RCT | 136 | ＞60 | CT_VD_BP  CT_VD_BP_AcuInj | 68  68 | 71(3)  70(4) | 6 months | clinical efficacy/TCM syndrome score/PINP/CTX/NBAP/BMD | 6 |
| Chen 2025 | china | RCT | 96 | 40-75 | CT_VD_Cel_Acu_Mox;  VD_Cel_Acu_Mox | 47  48 | 50.09(15.36)  46.04(13.75) | 1 month | clinical efficacy/ODI score/BMD/Loss of height of anterior edge of injured vertebra/Cobb angle/AE | 6 |
| Zhang 2025 | china | RCT | 120 | 55-70 | CT_VD_BP  CT_VD_BP_Pat | 60  60 | 58.19(3.30)  58.54(3.34) | 12 months | BMD/E2/FSH/PINP/CTX/VAS score/SF-36 | 5 |
| Wang 2015 | china | RCT | 60 | 42-65 | TCM_Mox;  Cal | 30  30 | 56.7  54.9 | 4 months | BMD/clinical efficacy | 4 |
| Guo 2021 | china | RCT | 72 | 50-75 | CT_VD_BP  CT_VD_BP_Acu | 36  36 | 63.56(3.82)  64.06(4.42) | 13 weeks | BMD/CTX/TCM syndrome score/SF-36/clinical efficacy/AE | 6 |
| Feng 2021 | china | RCT | 72 | 50-75 | CT_VD_BP  CT_VD_BP_Acu | 36  36 | 63.91(5.58)  63.57(5.63) | 13 weeks | BMD/ALP/SF-36/TCM syndrome score/clinical efficacy | 5 |
| Wang 2025 | china | RCT | 100 | 50-70 | CT_VD_BP  CT_VD_BP_Acu | 50  50 | 58.97(1.59)  58.92(1.52) | 1 month | clinical efficacy/VAS score/TCM syndrome score/ODI score/Serum Ca/Serum P/N-MID/ALP/CTX/BMD/AE | 6 |
| Huo 2020 | china | RCT | 360 | 55-73 | CT_VD_BP  CT_VD_BP_Acu | 180  180 | 61.29(7.16)  60.63(6.51) | 1 month | BMD/Serum Ca/Serum P/ALP/SF-36/clinical efficacy | 5 |
| Liu 2018 | china | RCT | 88 | 47-69 | CT_VD  CT_VD_Acu_GinMox | 44  44 | 57.1;  56.8 | 3 months | BMD/SF-36 | 6 |
| Chen 2018 | china | RCT | 63 |  | CT_VD_PVP  CT_VD_PVP_Acu | 31  32 | 77.02(5.21)  76.55(6.78) | 6 months | ODI score/VAS score/BMD | 6 |
| Wang 2008 | china | RCT | 57 | 40-75 | CT_VD_BP  CT_VD_BP_Acu | 25  32 | 59.2  58.5 | 6 months | BMD | 5 |
| Yu 2021 | china | RCT | 216 | 50-69 | CT_VD_BP_Acu;  CT_VD_BP_TFMox | 108  108 | 60(4)  59(4) | 3 months | ALP/E2/OCN/CTX/BMD/TCM syndrome score/VAS score/AE/clinical efficacy | 6 |
| Wang 2023 | china | RCT | 90 | 50-80 | CT_VD_BP;  CT_VD_BP_Acu | 45  45 | 62.20(8.24)  62.17(8.22) | 3 months | TCM syndrome score/BMD/VAS score | 6 |
| Li 2023 | china | RCT | 86 | 52-64 | CT_VD_BP  CT_VD_BP_Acu_Pat | 43  43 | 57.06(5.57)  58.03(6.03) | 3 months | clinical efficacy/BMD/Serum Ca/Serum P/ALP/PICP/OCN/CTX/VAS score/AE | 5 |
| Qu 2021 | china | RCT | 60 | 40-75 | CT_VD_BP  CT_VD_BP_Acu | 30  30 | 58.11(4.96)  58.32(4.39) | 3 months | BMD/TCM syndrome score/E2/IL-17/SF-36/clinical efficacy | 7 |
| Liu 2023 | china | RCT | 66 | 50-70 | CT_VD  CT_VD_Acu | 29  31 | 59.45(5.34)  60.03(5.06) | 3 months | BMD/TCM syndrome score/Body composition analysis/clinical efficacy | 5 |
| Liu 2023 | china | RCT | 66 | 50-70 | CT_VD_BP  CT_VD_BP_Acu | 30  30 | 62.89(7.68)  59.37(8.36) | 6 months | VAS score/TCM syndrome score/BMD/PINP/CTX/E2/serum ferritin/MDA/SOD/clinical efficacy/AE | 7 |
| Li 2012 | china | RCT | 56 | 52-68 | CT_VD_BP_E2;  CT_VD_BP_E2_TCM_Acu | 29  27 | 55.34  56.68 | 2 months | clinical efficacy | 6 |
| Ye 2023 | china | RCT | 80 | 55-79 | TCM;  TCM_Acu | 40  40 | 68(5)  68(5) | 2 months | BMD/BAP/OCN/NTX/CD4+T/CD8+T/CD4+T:CD8+T/TCM syndrome score/AE | 6 |
| Wang 2011 | china | RCT | 86 | 48-76 | CT_VD  CT_VD_TCM_Acu | 44  42 | 54.52  56.68 | 6 months | clinical efficacy | 5 |
| Zhou 2024 | china | RCT | 63 |  | CT_VD_TCM;  CT_VD_TCM_Acu | 21  42 | 62,38(5.09)  62.12(5.03) | 3 months | NRS score/BMD/25-OH-D/Serum Ca/Serum P/OPG/RANKL/OPG:RANKL/AE | 6 |
| Zhou 2012 | china | RCT | 100 | ≤65 | Cal  Cal_ElecAcu | 50  50 | 58(5)  56(7) | 6 months | clinical efficacy/pain score/BMD/Serum Ca/ Serum P/ALP/24 h Ca/Cr/E2/AE | 5 |
| Lin 2006 | china | RCT | 40 | 45-65 | Acu  TCM_Acu | 20  20 | 57.61(5.41)  57.80(5.20) | 2 months | bone pain score /kupper score/E2/OCN/IL-6 | 5 |
| Huang 2024 | china | RCT | 65 | 55-64 | VD_BP  VD_BP_Acu | 33  32 | 59.81(2.15)  59.58(2.65) | 3 months | BMD/VAS score/clinical efficacy | 5 |
| Zhuo 2000 | china | RCT | 62 | 50-70 | CT_VD_Acu  CT_VD | 32  30 | 63(4.5)  62(4.3) | 6 months | BMD | 5 |
| Zhou 2011 | china | RCT | 100 | ≤65 | ElecAcu  Cal_ElecAcu | 50  50 | 25.16(2.07)  26.02(1.72) | 5 months | pain score/BMD/Serum Ca/Serum P/ALP/24h urinary Ca/Cr/E2/clinical efficacy/AE | 5 |
| Han 2005 | china | RCT | 90 | 45-68 | Acu  TCM  TCM_Acu | 30  30  30 | 60.5 | 1 month | Bone pain symptoms efficacy/clinical efficacy/Serum Ca/Serum P/ALP/E2/progesterone | 5 |
| Wang 2013 | china | RCT | 150 | ≤65 | ElecAcu;  Cal  Cal_ElecAcu | 50  50  50 |  | 6 months | VAS score/BMD/Serum Ca/Serum P/ALP/urinary Ca/Cr/E2/clinical efficacy | 5 |
| Wu 2017 | china | RCT | 60 | 45-65 | CT_VD  CT_VD_Mox | 30  30 |  | 3 months | BMD/Clinical Bone Pain Score/TCM syndrome score | 5 |
| An 2022 | china | RCT | 70 |  | Cel;  TCM_Acu | 32  32 | 75.03(7.18);  74.78(7.12) | 2 weeks | VAS score/ODI score/TCM syndrome score | 6 |
| Wu 2023 | china | RCT | 76 | 45-75 | CT_VD_BP;  CT_VD_Acu | 38  38 | 63.92(5.53)  62.69(5.39) | 3 months | VAS score/TCM syndrome score/PINP/CTX/BMD/SF/E2/MDA/SOD/clinical efficacy/AE | 6 |
| Luo 2025 | china | RCT | 82 | 45-75 | CT_VD_BP;  CT_VD_Acu | 41  41 | 60.02(12.95)  61.44(9.37) | 3 months | BMD/HMOFP/AE | 6 |
| Lin 2016 | china | RCT | 40 | 46-62 | CT_VD_BP  CT_VD_BP_Acu | 20  20 | 53.62(2.37) | 6 months | BMD/symptom score/CTX/OPG/Serum Ca/Serum P | 6 |
| Wang 2016 | china | RCT | 90 | 45-70 | CT_VD;  TCM;  TCM_FNeedle | 30  30  30 | 57.98(13.64)  59.42(12.19)  58.65(12.37) | 3 months | VAS score/BMD/IL-6/OPG/clinical efficacy | 5 |
| He 2015 | china | RCT | 100 | 55-65 | CT_VD;  CT_VD_Pat | 100  100 |  | 6 months | BMD/PINP/CTX/VAS score | 5 |
| Zhang 2014 | china | RCT | 80 | 62.3(9.6) | TCM  TCM_Acu | 40  40 | 63.1(7.7)  62.2(8.2) | 3 months | clinical efficacy | 5 |
| Cao 2021 | china | RCT | 60 | 55-65 | CT_VD_BP;  CT_VD_BP_TCM_ACE | 30  30 | 59.25(4.20)  59.81(4.80) | 3 months | clinical efficacy/TCM syndrome score/ALP/PINP/Uca/Cr/E2/FSH/LH/BMD | 6 |
| Le 2015 | china | RCT | 160 | 45-65 | Cal  TCM_Aps | 80  80 |  | 6 months | clinical efficacy | 6 |
| Ma 2024 | china | RCT | 90 | ＞50 | CT_VD_TCM  CT_VD_Acu  CT_VD_TCM_Acu | 30  30  30 | 67.07(7.38)  66.47(8.63)  67.90(6.90) | 3 months | VAS score/TCM syndrome score/BMD/CTX | 6 |
| Hao 2013 | china | RCT | 83 | 53-69 | CT_BP  CT_BP_TCM_Acu | 41  42 |  | 2 months | clinical efficacy/BMD | 5 |
| Wu 2026 | china | RCT | 68 | 45-80 | CT_VD_BP  CT_VD_BP_Mox | 34  34 | 64.24(8.58)  64.24(8.45) | 3 months | TCM syndrome score/clinical efficacy/CTX/PINP/OCN/25OHD/PTH/ | 4 |
| Liu 2026 | china | RCT | 120 | 45-65 | CT_VD_BP_Acu  CT_VD_BP_Acu_Mox | 60  60 | 56.2(8.2)  56.2(8.2) | 3 months | SF-36/VAS score/ODI/PINP/BMP-2/OCN/CTX/TCM syndrome score/clinical efficacy/AE | 4 |
| Cai 2025 | china | RCT | 80 | 48-66 | CT_VD  CT_VD_Acu | 40  40 | 58.26(3.62)  59.10(3.44) | 6 weeks | clinical efficacy/BMD/PINP/TRAP-5b/CTX/VAS score/PHQ-9/VIP/CGRP/SP/NPY | 4 |
| Luo 2025 | china | RCT | 64 | 70-80 | CT_VD_BP  CT_VD_BP_Acu_DuMox | 32  31 |  | 3 months | TCM syndrome score/VAS score/PINP/CTX | 4 |
| Li 2026 | china | RCT | 100 | 55-75 | TCM  TCM_WarmAcu | 50  50 | 66.25(8.54)  65.95(8.96) | 3 months | clinical efficacy/VAS score/ODI/vertebral body front edge height/Cobb angle/BMD/PTH/OCN | 5 |
| Wang 2026 | china | RCT | 84 | 55-80 | CT_VD_BP_Acu  CT_VD_BP_TCM_Acu | 42  42 | 68.05(10.33)  67.81(10.26) | 6 months | TCM syndrome score/VAS score/BMD/IGFBP-3/N-MID/CTX/clinical efficacy | 4 |
| Zhang 2026 | china | RCT | 120 | 55-75 | CT_VD_Deno  CT_VD_Deno_WarmAcu | 60  60 | 66.3(4.9)  65.8(5.2) | 6 months | BMD/VAS score/CTX/PINP/SF-36/AE | 6 |

# Abbreviations

## 1. Outcomes

**Bone density：**

BMD: Bone mineral density

LS-BMD: Lumbar spine BMD

FN-BMD: Femoral neck BMD

**Bone turnover markers：**

PINP: Procollagen I N‑terminal propeptide

PICP: C‑terminal propeptide of type I procollagen

CTX / β‑CTX: C‑terminal telopeptide / β‑isomerized C‑terminal telopeptide

NTX: Type I collagen N‑terminal peptide

OCN / BGP: Osteocalcin / bone glutamyl protein

N‑MID: Molecular fragment of osteocalcin N‑terminal

ALP: Alkaline phosphatase

BNALP / NBAP: Bone‑specific alkaline phosphatase

TRAP‑P / TRACP / TRAP‑5b: Tartrate‑resistant acid phosphatase (5b)

CICP: Collagen type I carboxyterminal elongation peptide

PYR: Pyridine (cross‑links)

Hyp/Cr: Urine hydroxyproline/creatinine ratio

UCa/Cr: Urine calcium/creatinine ratio

**Hormones：**

E2: Estradiol

T: Testosterone

GH: Growth hormone

FSH: Follicle‑stimulating hormone

LH: Luteinizing hormone

PGF2α: Prostaglandin F2α

PGE2: Prostaglandin E2

VIP: Vasoactive intestinal peptide

CGRP: Calcitonin gene‑related peptide

NPY: Neuropeptide Y

SP: Substance P

**Cytokines and growth factors**

OPG: Osteoprotegerin

OPN: Osteopontin

RANKL: Receptor activator of NF‑κB ligand

IGF‑1: Insulin‑like growth factor‑1

IGFBP‑3: Insulin‑like growth factor binding protein‑3

TGF‑β1: Transforming growth factor‑β1

TNF‑α: Tumor necrosis factor‑α

VEGF: Vascular endothelial growth factor

**Oxidative stress**

AOPP: Advanced oxidation protein products

T‑AOC: Total antioxidant capacity

SOD: Superoxide dismutase

MDA: Malondialdehyde

GSH: Glutathione

GSSG: Glutathione disulfide

**Pain and function scores**

VAS: Visual analogue scale

NRS: Numerical rating scale

ODI: Oswestry disability index

BPI: Brief pain inventory

PRI: Pain rating index

PPI: Present pain intensity

BBS: Berg balance scale

BMT: Biomechanical tension test

**Quality of life:**

SF‑36: 36‑item short form health survey

PCS: Physical component summary

MCS: Mental component summary

OQOLS: Osteoporosis quality of life scale

COQOL: Chinese osteoporosis‑targeted quality of life questionnaire

**Others:**

AE: Adverse event

ESR: Erythrocyte sedimentation rate

HCT: Hematocrit

Fib: Fibrinogen

PGC‑1α: Peroxisome proliferator‑activated receptor γ coactivator‑1α

SRC‑3: Steroid receptor coactivator 3

ERRα: Estrogen‑related receptor α

MAO‑A: Monoamine oxidase A

MMSE: Mini‑mental state examination

HAMA: Hamilton anxiety scale

PHQ‑9: Patient health questionnaire‑9

HMOFP: Hip and major osteoporotic fracture probability

PVP: Percutaneous vertebroplasty

## 2. Drug classes

BP: Bisphosphonate

CT: Calcitonin

VD: Vitamin D

Cal: Calcitriol

TCM: Traditional Chinese Medicine

E: Estrogen

Deno: Denosumab

Cel: Celecoxib capsules

Pla: Placebo

## 3. Acupuncture and therapeutic modalities

**Needling:**

Acu: Standard acupuncture

ElecAcu: Electroacupuncture

WarmAcu: Warm acupuncture

FNeedle: Fire needle

TENeedle: Three‑edged needle

MNKnife: Micro‑needle knife

ShortPri: Short pricking

**Moxibustion:**

Mox: Moxibustion

GinMox: Ginger moxibustion

DuMox: Du‑moxibustion (governor vessel)

FDMox: Fire dragon moxibustion

TFMox: Thunder‑fire moxibustion

IMox: Indirect moxibustion

HSMox: Heat‑sensitive moxibustion

**Other techniques:**

AcuInj: Acupoint injection

Pat: Point Application Therapy

ACE: Acupoint catgut embedding

TCMStick: TCM sticking

Aps: Auricular point sticking
