# Supplementary material for: Evaluating combined acupuncture and antiresorptive therapy in Chinese women with postmenopausal osteoporosis: a systematic review and network meta-analysis
Source: Front Endocrinol (Lausanne). 2026 Jul 1;17:1784394. doi: 10.3389/fendo.2026.1784394 (PMC13368563; doi:10.3389/fendo.2026.1784394)
Supplement: Supplementary file 4 [file DataSheet4.docx]

# LS-BMD

## Consistency

Results on the Mean Difference scale

Iterations = 20001:70000

Thinning interval = 1

Number of chains = 4

Sample size per chain = 50000

1. Empirical mean and standard deviation for each variable,

plus standard error of the mean:

Mean SD Naive SE Time-series SE

d.Cal.Cal_ElecAcu 0.1899430 0.09676 0.0002164 0.0002529

d.Cal.ElecAcu -0.0100720 0.12771 0.0002856 0.0003318

d.CT_VD.CT_VD_ACE 0.0717219 0.06071 0.0001358 0.0001627

d.CT_VD.CT_VD_Acu 0.0098693 0.06192 0.0001385 0.0001526

d.CT_VD.CT_VD_Acu_GinMox 0.0502759 0.13900 0.0003108 0.0003378

d.CT_VD.CT_VD_MNKnife_Mox 0.0100338 0.13694 0.0003062 0.0003138

d.CT_VD.CT_VD_Mox -0.0005021 0.13608 0.0003043 0.0003034

d.CT_VD.CT_VD_TCM 0.0847363 0.08205 0.0001835 0.0003065

d.CT_VD.CT_VD_TCM_Acu 0.2634542 0.08893 0.0001988 0.0003260

d.CT_VD.CT_VD_TCM_FDMox 0.0999876 0.13774 0.0003080 0.0003220

d.CT_VD.CT_VD_TCM_Mox 0.0400822 0.13657 0.0003054 0.0003113

d.CT_VD.CT_VD_TFMox 0.0199398 0.14393 0.0003218 0.0003885

d.CT_VD.TCM_Mox 0.0498777 0.13644 0.0003051 0.0003133

d.CT_VD_Acu.CT_VD_BP -0.0195755 0.13668 0.0003056 0.0003247

d.CT_VD_Acu.CT_VD_ShortPri -0.0001222 0.13724 0.0003069 0.0003136

d.CT_VD_BP.CT_VD_BP_Acu 0.0458542 0.06124 0.0001369 0.0001403

d.CT_VD_BP.CT_VD_BP_Mox 0.0195904 0.13647 0.0003052 0.0003109

d.CT_VD_BP.CT_VD_BP_TCM_WarmAcu 0.0295088 0.13682 0.0003059 0.0003159

d.CT_VD_BP_Acu.CT_VD_BP_TCM_Acu 0.0798500 0.13703 0.0003064 0.0003178

d.TCM_Mox.Cal -0.0302809 0.14209 0.0003177 0.0003779

sd.d 0.1326340 0.02889 0.0000646 0.0001870

2. Quantiles for each variable:

2.5% 25% 50% 75% 97.5%

d.Cal.Cal_ElecAcu -0.003057 0.128266 0.1900486 0.25160 0.3820

d.Cal.ElecAcu -0.263759 -0.092055 -0.0105342 0.07133 0.2449

d.CT_VD.CT_VD_ACE -0.047680 0.032786 0.0712710 0.11039 0.1938

d.CT_VD.CT_VD_Acu -0.113381 -0.029669 0.0099619 0.04949 0.1327

d.CT_VD.CT_VD_Acu_GinMox -0.225574 -0.038942 0.0503373 0.13931 0.3262

d.CT_VD.CT_VD_MNKnife_Mox -0.260842 -0.077577 0.0099306 0.09763 0.2823

d.CT_VD.CT_VD_Mox -0.272237 -0.087085 -0.0004115 0.08617 0.2705

d.CT_VD.CT_VD_TCM -0.073941 0.031271 0.0833811 0.13671 0.2514

d.CT_VD.CT_VD_TCM_Acu 0.086251 0.206353 0.2639400 0.32075 0.4393

d.CT_VD.CT_VD_TCM_FDMox -0.173171 0.011626 0.0999784 0.18854 0.3721

d.CT_VD.CT_VD_TCM_Mox -0.231001 -0.047382 0.0400899 0.12738 0.3118

d.CT_VD.CT_VD_TFMox -0.265908 -0.073038 0.0201508 0.11320 0.3057

d.CT_VD.TCM_Mox -0.222168 -0.036770 0.0498218 0.13664 0.3212

d.CT_VD_Acu.CT_VD_BP -0.291836 -0.106748 -0.0193809 0.06759 0.2528

d.CT_VD_Acu.CT_VD_ShortPri -0.274093 -0.088081 -0.0002933 0.08732 0.2723

d.CT_VD_BP.CT_VD_BP_Acu -0.076391 0.006905 0.0458489 0.08521 0.1669

d.CT_VD_BP.CT_VD_BP_Mox -0.252571 -0.067585 0.0194126 0.10623 0.2917

d.CT_VD_BP.CT_VD_BP_TCM_WarmAcu -0.243034 -0.057612 0.0296112 0.11699 0.3009

d.CT_VD_BP_Acu.CT_VD_BP_TCM_Acu -0.192852 -0.007628 0.0801100 0.16732 0.3523

d.TCM_Mox.Cal -0.312262 -0.121499 -0.0303070 0.06107 0.2516

sd.d 0.088289 0.112158 0.1285222 0.14853 0.2004

-- Model fit (residual deviance):

Dbar pD DIC

70.26891 68.37006 138.63897

69 data points, ratio 1.018, I^2 = 3%

## Inconsistency

Results on the Mean Difference scale

Iterations = 20001:70000

Thinning interval = 1

Number of chains = 4

Sample size per chain = 50000

1. Empirical mean and standard deviation for each variable,

plus standard error of the mean:

Mean SD Naive SE Time-series SE

d.Cal.Cal_ElecAcu 1.902e-01 0.09385 2.098e-04 0.0002452

d.Cal.ElecAcu -9.828e-03 0.12352 2.762e-04 0.0003212

d.Cal.TCM_Mox 3.009e-02 0.13895 3.107e-04 0.0003742

d.CT_VD.CT_VD_ACE 5.930e-02 0.05967 1.334e-04 0.0001549

d.CT_VD.CT_VD_Acu 1.004e-02 0.06019 1.346e-04 0.0001467

d.CT_VD.CT_VD_Acu_GinMox 4.997e-02 0.13552 3.030e-04 0.0003310

d.CT_VD.CT_VD_MNKnife_Mox 9.895e-03 0.13273 2.968e-04 0.0003008

d.CT_VD.CT_VD_Mox 9.409e-05 0.13159 2.942e-04 0.0002912

d.CT_VD.CT_VD_TCM 3.451e-02 0.08597 1.922e-04 0.0002179

d.CT_VD.CT_VD_TCM_Acu 3.247e-01 0.09587 2.144e-04 0.0002359

d.CT_VD.CT_VD_TCM_FDMox 9.989e-02 0.13428 3.003e-04 0.0003177

d.CT_VD.CT_VD_TCM_Mox 3.996e-02 0.13330 2.981e-04 0.0003054

d.CT_VD.CT_VD_TFMox 1.978e-02 0.14052 3.142e-04 0.0003805

d.CT_VD.TCM_Mox 4.990e-02 0.13262 2.965e-04 0.0003041

d.CT_VD_Acu.CT_VD_BP -1.995e-02 0.13284 2.970e-04 0.0003176

d.CT_VD_Acu.CT_VD_ShortPri 7.778e-05 0.13332 2.981e-04 0.0003110

d.CT_VD_BP.CT_VD_BP_Acu 4.600e-02 0.05957 1.332e-04 0.0001360

d.CT_VD_BP.CT_VD_BP_Mox 1.976e-02 0.13325 2.980e-04 0.0003030

d.CT_VD_BP.CT_VD_BP_TCM_WarmAcu 2.959e-02 0.13233 2.959e-04 0.0003063

d.CT_VD_BP_Acu.CT_VD_BP_TCM_Acu 8.029e-02 0.13370 2.990e-04 0.0003135

d.CT_VD_TCM.CT_VD_TCM_Acu -4.963e-02 0.18086 4.044e-04 0.0013438

sd.d 1.290e-01 0.02796 6.253e-05 0.0001771

2. Quantiles for each variable:

2.5% 25% 50% 75% 97.5%

d.Cal.Cal_ElecAcu 0.00477 0.130211 0.1899501 0.25017 0.3779

d.Cal.ElecAcu -0.25498 -0.088488 -0.0095040 0.06914 0.2354

d.Cal.TCM_Mox -0.24507 -0.059016 0.0298827 0.11980 0.3052

d.CT_VD.CT_VD_ACE -0.05918 0.021117 0.0593466 0.09731 0.1783

d.CT_VD.CT_VD_Acu -0.10967 -0.028461 0.0101661 0.04859 0.1301

d.CT_VD.CT_VD_Acu_GinMox -0.21953 -0.036924 0.0497635 0.13616 0.3214

d.CT_VD.CT_VD_MNKnife_Mox -0.25387 -0.074764 0.0099873 0.09466 0.2737

d.CT_VD.CT_VD_Mox -0.26146 -0.084338 -0.0001862 0.08441 0.2624

d.CT_VD.CT_VD_TCM -0.13652 -0.020226 0.0344315 0.08906 0.2059

d.CT_VD.CT_VD_TCM_Acu 0.13857 0.262032 0.3233186 0.38558 0.5188

d.CT_VD.CT_VD_TCM_FDMox -0.16609 0.014274 0.0996208 0.18582 0.3669

d.CT_VD.CT_VD_TCM_Mox -0.22418 -0.044910 0.0399697 0.12502 0.3050

d.CT_VD.CT_VD_TFMox -0.25887 -0.070815 0.0195835 0.11027 0.2982

d.CT_VD.TCM_Mox -0.21366 -0.035011 0.0500309 0.13474 0.3137

d.CT_VD_Acu.CT_VD_BP -0.28364 -0.104732 -0.0199857 0.06493 0.2437

d.CT_VD_Acu.CT_VD_ShortPri -0.26542 -0.085329 -0.0002307 0.08542 0.2645

d.CT_VD_BP.CT_VD_BP_Acu -0.07265 0.007961 0.0460449 0.08407 0.1643

d.CT_VD_BP.CT_VD_BP_Mox -0.24518 -0.065415 0.0193976 0.10502 0.2855

d.CT_VD_BP.CT_VD_BP_TCM_WarmAcu -0.23446 -0.054660 0.0299796 0.11432 0.2909

d.CT_VD_BP_Acu.CT_VD_BP_TCM_Acu -0.18550 -0.005265 0.0805164 0.16554 0.3459

d.CT_VD_TCM.CT_VD_TCM_Acu -0.40499 -0.169626 -0.0502220 0.07052 0.3072

sd.d 0.08632 0.109343 0.1249677 0.14395 0.1950

-- Model fit (residual deviance):

Dbar pD DIC

69.62595 68.69530 138.32126

69 data points, ratio 1.009, I^2 = 2%

# FN-BMD (exculde BP)

## Consistency

Results on the Mean Difference scale

Iterations = 20001:70000

Thinning interval = 1

Number of chains = 4

Sample size per chain = 50000

1. Empirical mean and standard deviation for each variable,

plus standard error of the mean:

Mean SD Naive SE Time-series SE

d.CT_VD.CT_VD_ACE 0.05967 0.03628 8.113e-05 2.672e-04

d.CT_VD.CT_VD_Acu 0.01998 0.01638 3.663e-05 6.274e-05

d.CT_VD.CT_VD_MNKnife_Mox 0.01007 0.02993 6.692e-05 1.160e-04

d.CT_VD.CT_VD_TCM_FDMox 0.15057 0.04380 9.794e-05 4.411e-04

d.CT_VD.CT_VD_TCM_Mox 0.04977 0.03160 7.067e-05 1.541e-04

d.CT_VD.CT_VD_WarmAcu -0.01003 0.03270 7.311e-05 1.777e-04

sd.d 0.01829 0.02111 4.720e-05 4.074e-04

2. Quantiles for each variable:

2.5% 25% 50% 75% 97.5%

d.CT_VD.CT_VD_ACE -0.0094340 0.040031 0.059498 0.079238 0.12944

d.CT_VD.CT_VD_Acu -0.0132200 0.013602 0.020021 0.026263 0.05344

d.CT_VD.CT_VD_MNKnife_Mox -0.0489516 -0.001398 0.010173 0.021523 0.06893

d.CT_VD.CT_VD_TCM_FDMox 0.0659593 0.124392 0.150615 0.176842 0.23563

d.CT_VD.CT_VD_TCM_Mox -0.0113294 0.035603 0.049822 0.064103 0.11073

d.CT_VD.CT_VD_WarmAcu -0.0733017 -0.025369 -0.009939 0.005518 0.05325

sd.d 0.0004414 0.004805 0.011319 0.023356 0.08069

-- Model fit (residual deviance):

Dbar pD DIC

15.90237 15.90165 31.80402

18 data points, ratio 0.8835, I^2 = 0%

## Inconsistency

Results on the Mean Difference scale

Iterations = 20001:70000

Thinning interval = 1

Number of chains = 4

Sample size per chain = 50000

1. Empirical mean and standard deviation for each variable,

plus standard error of the mean:

Mean SD Naive SE Time-series SE

d.CT_VD.CT_VD_ACE 0.06042 0.03713 8.302e-05 2.630e-04

d.CT_VD.CT_VD_Acu 0.02013 0.01686 3.771e-05 6.307e-05

d.CT_VD.CT_VD_MNKnife_Mox 0.01012 0.03102 6.936e-05 1.187e-04

d.CT_VD.CT_VD_TCM_FDMox 0.14976 0.04518 1.010e-04 4.529e-04

d.CT_VD.CT_VD_TCM_Mox 0.04998 0.03278 7.330e-05 1.431e-04

d.CT_VD.CT_VD_WarmAcu -0.00989 0.03383 7.565e-05 1.784e-04

sd.d 0.01917 0.02193 4.904e-05 4.253e-04

2. Quantiles for each variable:

2.5% 25% 50% 75% 97.5%

d.CT_VD.CT_VD_ACE -0.0109825 0.040847 0.060495 0.07986 0.13207

d.CT_VD.CT_VD_Acu -0.0143170 0.013655 0.020156 0.02666 0.05435

d.CT_VD.CT_VD_MNKnife_Mox -0.0511047 -0.001747 0.010035 0.02193 0.07189

d.CT_VD.CT_VD_TCM_FDMox 0.0629115 0.122684 0.149542 0.17674 0.23693

d.CT_VD.CT_VD_TCM_Mox -0.0136755 0.035468 0.049991 0.06447 0.11399

d.CT_VD.CT_VD_WarmAcu -0.0745382 -0.025837 -0.009963 0.00593 0.05553

sd.d 0.0005952 0.005220 0.011882 0.02436 0.08506

-- Model fit (residual deviance):

Dbar pD DIC

16.01298 16.01237 32.02535

18 data points, ratio 0.8896, I^2 = 0%

# FN-BMD (include BP)

## Consistency

Results on the Mean Difference scale

Iterations = 20001:70000

Thinning interval = 1

Number of chains = 4

Sample size per chain = 50000

1. Empirical mean and standard deviation for each variable,

plus standard error of the mean:

Mean SD Naive SE Time-series SE

d.CT_VD_BP.CT_VD_BP_Acu 5.074e-02 0.02180 4.876e-05 7.045e-05

d.CT_VD_BP.CT_VD_BP_Acu_Pat 5.985e-02 0.04186 9.359e-05 2.236e-04

d.CT_VD_BP.CT_VD_BP_AcuInj -4.109e-05 0.03541 7.918e-05 8.057e-05

d.CT_VD_BP.CT_VD_BP_TCM_WarmAcu 2.003e-02 0.03848 8.604e-05 1.346e-04

d.CT_VD_BP_Acu.CT_VD_BP_TCM_Acu 1.098e-01 0.04206 9.404e-05 2.276e-04

d.CT_VD_BP_Acu.CT_VD_BP_TFMox 9.955e-03 0.03720 8.317e-05 1.132e-04

sd.d 2.549e-02 0.02453 5.485e-05 3.684e-04

2. Quantiles for each variable:

2.5% 25% 50% 75% 97.5%

d.CT_VD_BP.CT_VD_BP_Acu 0.0056381 0.041849 4.997e-02 0.058872 0.09946

d.CT_VD_BP.CT_VD_BP_Acu_Pat -0.0254395 0.038449 5.997e-02 0.081359 0.14569

d.CT_VD_BP.CT_VD_BP_AcuInj -0.0782368 -0.009594 1.808e-06 0.009566 0.07772

d.CT_VD_BP.CT_VD_BP_TCM_WarmAcu -0.0619991 0.003578 2.004e-02 0.036479 0.10190

d.CT_VD_BP_Acu.CT_VD_BP_TCM_Acu 0.0232109 0.088235 1.099e-01 0.131291 0.19525

d.CT_VD_BP_Acu.CT_VD_BP_TFMox -0.0712842 -0.004168 1.006e-02 0.024142 0.09042

sd.d 0.0007519 0.007363 1.715e-02 0.035511 0.09389

-- Model fit (residual deviance):

Dbar pD DIC

15.73785 15.31669 31.05455

16 data points, ratio 0.9836, I^2 = 5%

## Inconsistency

Results on the Mean Difference scale

Iterations = 20001:70000

Thinning interval = 1

Number of chains = 4

Sample size per chain = 50000

1. Empirical mean and standard deviation for each variable,

plus standard error of the mean:

Mean SD Naive SE Time-series SE

d.CT_VD_BP.CT_VD_BP_Acu 0.0506870 0.02154 4.816e-05 7.329e-05

d.CT_VD_BP.CT_VD_BP_Acu_Pat 0.0601047 0.04174 9.333e-05 2.231e-04

d.CT_VD_BP.CT_VD_BP_AcuInj 0.0000369 0.03498 7.823e-05 7.793e-05

d.CT_VD_BP.CT_VD_BP_TCM_WarmAcu 0.0199683 0.03756 8.399e-05 1.440e-04

d.CT_VD_BP_Acu.CT_VD_BP_TCM_Acu 0.1101290 0.04127 9.229e-05 2.158e-04

d.CT_VD_BP_Acu.CT_VD_BP_TFMox 0.0098709 0.03667 8.199e-05 1.046e-04

sd.d 0.0252685 0.02411 5.391e-05 3.571e-04

2. Quantiles for each variable:

2.5% 25% 50% 75% 97.5%

d.CT_VD_BP.CT_VD_BP_Acu 0.0063305 0.041643 4.987e-02 0.058823 0.09903

d.CT_VD_BP.CT_VD_BP_Acu_Pat -0.0255109 0.038423 6.014e-02 0.081932 0.14537

d.CT_VD_BP.CT_VD_BP_AcuInj -0.0772650 -0.009619 -5.072e-07 0.009692 0.07748

d.CT_VD_BP.CT_VD_BP_TCM_WarmAcu -0.0590982 0.003214 1.978e-02 0.036424 0.10045

d.CT_VD_BP_Acu.CT_VD_BP_TCM_Acu 0.0254395 0.089026 1.102e-01 0.131187 0.19448

d.CT_VD_BP_Acu.CT_VD_BP_TFMox -0.0694765 -0.004254 9.819e-03 0.024009 0.08910

sd.d 0.0008047 0.007430 1.717e-02 0.035231 0.09234

-- Model fit (residual deviance):

Dbar pD DIC

15.72845 15.30906 31.03750

16 data points, ratio 0.983, I^2 = 5%

# clinical efficacy

## Consistency

Results on the Log Risk Ratio scale

Iterations = 20001:70000

Thinning interval = 1

Number of chains = 4

Sample size per chain = 50000

1. Empirical mean and standard deviation for each variable,

plus standard error of the mean:

Mean SD Naive SE Time-series SE

d.ACE.E -0.33793 0.17907 0.0004004 0.0038056

d.ACE.TCM -0.12192 0.14033 0.0003138 0.0025549

d.ACE.TCM_ACE 0.03733 0.11460 0.0002563 0.0019424

d.Acu.TCM -0.12071 0.24454 0.0005468 0.0066373

d.Acu.TCM_Acu 0.29141 0.18749 0.0004192 0.0043431

d.BP.BP_ACE 0.11155 0.12277 0.0002745 0.0017652

d.BP.TCM_WarmAcu 0.63536 0.21049 0.0004707 0.0056221

d.CT_VD.CT_VD_ACE 0.32426 0.06603 0.0001477 0.0014155

d.CT_VD.CT_VD_Acu 0.19191 0.05893 0.0001318 0.0009550

d.CT_VD.CT_VD_Mox -0.22185 0.21151 0.0004729 0.0048196

d.CT_VD.CT_VD_TCM 0.28413 0.07480 0.0001673 0.0014573

d.CT_VD.CT_VD_TCM_Acu 0.22249 0.05789 0.0001294 0.0009657

d.CT_VD.CT_VD_TFMox 0.08920 0.14280 0.0003193 0.0022242

d.CT_VD.CT_VD_WarmAcu 0.29576 0.11558 0.0002585 0.0024227

d.CT_VD.TCM 0.21675 0.16033 0.0003585 0.0037857

d.CT_VD.TCM_Acu 0.51111 0.15144 0.0003386 0.0038953

d.CT_VD.TCM_FNeedle 0.29365 0.14974 0.0003348 0.0034072

d.CT_VD_Acu.CT_VD_BP -0.47707 0.17995 0.0004024 0.0032522

d.CT_VD_Acu.CT_VD_DuMox 0.14085 0.08488 0.0001898 0.0012861

d.CT_VD_BP.CT_VD_BP_Acu 0.15797 0.05209 0.0001165 0.0006112

d.CT_VD_BP.CT_VD_BP_Acu_Pat 0.18972 0.11132 0.0002489 0.0017510

d.CT_VD_BP.CT_VD_BP_Mox 0.18592 0.13719 0.0003068 0.0022417

d.CT_VD_BP.CT_VD_BP_Pat 0.31868 0.16353 0.0003657 0.0028965

d.CT_VD_BP.CT_VD_BP_TCM_ACE 0.32357 0.17758 0.0003971 0.0035347

d.CT_VD_BP_Acu.CT_VD_BP_Acu_Mox 0.18014 0.10530 0.0002355 0.0013829

d.CT_VD_BP_Acu.CT_VD_BP_TCM_Acu 0.17254 0.11101 0.0002482 0.0015468

d.TCM.TCM_Acu 0.31656 0.09789 0.0002189 0.0018961

d.TCM.TCM_WarmAcu 0.17428 0.07387 0.0001652 0.0009647

d.TCM_WarmAcu.WarmAcu -0.20131 0.10735 0.0002400 0.0011479

sd.d 0.04837 0.03430 0.0000767 0.0011109

2. Quantiles for each variable:

2.5% 25% 50% 75% 97.5%

d.ACE.E -0.711812 -0.449689 -0.33011 -0.21680 -0.013602

d.ACE.TCM -0.409579 -0.209226 -0.11849 -0.03018 0.147219

d.ACE.TCM_ACE -0.189317 -0.035961 0.03582 0.10975 0.268698

d.Acu.TCM -0.603622 -0.284010 -0.11618 0.04394 0.353371

d.Acu.TCM_Acu -0.057553 0.165181 0.28390 0.41066 0.680998

d.BP.BP_ACE -0.123752 0.032686 0.10622 0.18654 0.367094

d.BP.TCM_WarmAcu 0.258941 0.493174 0.62307 0.76287 1.092534

d.CT_VD.CT_VD_ACE 0.202845 0.278706 0.32170 0.36667 0.462487

d.CT_VD.CT_VD_Acu 0.086585 0.151064 0.18900 0.22937 0.316167

d.CT_VD.CT_VD_Mox -0.666039 -0.357657 -0.21240 -0.07661 0.170080

d.CT_VD.CT_VD_TCM 0.137993 0.235313 0.28312 0.33238 0.434938

d.CT_VD.CT_VD_TCM_Acu 0.118146 0.183153 0.22004 0.25875 0.345143

d.CT_VD.CT_VD_TFMox -0.187653 -0.003484 0.08517 0.17927 0.380264

d.CT_VD.CT_VD_WarmAcu 0.081401 0.216831 0.29025 0.36819 0.537578

d.CT_VD.TCM -0.081586 0.107834 0.20642 0.31787 0.553692

d.CT_VD.TCM_Acu 0.239998 0.404669 0.50176 0.60781 0.832420

d.CT_VD.TCM_FNeedle 0.022506 0.189855 0.28521 0.38877 0.606781

d.CT_VD_Acu.CT_VD_BP -0.850421 -0.593647 -0.46699 -0.35279 -0.151274

d.CT_VD_Acu.CT_VD_DuMox -0.016811 0.084168 0.13731 0.19398 0.316843

d.CT_VD_BP.CT_VD_BP_Acu 0.064279 0.123332 0.15507 0.18921 0.269256

d.CT_VD_BP.CT_VD_BP_Acu_Pat -0.016791 0.114725 0.18432 0.25912 0.419445

d.CT_VD_BP.CT_VD_BP_Mox -0.075315 0.095644 0.18300 0.26993 0.471835

d.CT_VD_BP.CT_VD_BP_Pat 0.007793 0.209243 0.31411 0.42412 0.652195

d.CT_VD_BP.CT_VD_BP_TCM_ACE 0.004497 0.201704 0.31083 0.43393 0.705617

d.CT_VD_BP_Acu.CT_VD_BP_Acu_Mox -0.020961 0.112038 0.17699 0.24652 0.397142

d.CT_VD_BP_Acu.CT_VD_BP_TCM_Acu -0.038542 0.099843 0.16818 0.24060 0.404385

d.TCM.TCM_Acu 0.132226 0.250201 0.31421 0.37954 0.517463

d.TCM.TCM_WarmAcu 0.034043 0.125015 0.17237 0.22117 0.324716

d.TCM_WarmAcu.WarmAcu -0.429202 -0.267386 -0.19641 -0.13075 -0.001319

sd.d 0.002036 0.021011 0.04298 0.06912 0.127822

-- Model fit (residual deviance):

Dbar pD DIC

93.40809 63.54977 156.95786

91 data points, ratio 1.026, I^2 = 4%

## Inconsistency

Results on the Log Risk Ratio scale

Iterations = 20001:70000

Thinning interval = 1

Number of chains = 4

Sample size per chain = 50000

1. Empirical mean and standard deviation for each variable,

plus standard error of the mean:

Mean SD Naive SE Time-series SE

d.BP.BP_ACE 0.11339 0.12184 2.724e-04 0.0018941

d.CT_VD.CT_VD_ACE 0.32147 0.06506 1.455e-04 0.0015134

d.CT_VD.CT_VD_Acu 0.19142 0.05802 1.297e-04 0.0009827

d.CT_VD.CT_VD_Mox -0.22770 0.20946 4.684e-04 0.0050125

d.CT_VD.CT_VD_TCM 0.28329 0.07254 1.622e-04 0.0015233

d.CT_VD.CT_VD_TCM_Acu 0.22251 0.05656 1.265e-04 0.0009735

d.CT_VD.CT_VD_TFMox 0.08976 0.14359 3.211e-04 0.0024903

d.CT_VD.CT_VD_WarmAcu 0.30165 0.11505 2.573e-04 0.0025065

d.CT_VD.TCM 0.19177 0.11673 2.610e-04 0.0033510

d.CT_VD.TCM_Acu 0.51808 0.11459 2.562e-04 0.0033846

d.CT_VD.TCM_FNeedle 0.27023 0.12985 2.904e-04 0.0030099

d.CT_VD_Acu.CT_VD_BP -0.47012 0.17614 3.939e-04 0.0031796

d.CT_VD_Acu.CT_VD_DuMox 0.14236 0.08338 1.864e-04 0.0012784

d.CT_VD_BP.CT_VD_BP_Acu 0.15729 0.04985 1.115e-04 0.0005757

d.CT_VD_BP.CT_VD_BP_Acu_Pat 0.18947 0.10746 2.403e-04 0.0016227

d.CT_VD_BP.CT_VD_BP_Mox 0.18226 0.13168 2.944e-04 0.0021998

d.CT_VD_BP.CT_VD_BP_Pat 0.32532 0.16329 3.651e-04 0.0030031

d.CT_VD_BP.CT_VD_BP_TCM_ACE 0.31838 0.17093 3.822e-04 0.0034286

d.CT_VD_BP_Acu.CT_VD_BP_Acu_Mox 0.18061 0.10008 2.238e-04 0.0013002

d.CT_VD_BP_Acu.CT_VD_BP_TCM_Acu 0.17662 0.11003 2.460e-04 0.0016807

d.TCM.ACE 0.12591 0.14267 3.190e-04 0.0030605

d.TCM.Acu 0.03833 0.19099 4.271e-04 0.0054593

d.TCM.E -0.20813 0.19418 4.342e-04 0.0042815

d.TCM.TCM_ACE 0.16522 0.13409 2.998e-04 0.0024439

d.TCM.TCM_WarmAcu 0.17370 0.07127 1.594e-04 0.0010142

d.TCM_WarmAcu.BP -0.64955 0.20744 4.638e-04 0.0057764

d.TCM_WarmAcu.WarmAcu -0.20076 0.10792 2.413e-04 0.0013643

sd.d 0.04446 0.03227 7.216e-05 0.0010492

2. Quantiles for each variable:

2.5% 25% 50% 75% 97.5%

d.BP.BP_ACE -0.122205 0.034643 0.10976 0.18897 0.3635340

d.CT_VD.CT_VD_ACE 0.202359 0.276035 0.31875 0.36276 0.4576737

d.CT_VD.CT_VD_Acu 0.084576 0.151936 0.18877 0.22786 0.3136844

d.CT_VD.CT_VD_Mox -0.674256 -0.362941 -0.21529 -0.08293 0.1539085

d.CT_VD.CT_VD_TCM 0.143626 0.235121 0.28283 0.32866 0.4343075

d.CT_VD.CT_VD_TCM_Acu 0.120110 0.183980 0.21934 0.25766 0.3409727

d.CT_VD.CT_VD_TFMox -0.185407 -0.004081 0.08416 0.18108 0.3858651

d.CT_VD.CT_VD_WarmAcu 0.087954 0.224410 0.29655 0.37262 0.5447256

d.CT_VD.TCM -0.030138 0.114751 0.18915 0.26678 0.4274466

d.CT_VD.TCM_Acu 0.303099 0.438720 0.51606 0.59311 0.7474428

d.CT_VD.TCM_FNeedle 0.022246 0.184614 0.26716 0.35251 0.5343538

d.CT_VD_Acu.CT_VD_BP -0.840174 -0.581193 -0.46246 -0.34884 -0.1447272

d.CT_VD_Acu.CT_VD_DuMox -0.013355 0.087202 0.13900 0.19466 0.3154786

d.CT_VD_BP.CT_VD_BP_Acu 0.065661 0.124637 0.15500 0.18701 0.2633715

d.CT_VD_BP.CT_VD_BP_Acu_Pat -0.011954 0.119329 0.18433 0.25626 0.4147481

d.CT_VD_BP.CT_VD_BP_Mox -0.071571 0.095777 0.17944 0.26398 0.4554186

d.CT_VD_BP.CT_VD_BP_Pat 0.014639 0.215561 0.32100 0.43065 0.6598052

d.CT_VD_BP.CT_VD_BP_TCM_ACE 0.008561 0.204625 0.30895 0.42367 0.6795075

d.CT_VD_BP_Acu.CT_VD_BP_Acu_Mox -0.010386 0.115991 0.17758 0.24419 0.3843045

d.CT_VD_BP_Acu.CT_VD_BP_TCM_Acu -0.031002 0.103588 0.17302 0.24524 0.4052909

d.TCM.ACE -0.148928 0.031777 0.12243 0.21564 0.4184768

d.TCM.Acu -0.347793 -0.087486 0.04026 0.16822 0.4062274

d.TCM.E -0.609756 -0.329239 -0.19976 -0.07593 0.1618313

d.TCM.TCM_ACE -0.087954 0.075839 0.16017 0.24898 0.4491201

d.TCM.TCM_WarmAcu 0.038509 0.126975 0.17144 0.21785 0.3205243

d.TCM_WarmAcu.BP -1.084757 -0.781422 -0.63596 -0.50404 -0.2774857

d.TCM_WarmAcu.WarmAcu -0.430924 -0.266751 -0.19551 -0.12963 -0.0007229

sd.d 0.002489 0.019072 0.03846 0.06325 0.1212731

-- Model fit (residual deviance):

Dbar pD DIC

91.86675 61.46192 153.32867

91 data points, ratio 1.01, I^2 = 2%

# The total score of TCM syndrome

## Consistency

Results on the Mean Difference scale

Iterations = 20001:70000

Thinning interval = 1

Number of chains = 4

Sample size per chain = 50000

1. Empirical mean and standard deviation for each variable,

plus standard error of the mean:

Mean SD Naive SE Time-series SE

d.CT_VD.CT_VD_ACE -2.4119 1.2804 0.002863 0.003394

d.CT_VD.CT_VD_Mox -1.9881 2.2633 0.005061 0.005135

d.CT_VD.CT_VD_TCM_FDMox -1.2279 2.3556 0.005267 0.006162

d.CT_VD.CT_VD_TFMox -2.7525 2.4089 0.005386 0.006689

d.CT_VD_Acu.CT_VD 3.7937 1.0351 0.002315 0.003917

d.CT_VD_Acu.CT_VD_BP 4.0938 2.4988 0.005587 0.007758

d.CT_VD_Acu.CT_VD_DuMox -2.5406 1.6923 0.003784 0.004875

d.CT_VD_Acu.CT_VD_ShortPri -2.4326 2.3921 0.005349 0.006809

d.CT_VD_Acu.CT_VD_TCM 2.8793 1.4825 0.003315 0.005416

d.CT_VD_Acu.CT_VD_TCM_Acu -0.4369 1.5233 0.003406 0.005331

d.CT_VD_BP.CT_VD_BP_Acu -2.6683 1.9240 0.004302 0.006217

d.CT_VD_BP.CT_VD_BP_Acu_DuMox -0.8020 2.3004 0.005144 0.005499

d.CT_VD_BP.CT_VD_BP_Mox -2.4955 2.4237 0.005420 0.006910

d.CT_VD_BP.CT_VD_BP_Pat 2.7034 2.5033 0.005598 0.007839

d.CT_VD_BP.CT_VD_BP_TCM_Acu -5.2862 2.0118 0.004498 0.006957

sd.d 2.1809 0.5718 0.001279 0.003779

2. Quantiles for each variable:

2.5% 25% 50% 75% 97.5%

d.CT_VD.CT_VD_ACE -4.95940 -3.212 -2.4141 -1.6131 0.1401

d.CT_VD.CT_VD_Mox -6.52201 -3.395 -1.9890 -0.5810 2.5297

d.CT_VD.CT_VD_TCM_FDMox -5.88614 -2.718 -1.2317 0.2587 3.4678

d.CT_VD.CT_VD_TFMox -7.55525 -4.287 -2.7470 -1.2188 2.0204

d.CT_VD_Acu.CT_VD 1.73229 3.142 3.7895 4.4422 5.8616

d.CT_VD_Acu.CT_VD_BP -0.87434 2.496 4.1012 5.6977 9.0243

d.CT_VD_Acu.CT_VD_DuMox -5.90778 -3.614 -2.5414 -1.4710 0.8131

d.CT_VD_Acu.CT_VD_ShortPri -7.17110 -3.955 -2.4328 -0.9205 2.3336

d.CT_VD_Acu.CT_VD_TCM -0.06879 1.948 2.8775 3.8075 5.8476

d.CT_VD_Acu.CT_VD_TCM_Acu -3.50202 -1.391 -0.4232 0.5298 2.5506

d.CT_VD_BP.CT_VD_BP_Acu -6.52585 -3.872 -2.6656 -1.4568 1.1378

d.CT_VD_BP.CT_VD_BP_Acu_DuMox -5.40367 -2.238 -0.7998 0.6386 3.7635

d.CT_VD_BP.CT_VD_BP_Mox -7.32032 -4.030 -2.4906 -0.9558 2.3250

d.CT_VD_BP.CT_VD_BP_Pat -2.24105 1.099 2.7024 4.3101 7.6663

d.CT_VD_BP.CT_VD_BP_TCM_Acu -9.28189 -6.567 -5.2825 -4.0043 -1.2969

sd.d 1.36268 1.781 2.0816 2.4664 3.5901

-- Model fit (residual deviance):

Dbar pD DIC

48.73443 48.23613 96.97056

49 data points, ratio 0.9946, I^2 = 2%

## Inconsistency

Results on the Mean Difference scale

Iterations = 20001:70000

Thinning interval = 1

Number of chains = 4

Sample size per chain = 50000

1. Empirical mean and standard deviation for each variable,

plus standard error of the mean:

Mean SD Naive SE Time-series SE

d.CT_VD.CT_VD_ACE -2.8072 1.3201 0.002952 0.003781

d.CT_VD.CT_VD_Acu -3.8528 1.1617 0.002598 0.002923

d.CT_VD.CT_VD_Mox -1.9943 2.2644 0.005063 0.005229

d.CT_VD.CT_VD_TCM -1.9969 1.5502 0.003466 0.004394

d.CT_VD.CT_VD_TCM_Acu -2.7473 1.6920 0.003783 0.004849

d.CT_VD.CT_VD_TCM_FDMox -1.2399 2.3492 0.005253 0.006204

d.CT_VD.CT_VD_TFMox -2.7353 2.3979 0.005362 0.006757

d.CT_VD_Acu.CT_VD_BP 4.0897 2.5017 0.005594 0.007638

d.CT_VD_Acu.CT_VD_DuMox -2.5378 1.6907 0.003780 0.004920

d.CT_VD_Acu.CT_VD_ShortPri -2.4228 2.3859 0.005335 0.006867

d.CT_VD_Acu.CT_VD_TCM 4.0033 2.3222 0.005193 0.007137

d.CT_VD_Acu.CT_VD_TCM_Acu -1.9960 2.3164 0.005180 0.006921

d.CT_VD_BP.CT_VD_BP_Acu -2.4947 2.2996 0.005142 0.005609

d.CT_VD_BP.CT_VD_BP_Acu_DuMox -0.7943 2.2934 0.005128 0.005497

d.CT_VD_BP.CT_VD_BP_Mox -2.5009 2.4182 0.005407 0.006890

d.CT_VD_BP.CT_VD_BP_Pat 2.6966 2.5013 0.005593 0.007827

d.CT_VD_BP.CT_VD_BP_TCM_Acu -5.4893 2.5465 0.005694 0.007806

d.CT_VD_BP_Acu.CT_VD_BP_TCM_Acu -2.4357 2.3111 0.005168 0.005753

sd.d 2.1391 0.7071 0.001581 0.005890

2. Quantiles for each variable:

2.5% 25% 50% 75% 97.5%

d.CT_VD.CT_VD_ACE -5.4560 -3.598 -2.809 -2.0163 -0.1442

d.CT_VD.CT_VD_Acu -6.1962 -4.555 -3.848 -3.1449 -1.5224

d.CT_VD.CT_VD_Mox -6.5668 -3.342 -1.993 -0.6356 2.5549

d.CT_VD.CT_VD_TCM -5.1027 -2.927 -2.000 -1.0707 1.1420

d.CT_VD.CT_VD_TCM_Acu -6.2500 -3.759 -2.704 -1.6904 0.5274

d.CT_VD.CT_VD_TCM_FDMox -5.9537 -2.670 -1.240 0.1915 3.4645

d.CT_VD.CT_VD_TFMox -7.5243 -4.210 -2.738 -1.2638 2.0806

d.CT_VD_Acu.CT_VD_BP -0.9279 2.543 4.096 5.6470 9.0617

d.CT_VD_Acu.CT_VD_DuMox -5.9114 -3.576 -2.537 -1.5047 0.8475

d.CT_VD_Acu.CT_VD_ShortPri -7.1912 -3.882 -2.422 -0.9608 2.3359

d.CT_VD_Acu.CT_VD_TCM -0.6665 2.598 4.001 5.4041 8.6381

d.CT_VD_Acu.CT_VD_TCM_Acu -6.6894 -3.394 -1.991 -0.5991 2.6517

d.CT_VD_BP.CT_VD_BP_Acu -7.1088 -3.883 -2.492 -1.1059 2.1206

d.CT_VD_BP.CT_VD_BP_Acu_DuMox -5.3872 -2.169 -0.803 0.5893 3.8199

d.CT_VD_BP.CT_VD_BP_Mox -7.3365 -3.983 -2.499 -1.0112 2.3218

d.CT_VD_BP.CT_VD_BP_Pat -2.2880 1.135 2.700 4.2492 7.7146

d.CT_VD_BP.CT_VD_BP_TCM_Acu -10.5106 -7.093 -5.494 -3.8929 -0.3855

d.CT_VD_BP_Acu.CT_VD_BP_TCM_Acu -7.0860 -3.825 -2.432 -1.0474 2.1868

sd.d 1.1744 1.642 1.998 2.4777 3.9317

-- Model fit (residual deviance):

Dbar pD DIC

49.13776 48.46378 97.60154

49 data points, ratio 1.003, I^2 = 2%

# The low back pain score of TCM syndrome (exclude BP)

## Consistency

Results on the Mean Difference scale

Iterations = 20001:70000

Thinning interval = 1

Number of chains = 4

Sample size per chain = 50000

1. Empirical mean and standard deviation for each variable,

plus standard error of the mean:

Mean SD Naive SE Time-series SE

d.CT_VD.CT_VD_Mox -0.05251 0.7667 0.001714 0.002016

d.CT_VD.CT_VD_Pat -1.57769 1.1999 0.002683 0.005657

d.CT_VD.CT_VD_TCM -0.08926 1.0198 0.002280 0.002739

d.CT_VD.CT_VD_TCM_Acu -0.63644 1.0916 0.002441 0.002622

d.CT_VD.CT_VD_TCM_Mox -0.17279 1.0827 0.002421 0.002442

d.CT_VD.CT_VD_TFMox -0.49649 1.0929 0.002444 0.002731

d.CT_VD.CT_VD_WarmAcu -2.93766 1.1299 0.002526 0.003527

d.CT_VD.TCM_Acu -0.63625 1.0806 0.002416 0.002332

d.TCM_Acu.TCM 1.42589 1.1678 0.002611 0.004855

sd.d 0.76211 0.7673 0.001716 0.014653

2. Quantiles for each variable:

2.5% 25% 50% 75% 97.5%

d.CT_VD.CT_VD_Mox -1.82044 -0.2433 -0.03234 0.12965 1.6913

d.CT_VD.CT_VD_Pat -4.16372 -2.1303 -1.57747 -1.02720 1.0108

d.CT_VD.CT_VD_TCM -2.41959 -0.3590 -0.08010 0.17984 2.2164

d.CT_VD.CT_VD_TCM_Acu -3.10190 -0.9191 -0.64029 -0.35941 1.8851

d.CT_VD.CT_VD_TCM_Mox -2.64309 -0.4266 -0.16954 0.08578 2.2927

d.CT_VD.CT_VD_TFMox -3.00433 -0.8107 -0.49311 -0.17956 1.9831

d.CT_VD.CT_VD_WarmAcu -5.46947 -3.3448 -2.93320 -2.52430 -0.4256

d.CT_VD.TCM_Acu -3.09825 -0.8877 -0.63900 -0.38968 1.8307

d.TCM_Acu.TCM -1.12808 0.9370 1.42650 1.91457 3.9805

sd.d 0.01486 0.1578 0.45466 1.17230 2.6789

-- Model fit (residual deviance):

Dbar pD DIC

18.83444 18.80142 37.63586

19 data points, ratio 0.9913, I^2 = 4%

## Inconsistency

Results on the Mean Difference scale

Iterations = 20001:70000

Thinning interval = 1

Number of chains = 4

Sample size per chain = 50000

1. Empirical mean and standard deviation for each variable,

plus standard error of the mean:

Mean SD Naive SE Time-series SE

d.CT_VD.CT_VD_Mox -0.05313 0.7774 0.001738 0.002053

d.CT_VD.CT_VD_Pat -1.56082 1.2090 0.002703 0.005713

d.CT_VD.CT_VD_TCM -0.08996 1.0284 0.002300 0.002766

d.CT_VD.CT_VD_TCM_Acu -0.63508 1.0936 0.002445 0.002535

d.CT_VD.CT_VD_TCM_Mox -0.17222 1.0923 0.002442 0.002533

d.CT_VD.CT_VD_TFMox -0.50165 1.1030 0.002466 0.002722

d.CT_VD.CT_VD_WarmAcu -2.93551 1.1351 0.002538 0.003668

d.CT_VD.TCM_Acu -0.64244 1.0817 0.002419 0.002368

d.TCM.TCM_Acu -1.43070 1.1740 0.002625 0.004656

sd.d 0.76885 0.7691 0.001720 0.015085

2. Quantiles for each variable:

2.5% 25% 50% 75% 97.5%

d.CT_VD.CT_VD_Mox -1.82735 -0.2492 -0.03407 0.1315 1.7198

d.CT_VD.CT_VD_Pat -4.16605 -2.1110 -1.55877 -1.0103 1.0663

d.CT_VD.CT_VD_TCM -2.42116 -0.3642 -0.08323 0.1790 2.2629

d.CT_VD.CT_VD_TCM_Acu -3.10204 -0.9251 -0.63803 -0.3541 1.8731

d.CT_VD.CT_VD_TCM_Mox -2.68428 -0.4360 -0.17057 0.0906 2.3160

d.CT_VD.CT_VD_TFMox -2.99847 -0.8187 -0.49985 -0.1832 2.0044

d.CT_VD.CT_VD_WarmAcu -5.46553 -3.3570 -2.93687 -2.5171 -0.3994

d.CT_VD.TCM_Acu -3.13233 -0.8933 -0.64029 -0.3882 1.8292

d.TCM.TCM_Acu -3.98615 -1.9241 -1.42703 -0.9421 1.1380

sd.d 0.01471 0.1610 0.46429 1.1845 2.6796

-- Model fit (residual deviance):

Dbar pD DIC

18.90021 18.86832 37.76853

19 data points, ratio 0.9947, I^2 = 5%

# The low back pain score of TCM syndrome (include BP)

## Consistency

Results on the Mean Difference scale

Iterations = 20001:70000

Thinning interval = 1

Number of chains = 4

Sample size per chain = 50000

1. Empirical mean and standard deviation for each variable,

plus standard error of the mean:

Mean SD Naive SE Time-series SE

d.CT_VD_BP.CT_VD_BP_Acu -0.4334 0.3263 0.0007297 0.0007437

d.CT_VD_BP.CT_VD_BP_Mox 0.0833 0.5569 0.0012454 0.0010447

d.CT_VD_BP.CT_VD_BP_TCM_ACE -0.7202 0.4827 0.0010793 0.0015109

d.CT_VD_BP.CT_VD_BP_TCM_WarmAcu -0.8391 0.4632 0.0010359 0.0010793

d.CT_VD_BP_Acu.CT_VD_BP_Acu_Mox -1.2200 0.4785 0.0010700 0.0014446

d.CT_VD_BP_Acu.CT_VD_BP_TFMox -0.1689 0.4637 0.0010368 0.0010760

sd.d 0.3298 0.3206 0.0007170 0.0050216

2. Quantiles for each variable:

2.5% 25% 50% 75% 97.5%

d.CT_VD_BP.CT_VD_BP_Acu -1.178009 -0.52152 -0.43317 -0.34605 0.3155

d.CT_VD_BP.CT_VD_BP_Mox -1.073916 -0.27948 0.09648 0.42559 1.2194

d.CT_VD_BP.CT_VD_BP_TCM_ACE -1.783840 -0.90414 -0.71897 -0.53857 0.3544

d.CT_VD_BP.CT_VD_BP_TCM_WarmAcu -1.885180 -0.96336 -0.84088 -0.71513 0.2265

d.CT_VD_BP_Acu.CT_VD_BP_Acu_Mox -2.296198 -1.39435 -1.21613 -1.04430 -0.1594

d.CT_VD_BP_Acu.CT_VD_BP_TFMox -1.220405 -0.29596 -0.16918 -0.04344 0.8944

sd.d 0.006444 0.07375 0.20819 0.50916 1.1153

-- Model fit (residual deviance):

Dbar pD DIC

19.41667 18.95496 38.37164

14 data points, ratio 1.387, I^2 = 33%

## Inconsistency

Results on the Mean Difference scale

Iterations = 20001:70000

Thinning interval = 1

Number of chains = 4

Sample size per chain = 50000

1. Empirical mean and standard deviation for each variable,

plus standard error of the mean:

Mean SD Naive SE Time-series SE

d.CT_VD_BP.CT_VD_BP_Acu -0.4331 0.3287 0.0007351 0.0007508

d.CT_VD_BP.CT_VD_BP_Mox -0.3655 0.4934 0.0011033 0.0010390

d.CT_VD_BP.CT_VD_BP_TCM_ACE -0.7208 0.4835 0.0010810 0.0014352

d.CT_VD_BP.CT_VD_BP_TCM_WarmAcu -0.8390 0.4661 0.0010423 0.0010596

d.CT_VD_BP_Acu.CT_VD_BP_Acu_Mox -1.2199 0.4805 0.0010744 0.0013764

d.CT_VD_BP_Acu.CT_VD_BP_TFMox -0.1700 0.4652 0.0010403 0.0011078

sd.d 0.3337 0.3198 0.0007151 0.0049302

2. Quantiles for each variable:

2.5% 25% 50% 75% 97.5%

d.CT_VD_BP.CT_VD_BP_Acu -1.181164 -0.52326 -0.4329 -0.34297 0.3138

d.CT_VD_BP.CT_VD_BP_Mox -1.452382 -0.54164 -0.4150 -0.10929 0.7224

d.CT_VD_BP.CT_VD_BP_TCM_ACE -1.799667 -0.90352 -0.7230 -0.53733 0.3529

d.CT_VD_BP.CT_VD_BP_TCM_WarmAcu -1.893894 -0.96657 -0.8400 -0.71178 0.2218

d.CT_VD_BP_Acu.CT_VD_BP_Acu_Mox -2.289592 -1.39950 -1.2206 -1.04393 -0.1396

d.CT_VD_BP_Acu.CT_VD_BP_TFMox -1.227974 -0.29882 -0.1692 -0.04018 0.8828

sd.d 0.007448 0.07809 0.2143 0.51216 1.1173

-- Model fit (residual deviance):

Dbar pD DIC

22.70239 14.67381 37.37621

14 data points, ratio 1.622, I^2 = 43%

# VAS score

## Consistency

Results on the Mean Difference scale

Iterations = 20001:70000

Thinning interval = 1

Number of chains = 4

Sample size per chain = 50000

1. Empirical mean and standard deviation for each variable,

plus standard error of the mean:

Mean SD Naive SE Time-series SE

d.CT_VD.CT_VD_ACE -1.4347 0.3741 0.0008364 0.001065

d.CT_VD.CT_VD_Acu -1.6896 0.3177 0.0007104 0.001141

d.CT_VD.CT_VD_ChenpiDuMox -3.0184 0.6699 0.0014978 0.002299

d.CT_VD.CT_VD_DuMox -2.4921 0.3945 0.0008822 0.001501

d.CT_VD.CT_VD_MNKnife_Mox -1.1982 0.6989 0.0015629 0.002070

d.CT_VD.CT_VD_Mox -0.4530 0.4728 0.0010571 0.001508

d.CT_VD.CT_VD_TCM -1.0718 0.3811 0.0008521 0.001280

d.CT_VD.CT_VD_TFMox -1.0267 0.7787 0.0017413 0.003152

d.CT_VD.CT_VD_WarmAcu -1.2163 0.6988 0.0015626 0.002151

d.CT_VD.TCM -1.0990 0.6428 0.0014373 0.001908

d.CT_VD.TCM_FNeedle -1.7004 0.6410 0.0014332 0.001886

d.CT_VD.TCM_Mox 1.8070 0.6965 0.0015574 0.002100

d.CT_VD_Acu.CT_VD_BP 0.7306 0.6786 0.0015174 0.001887

d.CT_VD_Acu.CT_VD_ShortPri -1.1081 0.7099 0.0015873 0.002287

d.CT_VD_Acu.CT_VD_TCM_Acu -0.9408 0.6395 0.0014299 0.001962

d.CT_VD_BP.CT_VD_BP_Acu -1.4848 0.4944 0.0011055 0.001482

d.CT_VD_BP.CT_VD_BP_Acu_Pat -2.1404 0.6505 0.0014545 0.001636

d.CT_VD_BP.CT_VD_BP_Pat -1.1937 0.6892 0.0015410 0.002123

d.CT_VD_BP_Acu.CT_VD_BP_Acu_Mox -0.6390 0.6417 0.0014349 0.001533

d.CT_VD_BP_Acu.CT_VD_BP_TCM_Acu -0.8875 0.6675 0.0014927 0.001850

d.TCM.ACE -0.4825 0.7432 0.0016618 0.003683

d.TCM.DuMox 1.4895 0.7211 0.0016124 0.002884

d.TCM.E 1.3511 0.7536 0.0016851 0.003245

d.TCM.TCM_ACE -1.0698 0.7427 0.0016608 0.003103

d.TCM.TCM_Acu -0.6914 0.6680 0.0014936 0.001849

d.TCM.TCM_DuMox -1.0482 0.7308 0.0016340 0.002648

d.TCM.TCM_WarmAcu -0.9305 0.6616 0.0014794 0.001708

d.TCM_Acu.Cel 0.8401 0.6463 0.0014451 0.001577

sd.d 0.6019 0.1891 0.0004228 0.001913

2. Quantiles for each variable:

2.5% 25% 50% 75% 97.5%

d.CT_VD.CT_VD_ACE -2.18339 -1.6663 -1.4347 -1.20396 -0.6867

d.CT_VD.CT_VD_Acu -2.31297 -1.8905 -1.6934 -1.49428 -1.0426

d.CT_VD.CT_VD_ChenpiDuMox -4.34758 -3.4433 -3.0189 -2.59359 -1.6858

d.CT_VD.CT_VD_DuMox -3.27494 -2.7410 -2.4927 -2.24251 -1.7086

d.CT_VD.CT_VD_MNKnife_Mox -2.59053 -1.6366 -1.1983 -0.76083 0.1938

d.CT_VD.CT_VD_Mox -1.39837 -0.7477 -0.4523 -0.15483 0.4784

d.CT_VD.CT_VD_TCM -1.83984 -1.3072 -1.0696 -0.83526 -0.3129

d.CT_VD.CT_VD_TFMox -2.56974 -1.5263 -1.0267 -0.52669 0.5110

d.CT_VD.CT_VD_WarmAcu -2.60880 -1.6555 -1.2154 -0.77776 0.1727

d.CT_VD.TCM -2.39282 -1.4907 -1.0973 -0.70688 0.1893

d.CT_VD.TCM_FNeedle -2.99099 -2.0894 -1.7002 -1.31071 -0.4137

d.CT_VD.TCM_Mox 0.41662 1.3712 1.8066 2.24180 3.1947

d.CT_VD_Acu.CT_VD_BP -0.62299 0.3099 0.7314 1.15354 2.0793

d.CT_VD_Acu.CT_VD_ShortPri -2.52150 -1.5542 -1.1097 -0.66436 0.3085

d.CT_VD_Acu.CT_VD_TCM_Acu -2.21926 -1.3375 -0.9383 -0.53850 0.3252

d.CT_VD_BP.CT_VD_BP_Acu -2.48715 -1.7921 -1.4781 -1.17319 -0.5208

d.CT_VD_BP.CT_VD_BP_Acu_Pat -3.44220 -2.5379 -2.1399 -1.74135 -0.8373

d.CT_VD_BP.CT_VD_BP_Pat -2.56967 -1.6239 -1.1943 -0.76329 0.1837

d.CT_VD_BP_Acu.CT_VD_BP_Acu_Mox -1.93488 -1.0284 -0.6389 -0.25132 0.6532

d.CT_VD_BP_Acu.CT_VD_BP_TCM_Acu -2.22418 -1.3005 -0.8873 -0.47449 0.4494

d.TCM.ACE -1.95558 -0.9569 -0.4837 -0.00762 0.9932

d.TCM.DuMox 0.06107 1.0336 1.4874 1.94538 2.9268

d.TCM.E -0.14324 0.8702 1.3507 1.83272 2.8442

d.TCM.TCM_ACE -2.54120 -1.5435 -1.0707 -0.59714 0.4071

d.TCM.TCM_Acu -2.02626 -1.1022 -0.6917 -0.27981 0.6513

d.TCM.TCM_DuMox -2.49725 -1.5121 -1.0475 -0.58679 0.4053

d.TCM.TCM_WarmAcu -2.26133 -1.3363 -0.9314 -0.52211 0.3909

d.TCM_Acu.Cel -0.46158 0.4476 0.8393 1.23290 2.1355

sd.d 0.31883 0.4709 0.5726 0.70032 1.0488

-- Model fit (residual deviance):

Dbar pD DIC

70.42464 68.07639 138.50103

70 data points, ratio 1.006, I^2 = 2%

## Inconsistency

Results on the Mean Difference scale

Iterations = 20001:70000

Thinning interval = 1

Number of chains = 4

Sample size per chain = 50000

1. Empirical mean and standard deviation for each variable,

plus standard error of the mean:

Mean SD Naive SE Time-series SE

d.ACE.E 1.8316 0.6541 0.0014626 0.004012

d.ACE.TCM 0.4808 0.6435 0.0014388 0.003830

d.ACE.TCM_ACE -0.5923 0.6435 0.0014389 0.003840

d.Cel.TCM_Acu -0.8406 0.5244 0.0011726 0.001352

d.CT_VD.CT_VD_ACE -1.5256 0.3114 0.0006963 0.001014

d.CT_VD.CT_VD_Acu -1.5051 0.3266 0.0007303 0.001072

d.CT_VD.CT_VD_ChenpiDuMox -2.9197 0.5919 0.0013235 0.002546

d.CT_VD.CT_VD_DuMox -2.2708 0.4331 0.0009684 0.001852

d.CT_VD.CT_VD_MNKnife_Mox -1.2020 0.5878 0.0013143 0.002001

d.CT_VD.CT_VD_Mox -0.5661 0.4046 0.0009047 0.001535

d.CT_VD.CT_VD_TCM -1.4724 0.3701 0.0008276 0.001325

d.CT_VD.CT_VD_TFMox -1.0320 0.6794 0.0015192 0.003321

d.CT_VD.CT_VD_WarmAcu -1.2197 0.5873 0.0013132 0.002128

d.CT_VD.TCM -1.1011 0.5231 0.0011696 0.001637

d.CT_VD.TCM_FNeedle -1.6990 0.5175 0.0011572 0.001632

d.CT_VD.TCM_Mox 1.8110 0.5862 0.0013108 0.002040

d.CT_VD_Acu.CT_VD_BP 0.7296 0.5639 0.0012609 0.001795

d.CT_VD_Acu.CT_VD_DuMox -1.0146 0.4502 0.0010067 0.001884

d.CT_VD_Acu.CT_VD_ShortPri -1.1113 0.6034 0.0013492 0.002290

d.CT_VD_Acu.CT_VD_TCM 1.4983 0.5386 0.0012044 0.001872

d.CT_VD_Acu.CT_VD_TCM_Acu -0.5028 0.5703 0.0012751 0.002166

d.CT_VD_BP.CT_VD_BP_Acu -1.4530 0.4163 0.0009309 0.001555

d.CT_VD_BP.CT_VD_BP_Acu_Pat -2.1414 0.5311 0.0011875 0.001391

d.CT_VD_BP.CT_VD_BP_Pat -1.1891 0.5760 0.0012881 0.002068

d.CT_VD_BP_Acu.CT_VD_BP_Acu_Mox -0.6395 0.5178 0.0011579 0.001252

d.CT_VD_BP_Acu.CT_VD_BP_TCM_Acu -0.8900 0.5507 0.0012315 0.001691

d.DuMox.TCM -1.4894 0.6150 0.0013751 0.002863

d.DuMox.TCM_DuMox -2.5357 0.6151 0.0013753 0.002845

d.TCM.TCM_Acu -0.6907 0.5550 0.0012410 0.001710

d.TCM.TCM_WarmAcu -0.9300 0.5421 0.0012123 0.001527

sd.d 0.4568 0.2166 0.0004843 0.003425

2. Quantiles for each variable:

2.5% 25% 50% 75% 97.5%

d.ACE.E 0.5345 1.42171 1.8306 2.2410 3.12890

d.ACE.TCM -0.7912 0.07996 0.4830 0.8792 1.74930

d.ACE.TCM_ACE -1.8651 -0.98918 -0.5948 -0.1932 0.68764

d.Cel.TCM_Acu -1.9008 -1.13022 -0.8409 -0.5516 0.22890

d.CT_VD.CT_VD_ACE -2.1487 -1.70505 -1.5304 -1.3485 -0.89055

d.CT_VD.CT_VD_Acu -2.1550 -1.69816 -1.5087 -1.3152 -0.84084

d.CT_VD.CT_VD_ChenpiDuMox -4.0913 -3.28490 -2.9225 -2.5560 -1.74305

d.CT_VD.CT_VD_DuMox -3.1351 -2.53353 -2.2692 -2.0074 -1.41032

d.CT_VD.CT_VD_MNKnife_Mox -2.3675 -1.55671 -1.2041 -0.8518 -0.01940

d.CT_VD.CT_VD_Mox -1.3767 -0.80845 -0.5645 -0.3218 0.23993

d.CT_VD.CT_VD_TCM -2.2200 -1.68697 -1.4699 -1.2555 -0.73672

d.CT_VD.CT_VD_TFMox -2.3708 -1.46029 -1.0338 -0.6041 0.30840

d.CT_VD.CT_VD_WarmAcu -2.3939 -1.57428 -1.2219 -0.8668 -0.04055

d.CT_VD.TCM -2.1657 -1.38801 -1.1001 -0.8142 -0.03810

d.CT_VD.TCM_FNeedle -2.7551 -1.98038 -1.7007 -1.4193 -0.64681

d.CT_VD.TCM_Mox 0.6327 1.46037 1.8125 2.1637 2.97933

d.CT_VD_Acu.CT_VD_BP -0.4045 0.39873 0.7313 1.0607 1.86128

d.CT_VD_Acu.CT_VD_DuMox -1.9254 -1.29098 -1.0085 -0.7332 -0.13835

d.CT_VD_Acu.CT_VD_ShortPri -2.3171 -1.47985 -1.1108 -0.7467 0.09431

d.CT_VD_Acu.CT_VD_TCM 0.4063 1.19525 1.4972 1.8055 2.58462

d.CT_VD_Acu.CT_VD_TCM_Acu -1.6447 -0.84059 -0.5025 -0.1645 0.63514

d.CT_VD_BP.CT_VD_BP_Acu -2.3164 -1.69619 -1.4399 -1.1985 -0.65633

d.CT_VD_BP.CT_VD_BP_Acu_Pat -3.2162 -2.43816 -2.1425 -1.8444 -1.06625

d.CT_VD_BP.CT_VD_BP_Pat -2.3427 -1.53386 -1.1882 -0.8469 -0.03955

d.CT_VD_BP_Acu.CT_VD_BP_Acu_Mox -1.6965 -0.92259 -0.6384 -0.3581 0.41457

d.CT_VD_BP_Acu.CT_VD_BP_TCM_Acu -1.9996 -1.20619 -0.8900 -0.5735 0.22125

d.DuMox.TCM -2.7141 -1.86704 -1.4848 -1.1148 -0.26976

d.DuMox.TCM_DuMox -3.7620 -2.91090 -2.5334 -2.1607 -1.31241

d.TCM.TCM_Acu -1.8034 -1.00949 -0.6924 -0.3718 0.43082

d.TCM.TCM_WarmAcu -2.0260 -1.23794 -0.9291 -0.6227 0.16400

sd.d 0.1171 0.31557 0.4280 0.5653 0.94946

-- Model fit (residual deviance):

Dbar pD DIC

71.61765 67.80401 139.42166

70 data points, ratio 1.023, I^2 = 4%

# PINP

## Consistency

Results on the Mean Difference scale

Iterations = 20001:70000

Thinning interval = 1

Number of chains = 4

Sample size per chain = 50000

1. Empirical mean and standard deviation for each variable,

plus standard error of the mean:

Mean SD Naive SE Time-series SE

d.CT_VD.CT_VD_TFMox 0.122216 7.132 0.015947 0.04402

d.CT_VD_Acu.CT_VD 0.008363 5.091 0.011385 0.01141

d.CT_VD_Acu.CT_VD_BP 5.587321 5.355 0.011974 0.01649

d.CT_VD_BP.CT_VD_BP_Acu -8.801398 5.881 0.013149 0.02411

d.CT_VD_BP.CT_VD_BP_Acu_DuMox 7.626453 6.241 0.013956 0.02801

d.CT_VD_BP.CT_VD_BP_AcuInj -7.588657 5.172 0.011566 0.01270

d.CT_VD_BP.CT_VD_BP_Mox -3.480761 6.997 0.015646 0.04256

d.CT_VD_BP.CT_VD_BP_Pat 5.092947 5.262 0.011767 0.01348

d.CT_VD_BP.CT_VD_BP_TCM_ACE -5.689683 5.270 0.011785 0.01421

d.CT_VD_BP.CT_VD_BP_TCM_Acu_Mox -7.542480 5.963 0.013334 0.02496

d.CT_VD_BP_Acu.CT_VD_BP_Acu_Mox 5.896029 5.133 0.011477 0.01211

sd.d 4.403391 2.531 0.005659 0.03642

2. Quantiles for each variable:

2.5% 25% 50% 75% 97.5%

d.CT_VD.CT_VD_TFMox -14.1871 -4.389 0.08715 4.6519 14.396

d.CT_VD_Acu.CT_VD -11.0731 -2.293 0.01162 2.3174 11.092

d.CT_VD_Acu.CT_VD_BP -5.7784 2.806 5.58327 8.3590 17.010

d.CT_VD_BP.CT_VD_BP_Acu -21.0096 -12.188 -8.78790 -5.4140 3.404

d.CT_VD_BP.CT_VD_BP_Acu_DuMox -5.0874 3.885 7.58989 11.3214 20.475

d.CT_VD_BP.CT_VD_BP_AcuInj -18.7311 -10.070 -7.58799 -5.1498 3.642

d.CT_VD_BP.CT_VD_BP_Mox -17.4887 -7.861 -3.49018 0.8927 10.680

d.CT_VD_BP.CT_VD_BP_Pat -6.2034 2.492 5.09911 7.6909 16.420

d.CT_VD_BP.CT_VD_BP_TCM_ACE -16.9862 -8.322 -5.69973 -3.0582 5.649

d.CT_VD_BP.CT_VD_BP_TCM_Acu_Mox -19.8208 -11.014 -7.56561 -4.0543 4.799

d.CT_VD_BP_Acu.CT_VD_BP_Acu_Mox -5.2022 3.513 5.90032 8.2911 17.010

sd.d 0.2558 2.212 4.40252 6.5896 8.587

-- Model fit (residual deviance):

Dbar pD DIC

21.96925 21.96859 43.93785

22 data points, ratio 0.9986, I^2 = 4%

## Inconsistency

Results on the Mean Difference scale

Iterations = 20001:70000

Thinning interval = 1

Number of chains = 4

Sample size per chain = 50000

1. Empirical mean and standard deviation for each variable,

plus standard error of the mean:

Mean SD Naive SE Time-series SE

d.CT_VD.CT_VD_Acu -0.03749 5.054 0.011301 0.01125

d.CT_VD.CT_VD_TFMox 0.19768 7.069 0.015807 0.04371

d.CT_VD_Acu.CT_VD_BP 5.58872 5.329 0.011916 0.01653

d.CT_VD_BP.CT_VD_BP_Acu -8.80828 5.833 0.013042 0.02418

d.CT_VD_BP.CT_VD_BP_Acu_DuMox 7.77117 6.223 0.013915 0.02994

d.CT_VD_BP.CT_VD_BP_AcuInj -7.60571 5.129 0.011468 0.01259

d.CT_VD_BP.CT_VD_BP_Mox -3.50217 7.036 0.015733 0.04612

d.CT_VD_BP.CT_VD_BP_Pat 5.09962 5.242 0.011720 0.01375

d.CT_VD_BP.CT_VD_BP_TCM_ACE -5.68393 5.245 0.011728 0.01391

d.CT_VD_BP.CT_VD_BP_TCM_Acu_Mox -7.56977 5.961 0.013330 0.02654

d.CT_VD_BP_Acu.CT_VD_BP_Acu_Mox 5.88882 5.084 0.011369 0.01210

sd.d 4.35371 2.544 0.005689 0.03750

2. Quantiles for each variable:

2.5% 25% 50% 75% 97.5%

d.CT_VD.CT_VD_Acu -11.1257 -2.298 -0.01805 2.2336 10.997

d.CT_VD.CT_VD_TFMox -13.9959 -4.272 0.21221 4.6580 14.360

d.CT_VD_Acu.CT_VD_BP -5.8298 2.845 5.61381 8.3331 16.949

d.CT_VD_BP.CT_VD_BP_Acu -20.9337 -12.140 -8.82089 -5.4396 3.267

d.CT_VD_BP.CT_VD_BP_Acu_DuMox -4.9412 4.033 7.77522 11.5179 20.479

d.CT_VD_BP.CT_VD_BP_AcuInj -18.6929 -10.035 -7.61964 -5.1663 3.413

d.CT_VD_BP.CT_VD_BP_Mox -17.6559 -7.921 -3.51192 0.9428 10.615

d.CT_VD_BP.CT_VD_BP_Pat -6.2496 2.543 5.11560 7.6851 16.305

d.CT_VD_BP.CT_VD_BP_TCM_ACE -17.0094 -8.255 -5.70660 -3.0854 5.571

d.CT_VD_BP.CT_VD_BP_TCM_Acu_Mox -19.8130 -11.039 -7.57926 -4.0940 4.754

d.CT_VD_BP_Acu.CT_VD_BP_Acu_Mox -5.2261 3.556 5.90981 8.2411 16.890

sd.d 0.1939 2.166 4.33220 6.5452 8.583

-- Model fit (residual deviance):

Dbar pD DIC

21.99622 21.99575 43.99197

22 data points, ratio 0.9998, I^2 = 5%

# CTX

## Consistency

Results on the Mean Difference scale

Iterations = 20001:70000

Thinning interval = 1

Number of chains = 4

Sample size per chain = 50000

1. Empirical mean and standard deviation for each variable,

plus standard error of the mean:

Mean SD Naive SE Time-series SE

d.CT_VD.CT_VD_TFMox -0.070823 0.08180 0.0001829 0.0002811

d.CT_VD_Acu.CT_VD 0.029771 0.14442 0.0003229 0.0050600

d.CT_VD_Acu.CT_VD_BP 0.140273 0.11671 0.0002610 0.0004076

d.CT_VD_Acu.CT_VD_TCM -0.019909 0.11741 0.0002625 0.0005784

d.CT_VD_Acu.CT_VD_TCM_Acu -0.170442 0.11612 0.0002596 0.0005474

d.CT_VD_BP.CT_VD_BP_Acu -0.074981 0.06595 0.0001475 0.0002150

d.CT_VD_BP.CT_VD_BP_Acu_DuMox -0.030164 0.11099 0.0002482 0.0002997

d.CT_VD_BP.CT_VD_BP_Acu_Pat -0.150032 0.11836 0.0002647 0.0004915

d.CT_VD_BP.CT_VD_BP_AcuInj -0.109936 0.11021 0.0002464 0.0002448

d.CT_VD_BP.CT_VD_BP_Mox -0.060424 0.12179 0.0002723 0.0006328

d.CT_VD_BP.CT_VD_BP_Pat 0.009997 0.11120 0.0002486 0.0002711

d.CT_VD_BP.CT_VD_BP_TCM_Acu_Mox -0.059956 0.10900 0.0002437 0.0002454

d.CT_VD_BP_Acu.CT_VD_BP_Acu_Mox -0.090169 0.11120 0.0002486 0.0002874

d.CT_VD_BP_Acu.CT_VD_BP_TCM_Acu -0.140018 0.11865 0.0002653 0.0005459

d.CT_VD_BP_Acu.CT_VD_BP_TFMox -0.070670 0.11312 0.0002529 0.0002880

sd.d 0.053678 0.09631 0.0002153 0.0060527

2. Quantiles for each variable:

2.5% 25% 50% 75% 97.5%

d.CT_VD.CT_VD_TFMox -0.1842103 -0.093764 -0.072035 -0.049601 0.050855

d.CT_VD_Acu.CT_VD -0.0981806 -0.002561 0.010456 0.029840 0.271412

d.CT_VD_Acu.CT_VD_BP -0.0248319 0.107843 0.139908 0.172073 0.307026

d.CT_VD_Acu.CT_VD_TCM -0.1872730 -0.055480 -0.020421 0.015712 0.150128

d.CT_VD_Acu.CT_VD_TCM_Acu -0.3372887 -0.204819 -0.170441 -0.136318 -0.003188

d.CT_VD_BP.CT_VD_BP_Acu -0.1771717 -0.090343 -0.073325 -0.057971 0.019560

d.CT_VD_BP.CT_VD_BP_Acu_DuMox -0.1895738 -0.051669 -0.030216 -0.008188 0.128941

d.CT_VD_BP.CT_VD_BP_Acu_Pat -0.3207548 -0.188897 -0.149930 -0.111644 0.023003

d.CT_VD_BP.CT_VD_BP_AcuInj -0.2670068 -0.128259 -0.109593 -0.091281 0.047505

d.CT_VD_BP.CT_VD_BP_Mox -0.2342361 -0.103682 -0.061215 -0.017411 0.117304

d.CT_VD_BP.CT_VD_BP_Pat -0.1491624 -0.011443 0.009946 0.031081 0.168381

d.CT_VD_BP.CT_VD_BP_TCM_Acu_Mox -0.2177726 -0.077673 -0.060081 -0.042500 0.097103

d.CT_VD_BP_Acu.CT_VD_BP_Acu_Mox -0.2480298 -0.112864 -0.090021 -0.067165 0.068596

d.CT_VD_BP_Acu.CT_VD_BP_TCM_Acu -0.3144977 -0.178748 -0.140062 -0.100226 0.032547

d.CT_VD_BP_Acu.CT_VD_BP_TFMox -0.2307422 -0.094786 -0.070363 -0.045450 0.087092

sd.d 0.0009811 0.011784 0.027455 0.055379 0.305507

-- Model fit (residual deviance):

Dbar pD DIC

48.16403 34.27680 82.44083

37 data points, ratio 1.302, I^2 = 25%

## Inconsistency

Results on the Mean Difference scale

Iterations = 20001:70000

Thinning interval = 1

Number of chains = 4

Sample size per chain = 50000

1. Empirical mean and standard deviation for each variable,

plus standard error of the mean:

Mean SD Naive SE Time-series SE

d.CT_VD.CT_VD_Acu -0.07955 0.3680 0.0008228 0.0255809

d.CT_VD.CT_VD_TFMox -0.07156 0.1766 0.0003949 0.0004312

d.CT_VD_Acu.CT_VD_BP 0.13898 0.2501 0.0005593 0.0006793

d.CT_VD_Acu.CT_VD_TCM -0.02136 0.2527 0.0005650 0.0009050

d.CT_VD_Acu.CT_VD_TCM_Acu -0.17158 0.2499 0.0005589 0.0009427

d.CT_VD_BP.CT_VD_BP_Acu -0.07498 0.1463 0.0003271 0.0003687

d.CT_VD_BP.CT_VD_BP_Acu_DuMox -0.03028 0.2472 0.0005527 0.0005055

d.CT_VD_BP.CT_VD_BP_Acu_Pat -0.15064 0.2541 0.0005681 0.0007385

d.CT_VD_BP.CT_VD_BP_AcuInj -0.11017 0.2465 0.0005512 0.0004894

d.CT_VD_BP.CT_VD_BP_Mox -0.06019 0.2511 0.0005614 0.0007647

d.CT_VD_BP.CT_VD_BP_Pat 0.01019 0.2493 0.0005575 0.0006107

d.CT_VD_BP.CT_VD_BP_TCM_Acu_Mox -0.06044 0.2522 0.0005640 0.0005616

d.CT_VD_BP_Acu.CT_VD_BP_Acu_Mox -0.09028 0.2501 0.0005593 0.0006016

d.CT_VD_BP_Acu.CT_VD_BP_TCM_Acu -0.13984 0.2537 0.0005672 0.0007110

d.CT_VD_BP_Acu.CT_VD_BP_TFMox -0.07006 0.2509 0.0005610 0.0005426

sd.d 0.09106 0.2312 0.0005171 0.0379681

2. Quantiles for each variable:

2.5% 25% 50% 75% 97.5%

d.CT_VD.CT_VD_Acu -1.093371 -0.03301 -0.01100 0.002151 0.09886

d.CT_VD.CT_VD_TFMox -0.260456 -0.09507 -0.07239 -0.048822 0.12068

d.CT_VD_Acu.CT_VD_BP -0.131655 0.10496 0.13927 0.174081 0.40757

d.CT_VD_Acu.CT_VD_TCM -0.294128 -0.05776 -0.02215 0.015360 0.25551

d.CT_VD_Acu.CT_VD_TCM_Acu -0.442757 -0.20721 -0.17151 -0.135778 0.09985

d.CT_VD_BP.CT_VD_BP_Acu -0.236171 -0.09114 -0.07351 -0.057533 0.07987

d.CT_VD_BP.CT_VD_BP_Acu_DuMox -0.298502 -0.05302 -0.03037 -0.007527 0.23974

d.CT_VD_BP.CT_VD_BP_Acu_Pat -0.419491 -0.19134 -0.15110 -0.110675 0.12999

d.CT_VD_BP.CT_VD_BP_AcuInj -0.380284 -0.12951 -0.10996 -0.090766 0.15695

d.CT_VD_BP.CT_VD_BP_Mox -0.341255 -0.10425 -0.05934 -0.015245 0.21243

d.CT_VD_BP.CT_VD_BP_Pat -0.262139 -0.01189 0.01068 0.032548 0.27837

d.CT_VD_BP.CT_VD_BP_TCM_Acu_Mox -0.333615 -0.07855 -0.05996 -0.041543 0.20902

d.CT_VD_BP_Acu.CT_VD_BP_Acu_Mox -0.357569 -0.11403 -0.09028 -0.066156 0.17736

d.CT_VD_BP_Acu.CT_VD_BP_TCM_Acu -0.419744 -0.18245 -0.14003 -0.097358 0.13618

d.CT_VD_BP_Acu.CT_VD_BP_TFMox -0.334888 -0.09549 -0.07041 -0.044510 0.19738

sd.d 0.000914 0.01236 0.02831 0.059315 0.93602

-- Model fit (residual deviance):

Dbar pD DIC

48.03249 34.56238 82.59487

37 data points, ratio 1.298, I^2 = 25%

# E2

## Consistency

Results on the Mean Difference scale

Iterations = 20001:70000

Thinning interval = 1

Number of chains = 4

Sample size per chain = 50000

1. Empirical mean and standard deviation for each variable,

plus standard error of the mean:

Mean SD Naive SE Time-series SE

d.CT_VD.CT_VD_ACE 9.2605 2.485 0.005556 0.009315

d.CT_VD.CT_VD_TCM 4.5262 2.919 0.006526 0.010815

d.CT_VD.CT_VD_WarmAcu 4.2881 4.191 0.009372 0.011106

d.CT_VD_Acu.CT_VD -19.3694 4.727 0.010570 0.021205

d.CT_VD_Acu.CT_VD_BP -1.2017 4.066 0.009093 0.009171

d.CT_VD_BP.CT_VD_BP_Acu 1.7793 4.126 0.009225 0.010737

d.CT_VD_BP.CT_VD_BP_Mox 0.8482 4.193 0.009376 0.012111

d.CT_VD_BP.CT_VD_BP_Pat 1.8225 4.063 0.009084 0.009383

d.CT_VD_BP.CT_VD_BP_TCM_ACE 3.9486 4.055 0.009068 0.009322

sd.d 2.9508 2.783 0.006223 0.047271

2. Quantiles for each variable:

2.5% 25% 50% 75% 97.5%

d.CT_VD.CT_VD_ACE 4.1645 8.2463 9.2965 10.3241 14.088

d.CT_VD.CT_VD_TCM -1.2599 3.2901 4.5210 5.7722 10.322

d.CT_VD.CT_VD_WarmAcu -4.0684 2.7009 4.2698 5.8794 12.718

d.CT_VD_Acu.CT_VD -28.5523 -21.7441 -19.3671 -17.0015 -10.036

d.CT_VD_Acu.CT_VD_BP -9.4617 -2.5220 -1.2078 0.1342 7.068

d.CT_VD_BP.CT_VD_BP_Acu -6.5088 0.3115 1.7696 3.2420 10.144

d.CT_VD_BP.CT_VD_BP_Mox -7.4905 -0.7952 0.8406 2.4921 9.260

d.CT_VD_BP.CT_VD_BP_Pat -6.4162 0.5015 1.8265 3.1443 9.989

d.CT_VD_BP.CT_VD_BP_TCM_ACE -4.3595 2.6177 3.9496 5.2692 12.310

sd.d 0.1354 1.1963 2.1658 3.7264 11.193

-- Model fit (residual deviance):

Dbar pD DIC

22.26228 21.41314 43.67542

22 data points, ratio 1.012, I^2 = 6%

## Inconsistency

Results on the Mean Difference scale

Iterations = 20001:70000

Thinning interval = 1

Number of chains = 4

Sample size per chain = 50000

1. Empirical mean and standard deviation for each variable,

plus standard error of the mean:

Mean SD Naive SE Time-series SE

d.CT_VD.CT_VD_ACE 9.2660 2.535 0.005668 0.009565

d.CT_VD.CT_VD_Acu 19.3592 4.809 0.010753 0.020434

d.CT_VD.CT_VD_TCM 4.5250 2.986 0.006678 0.011404

d.CT_VD.CT_VD_WarmAcu 4.2592 4.293 0.009600 0.011103

d.CT_VD_Acu.CT_VD_BP -1.2054 4.218 0.009432 0.009444

d.CT_VD_BP.CT_VD_BP_Acu 1.7693 4.248 0.009499 0.010033

d.CT_VD_BP.CT_VD_BP_Mox 0.8521 4.320 0.009660 0.012752

d.CT_VD_BP.CT_VD_BP_Pat 1.8299 4.189 0.009366 0.009386

d.CT_VD_BP.CT_VD_BP_TCM_ACE 3.9552 4.186 0.009361 0.009775

sd.d 3.0049 2.882 0.006443 0.051323

2. Quantiles for each variable:

2.5% 25% 50% 75% 97.5%

d.CT_VD.CT_VD_ACE 4.1087 8.2415 9.2959 10.3227 14.275

d.CT_VD.CT_VD_Acu 9.9458 16.9917 19.3776 21.7110 28.690

d.CT_VD.CT_VD_TCM -1.4441 3.2821 4.5155 5.7808 10.489

d.CT_VD.CT_VD_WarmAcu -4.4395 2.6796 4.2524 5.8428 12.895

d.CT_VD_Acu.CT_VD_BP -9.7312 -2.5526 -1.1987 0.1517 7.263

d.CT_VD_BP.CT_VD_BP_Acu -6.8143 0.3129 1.7629 3.2399 10.352

d.CT_VD_BP.CT_VD_BP_Mox -7.8560 -0.8104 0.8534 2.4914 9.667

d.CT_VD_BP.CT_VD_BP_Pat -6.6291 0.4856 1.8223 3.1525 10.317

d.CT_VD_BP.CT_VD_BP_TCM_ACE -4.5295 2.6341 3.9562 5.2931 12.430

sd.d 0.1353 1.1853 2.1824 3.7607 11.683

-- Model fit (residual deviance):

Dbar pD DIC

22.23106 21.37255 43.60361

22 data points, ratio 1.011, I^2 = 6%

# ALP (exclude BP)

## Consistency

Results on the Mean Difference scale

Iterations = 20001:70000

Thinning interval = 1

Number of chains = 4

Sample size per chain = 50000

1. Empirical mean and standard deviation for each variable,

plus standard error of the mean:

Mean SD Naive SE Time-series SE

d.CT_VD.CT_VD_Mox 13.215 8.513 0.019035 0.02785

d.CT_VD.CT_VD_TCM 12.845 8.939 0.019988 0.03073

d.CT_VD.CT_VD_TCM_Acu 7.283 8.335 0.018637 0.02052

d.CT_VD.CT_VD_TCM_Mox -0.904 8.575 0.019175 0.02280

d.TCM.ACE 5.206 9.865 0.022060 0.05201

d.TCM.E 2.547 9.604 0.021474 0.04208

d.TCM.TCM_ACE 11.275 9.884 0.022101 0.04485

d.TCM_Acu.Acu -5.049 8.230 0.018402 0.02711

d.TCM_Acu.CT_VD 12.555 8.276 0.018505 0.01940

d.TCM_Acu.TCM -5.741 6.067 0.013566 0.01808

sd.d 7.532 3.203 0.007162 0.02971

2. Quantiles for each variable:

2.5% 25% 50% 75% 97.5%

d.CT_VD.CT_VD_Mox -4.491 8.3925 13.2055 18.0307 30.999

d.CT_VD.CT_VD_TCM -5.584 7.6031 12.8424 18.1373 31.215

d.CT_VD.CT_VD_TCM_Acu -10.251 2.6720 7.2807 11.9074 24.625

d.CT_VD.CT_VD_TCM_Mox -18.733 -5.7671 -0.8929 3.9418 16.871

d.TCM.ACE -14.809 -0.9048 5.2106 11.3075 25.091

d.TCM.E -16.961 -3.3252 2.5389 8.4620 22.032

d.TCM.TCM_ACE -8.765 5.1714 11.2583 17.3840 31.158

d.TCM_Acu.Acu -21.678 -9.9019 -5.2478 -0.2853 12.296

d.TCM_Acu.CT_VD -4.701 8.0210 12.5674 17.0907 29.886

d.TCM_Acu.TCM -17.842 -9.2887 -6.0731 -2.3023 7.313

sd.d 1.678 4.9957 7.5130 10.1737 12.959

-- Model fit (residual deviance):

Dbar pD DIC

18.26444 18.05470 36.31914

18 data points, ratio 1.015, I^2 = 7%

## Inconsistency

Results on the Mean Difference scale

Iterations = 20001:70000

Thinning interval = 1

Number of chains = 4

Sample size per chain = 50000

1. Empirical mean and standard deviation for each variable,

plus standard error of the mean:

Mean SD Naive SE Time-series SE

d.ACE.E -2.6130 9.245 0.020674 0.05671

d.ACE.TCM -5.1944 9.399 0.021017 0.05990

d.ACE.TCM_ACE 6.0680 9.528 0.021306 0.05999

d.Acu.TCM 1.9317 8.263 0.018478 0.03131

d.Acu.TCM_Acu 2.0333 8.410 0.018805 0.03401

d.CT_VD.CT_VD_Mox 13.2387 8.055 0.018011 0.02784

d.CT_VD.CT_VD_TCM 12.9058 8.478 0.018957 0.03333

d.CT_VD.CT_VD_TCM_Acu 7.2697 7.868 0.017592 0.01989

d.CT_VD.CT_VD_TCM_Mox -0.9361 8.056 0.018013 0.02256

d.CT_VD.TCM_Acu -12.6144 7.789 0.017417 0.01856

d.TCM.TCM_Acu 9.8468 7.683 0.017179 0.01753

sd.d 6.6263 3.839 0.008584 0.05833

2. Quantiles for each variable:

2.5% 25% 50% 75% 97.5%

d.ACE.E -21.6707 -8.0894 -2.6029 2.8273 16.470

d.ACE.TCM -24.3711 -10.8226 -5.1440 0.4412 13.979

d.ACE.TCM_ACE -13.2845 0.3208 5.9955 11.8383 25.558

d.Acu.TCM -15.6225 -2.4709 1.9128 6.3312 19.429

d.Acu.TCM_Acu -15.6419 -2.5507 2.0615 6.6203 19.744

d.CT_VD.CT_VD_Mox -4.0450 9.1808 13.2400 17.3386 30.459

d.CT_VD.CT_VD_TCM -4.9207 8.2348 12.8945 17.5856 30.650

d.CT_VD.CT_VD_TCM_Acu -9.6984 3.4947 7.2601 11.0668 24.226

d.CT_VD.CT_VD_TCM_Mox -18.1086 -5.0244 -0.9904 3.1730 16.420

d.CT_VD.TCM_Acu -29.5200 -16.2324 -12.6056 -8.9777 4.215

d.TCM.TCM_Acu -6.9907 6.3622 9.8326 13.3505 26.563

sd.d 0.3034 3.3209 6.6268 9.9430 12.955

-- Model fit (residual deviance):

Dbar pD DIC

17.99087 17.99021 35.98108

18 data points, ratio 0.9995, I^2 = 6%

# ALP (include BP)

## Consistency

Results on the Mean Difference scale

Iterations = 20001:70000

Thinning interval = 1

Number of chains = 4

Sample size per chain = 50000

1. Empirical mean and standard deviation for each variable,

plus standard error of the mean:

Mean SD Naive SE Time-series SE

d.CT_VD_BP.CT_VD_BP_Acu -12.918 4.812 0.01076 0.01140

d.CT_VD_BP.CT_VD_BP_Acu_Pat -12.395 6.959 0.01556 0.02017

d.CT_VD_BP.CT_VD_BP_IMox -3.219 7.557 0.01690 0.03490

d.CT_VD_BP.CT_VD_BP_Mox -5.797 8.621 0.01928 0.06104

d.CT_VD_BP.CT_VD_BP_TCM_ACE -17.593 7.711 0.01724 0.04029

d.CT_VD_BP.CT_VD_BP_TCM_Acu_Mox -5.913 7.626 0.01705 0.03618

d.CT_VD_BP_Acu.CT_VD_BP_TFMox 5.078 6.958 0.01556 0.01947

sd.d 4.931 4.634 0.01036 0.07574

2. Quantiles for each variable:

2.5% 25% 50% 75% 97.5%

d.CT_VD_BP.CT_VD_BP_Acu -23.86571 -14.367 -12.863 -11.4543 -2.067

d.CT_VD_BP.CT_VD_BP_Acu_Pat -27.94741 -14.896 -12.394 -9.8930 3.164

d.CT_VD_BP.CT_VD_BP_IMox -19.33670 -6.835 -3.226 0.3653 13.096

d.CT_VD_BP.CT_VD_BP_Mox -23.62066 -10.539 -5.801 -0.9380 11.711

d.CT_VD_BP.CT_VD_BP_TCM_ACE -34.02238 -21.410 -17.625 -13.7700 -1.093

d.CT_VD_BP.CT_VD_BP_TCM_Acu_Mox -22.36599 -9.558 -5.911 -2.2611 10.502

d.CT_VD_BP_Acu.CT_VD_BP_TFMox -10.52137 2.592 5.075 7.5309 20.647

sd.d 0.09573 1.199 3.247 7.6394 16.185

-- Model fit (residual deviance):

Dbar pD DIC

15.81543 15.77341 31.58884

16 data points, ratio 0.9885, I^2 = 5%

## Inconsistency

Results on the Mean Difference scale

Iterations = 20001:70000

Thinning interval = 1

Number of chains = 4

Sample size per chain = 50000

1. Empirical mean and standard deviation for each variable,

plus standard error of the mean:

Mean SD Naive SE Time-series SE

d.CT_VD_BP.CT_VD_BP_Acu -12.892 4.876 0.01090 0.01170

d.CT_VD_BP.CT_VD_BP_Acu_Pat -12.387 7.068 0.01581 0.01987

d.CT_VD_BP.CT_VD_BP_IMox -3.242 7.632 0.01707 0.03442

d.CT_VD_BP.CT_VD_BP_Mox -5.931 8.674 0.01940 0.06027

d.CT_VD_BP.CT_VD_BP_TCM_ACE -17.674 7.769 0.01737 0.03880

d.CT_VD_BP.CT_VD_BP_TCM_Acu_Mox -5.905 7.650 0.01711 0.03640

d.CT_VD_BP_Acu.CT_VD_BP_TFMox 5.099 7.011 0.01568 0.01937

sd.d 4.991 4.652 0.01040 0.07392

2. Quantiles for each variable:

2.5% 25% 50% 75% 97.5%

d.CT_VD_BP.CT_VD_BP_Acu -23.9346 -14.378 -12.849 -11.4223 -1.847

d.CT_VD_BP.CT_VD_BP_Acu_Pat -28.2212 -14.952 -12.409 -9.8695 3.525

d.CT_VD_BP.CT_VD_BP_IMox -19.5223 -6.883 -3.256 0.3406 13.154

d.CT_VD_BP.CT_VD_BP_Mox -23.8218 -10.803 -5.977 -1.0580 11.827

d.CT_VD_BP.CT_VD_BP_TCM_ACE -34.3721 -21.404 -17.660 -13.8459 -1.247

d.CT_VD_BP.CT_VD_BP_TCM_Acu_Mox -22.3314 -9.589 -5.893 -2.2738 10.476

d.CT_VD_BP_Acu.CT_VD_BP_TFMox -10.7207 2.583 5.121 7.6098 20.755

sd.d 0.1094 1.233 3.324 7.7474 16.191

-- Model fit (residual deviance):

Dbar pD DIC

15.81531 15.77645 31.59176

16 data points, ratio 0.9885, I^2 = 5%

# OCN (exclude BP)

## Consistency

Results on the Mean Difference scale

Iterations = 20001:70000

Thinning interval = 1

Number of chains = 4

Sample size per chain = 50000

1. Empirical mean and standard deviation for each variable,

plus standard error of the mean:

Mean SD Naive SE Time-series SE

d.CT_VD.CT_VD_Acu -2.8505 1.7540 0.003922 0.004503

d.CT_VD.CT_VD_HSMox_ElecAcu -1.1171 1.7265 0.003861 0.004197

d.CT_VD.CT_VD_Mox 0.9251 1.6980 0.003797 0.005019

d.CT_VD.CT_VD_TCM 0.3651 1.6960 0.003792 0.005020

d.CT_VD.CT_VD_TCM_Acu 0.7137 1.1982 0.002679 0.002779

d.CT_VD.CT_VD_TFMox 0.2090 1.6832 0.003764 0.003747

d.CT_VD.CT_VD_WarmAcu -2.0953 1.7239 0.003855 0.004312

sd.d 1.5465 0.6668 0.001491 0.004830

2. Quantiles for each variable:

2.5% 25% 50% 75% 97.5%

d.CT_VD.CT_VD_Acu -6.524 -3.832370 -2.8496 -1.8704 0.803

d.CT_VD.CT_VD_HSMox_ElecAcu -4.730 -2.059375 -1.1202 -0.1736 2.518

d.CT_VD.CT_VD_Mox -2.645 0.003019 0.9250 1.8417 4.511

d.CT_VD.CT_VD_TCM -3.214 -0.556645 0.3669 1.2791 3.943

d.CT_VD.CT_VD_TCM_Acu -1.778 0.063238 0.6930 1.3649 3.254

d.CT_VD.CT_VD_TFMox -3.357 -0.689842 0.2098 1.1063 3.752

d.CT_VD.CT_VD_WarmAcu -5.696 -3.050140 -2.0968 -1.1468 1.520

sd.d 0.467 0.993249 1.4883 2.0868 2.763

-- Model fit (residual deviance):

Dbar pD DIC

15.05008 15.01041 30.06048

15 data points, ratio 1.003, I^2 = 7%

## Inconsistency

Results on the Mean Difference scale

Iterations = 20001:70000

Thinning interval = 1

Number of chains = 4

Sample size per chain = 50000

1. Empirical mean and standard deviation for each variable,

plus standard error of the mean:

Mean SD Naive SE Time-series SE

d.CT_VD.CT_VD_Acu -2.8487 1.7403 0.003891 0.004558

d.CT_VD.CT_VD_HSMox_ElecAcu -1.1223 1.7130 0.003830 0.004145

d.CT_VD.CT_VD_Mox 0.9131 1.6846 0.003767 0.004989

d.CT_VD.CT_VD_TCM 0.3623 1.6875 0.003773 0.005020

d.CT_VD.CT_VD_TCM_Acu 0.7131 1.1992 0.002681 0.002750

d.CT_VD.CT_VD_TFMox 0.2128 1.6802 0.003757 0.003730

d.CT_VD.CT_VD_WarmAcu -2.0932 1.7163 0.003838 0.004262

sd.d 1.5388 0.6671 0.001492 0.004945

2. Quantiles for each variable:

2.5% 25% 50% 75% 97.5%

d.CT_VD.CT_VD_Acu -6.4813 -3.826228 -2.8496 -1.879 0.796

d.CT_VD.CT_VD_HSMox_ElecAcu -4.7153 -2.060142 -1.1241 -0.184 2.479

d.CT_VD.CT_VD_Mox -2.6352 0.002378 0.9172 1.827 4.453

d.CT_VD.CT_VD_TCM -3.1809 -0.548797 0.3587 1.270 3.945

d.CT_VD.CT_VD_TCM_Acu -1.8096 0.069045 0.6960 1.360 3.252

d.CT_VD.CT_VD_TFMox -3.3305 -0.683476 0.2086 1.103 3.774

d.CT_VD.CT_VD_WarmAcu -5.6929 -3.039205 -2.0955 -1.145 1.504

sd.d 0.4671 0.982750 1.4820 2.077 2.761

-- Model fit (residual deviance):

Dbar pD DIC

15.03987 15.00036 30.04023

15 data points, ratio 1.003, I^2 = 7%

# OCN (include BP)

## Consistency

Results on the Mean Difference scale

Iterations = 20001:70000

Thinning interval = 1

Number of chains = 4

Sample size per chain = 50000

1. Empirical mean and standard deviation for each variable,

plus standard error of the mean:

Mean SD Naive SE Time-series SE

d.CT_VD_BP.CT_VD_BP_Acu 2.550099 1.4764 0.003301 0.003537

d.CT_VD_BP.CT_VD_BP_Acu_Pat 1.193192 1.4793 0.003308 0.003468

d.CT_VD_BP.CT_VD_BP_DuMox 0.002187 1.4498 0.003242 0.003228

d.CT_VD_BP.CT_VD_BP_Mox -0.748874 2.3607 0.005279 0.018553

d.CT_VD_BP.CT_VD_BP_TCM_Acu_Mox 1.887082 1.4551 0.003254 0.003293

d.CT_VD_BP_Acu.CT_VD_BP_Acu_Mox 0.704199 1.4791 0.003307 0.003498

d.CT_VD_BP_Acu.CT_VD_BP_TFMox 0.272720 1.4580 0.003260 0.003259

sd.d 1.253462 0.7362 0.001646 0.008884

2. Quantiles for each variable:

2.5% 25% 50% 75% 97.5%

d.CT_VD_BP.CT_VD_BP_Acu -0.65186 1.85955 2.5524522 3.2467 5.743

d.CT_VD_BP.CT_VD_BP_Acu_Pat -2.02693 0.50517 1.1922186 1.8838 4.382

d.CT_VD_BP.CT_VD_BP_DuMox -3.15463 -0.64452 -0.0001647 0.6481 3.171

d.CT_VD_BP.CT_VD_BP_Mox -5.43967 -2.28027 -0.7434409 0.7809 3.928

d.CT_VD_BP.CT_VD_BP_TCM_Acu_Mox -1.28568 1.23747 1.8888852 2.5369 5.054

d.CT_VD_BP_Acu.CT_VD_BP_Acu_Mox -2.50470 0.01647 0.7036183 1.3935 3.920

d.CT_VD_BP_Acu.CT_VD_BP_TFMox -2.89859 -0.37903 0.2701533 0.9213 3.447

sd.d 0.06388 0.61088 1.2450839 1.8862 2.484

-- Model fit (residual deviance):

Dbar pD DIC

14.00954 14.00937 28.01891

14 data points, ratio 1.001, I^2 = 7%

## Inconsistency

Results on the Mean Difference scale

Iterations = 20001:70000

Thinning interval = 1

Number of chains = 4

Sample size per chain = 50000

1. Empirical mean and standard deviation for each variable,

plus standard error of the mean:

Mean SD Naive SE Time-series SE

d.CT_VD_BP.CT_VD_BP_Acu 2.5461328 1.507 0.003369 0.003604

d.CT_VD_BP.CT_VD_BP_Acu_Pat 1.1845729 1.502 0.003358 0.003561

d.CT_VD_BP.CT_VD_BP_DuMox 0.0001278 1.473 0.003294 0.003285

d.CT_VD_BP.CT_VD_BP_Mox -0.7709871 2.361 0.005280 0.017351

d.CT_VD_BP.CT_VD_BP_TCM_Acu_Mox 1.8882766 1.478 0.003305 0.003272

d.CT_VD_BP_Acu.CT_VD_BP_Acu_Mox 0.7083028 1.497 0.003348 0.003599

d.CT_VD_BP_Acu.CT_VD_BP_TFMox 0.2704451 1.481 0.003312 0.003315

sd.d 1.2843723 0.735 0.001644 0.008667

2. Quantiles for each variable:

2.5% 25% 50% 75% 97.5%

d.CT_VD_BP.CT_VD_BP_Acu -0.71305 1.829609 2.5454374 3.2617 5.805

d.CT_VD_BP.CT_VD_BP_Acu_Pat -2.06816 0.475106 1.1885162 1.8934 4.436

d.CT_VD_BP.CT_VD_BP_DuMox -3.21765 -0.667550 0.0003121 0.6726 3.195

d.CT_VD_BP.CT_VD_BP_Mox -5.46195 -2.313054 -0.7583439 0.7663 3.890

d.CT_VD_BP.CT_VD_BP_TCM_Acu_Mox -1.32090 1.215213 1.8907558 2.5628 5.099

d.CT_VD_BP_Acu.CT_VD_BP_Acu_Mox -2.53027 -0.002091 0.7144940 1.4157 3.946

d.CT_VD_BP_Acu.CT_VD_BP_TFMox -2.95437 -0.402300 0.2707001 0.9434 3.487

sd.d 0.06601 0.649788 1.2848220 1.9237 2.487

-- Model fit (residual deviance):

Dbar pD DIC

14.02003 14.01983 28.03987

14 data points, ratio 1.001, I^2 = 7%
