# Supplementary material for: Evaluating combined acupuncture and antiresorptive therapy in Chinese women with postmenopausal osteoporosis: a systematic review and network meta-analysis
Source: Front Endocrinol (Lausanne). 2026 Jul 1;17:1784394. doi: 10.3389/fendo.2026.1784394 (PMC13368563; doi:10.3389/fendo.2026.1784394)
Supplement: Supplementary file 5 [file DataSheet5.docx]

# LS-BMD

Treatments used

A (reference): CT_VD

B: CT_VD_ACE

C: CT_VD_Acu

D: CT_VD_Acu_GinMox

E: CT_VD_BP

F: CT_VD_BP_Acu

G: CT_VD_BP_Mox

H: CT_VD_BP_TCM_Acu

I: CT_VD_BP_TCM_WarmAcu

J: CT_VD_MNKnife_Mox

K: CT_VD_Mox

L: CT_VD_ShortPri

M: CT_VD_TCM

N: CT_VD_TCM_Acu

O: CT_VD_TCM_FDMox

P: CT_VD_TCM_Mox

Q: CT_VD_TFMox

R: Cal

S: Cal_ElecAcu

T: ElecAcu

U: TCM_Mox

# FN-BMD(no BP/BP)

no closed loops

# Clinical efficacy

Treatments used

AA (reference): ACE

AB: Acu

AC: BP

AD: BP_ACE

AE: CT_VD

AF: CT_VD_ACE

AG: CT_VD_Acu

AH: CT_VD_BP

AI: CT_VD_BP_Acu

AJ: CT_VD_BP_Acu_Mox

AK: CT_VD_BP_Acu_Pat

AL: CT_VD_BP_Mox

AM: CT_VD_BP_Pat

AN: CT_VD_BP_TCM_ACE

AO: CT_VD_BP_TCM_Acu

AP: CT_VD_DuMox

AQ: CT_VD_Mox

AR: CT_VD_TCM

AS: CT_VD_TCM_Acu

AT: CT_VD_TFMox

AU: CT_VD_WarmAcu

AV: E

AW: TCM

AX: TCM_ACE

AY: TCM_Acu

AZ: TCM_FNeedle

BA: TCM_WarmAcu

BB: WarmAcu

# TCM syndrome score

Treatments used

A (reference): CT_VD

B: CT_VD_ACE

C: CT_VD_Acu

D: CT_VD_BP

E: CT_VD_BP_Acu

F: CT_VD_BP_Acu_DuMox

G: CT_VD_BP_Mox

H: CT_VD_BP_Pat

I: CT_VD_BP_TCM_Acu

J: CT_VD_DuMox

K: CT_VD_Mox

L: CT_VD_ShortPri

M: CT_VD_TCM

N: CT_VD_TCM_Acu

O: CT_VD_TCM_FDMox

P: CT_VD_TFMox

# TCM low back pain(exclude BP)

A (reference): CT_VD

B: CT_VD_Mox

C: CT_VD_Pat

D: CT_VD_TCM

E: CT_VD_TCM_Acu

F: CT_VD_TCM_Mox

G: CT_VD_TFMox

H: CT_VD_WarmAcu

I: TCM

J: TCM_Acu

include BP: no closed loops

# VAS score

AA (reference): ACE

AB: CT_VD

AC: CT_VD_ACE

AD: CT_VD_Acu

AE: CT_VD_BP

AF: CT_VD_BP_Acu

AG: CT_VD_BP_Acu_Mox

AH: CT_VD_BP_Acu_Pat

AI: CT_VD_BP_Pat

AJ: CT_VD_BP_TCM_Acu

AK: CT_VD_ChenpiDuMox

AL: CT_VD_DuMox

AM: CT_VD_MNKnife_Mox

AN: CT_VD_Mox

AO: CT_VD_ShortPri

AP: CT_VD_TCM

AQ: CT_VD_TCM_Acu

AR: CT_VD_TFMox

AS: CT_VD_WarmAcu

AT: Cel

AU: DuMox

AV: E

AW: TCM

AX: TCM_ACE

AY: TCM_Acu

AZ: TCM_DuMox

BA: TCM_FNeedle

BB: TCM_Mox

BC: TCM_WarmAcu

# PINP

no closed loops

# CTX

Treatments used

A (reference): CT_VD

B: CT_VD_Acu

C: CT_VD_BP

D: CT_VD_BP_Acu

E: CT_VD_BP_AcuInj

F: CT_VD_BP_Acu_DuMox

G: CT_VD_BP_Acu_Mox

H: CT_VD_BP_Acu_Pat

I: CT_VD_BP_Mox

J: CT_VD_BP_Pat

K: CT_VD_BP_TCM_Acu

L: CT_VD_BP_TCM_Acu_Mox

M: CT_VD_BP_TFMox

N: CT_VD_TCM

O: CT_VD_TCM_Acu

P: CT_VD_TFMox

Note: Loop B-N-O is formed only by multi-arm trial(s) - Consistent by definition

# E2

Treatments used

A (reference): CT_VD

B: CT_VD_ACE

C: CT_VD_Acu

D: CT_VD_BP

E: CT_VD_BP_Acu

F: CT_VD_BP_Mox

G: CT_VD_BP_Pat

H: CT_VD_BP_TCM_ACE

I: CT_VD_TCM

J: CT_VD_WarmAcu

# ALP（不含BP）

A (reference): ACE

B: Acu

C: CT_VD

D: CT_VD_Mox

E: CT_VD_TCM

F: CT_VD_TCM_Acu

G: CT_VD_TCM_Mox

H: E

I: TCM

J: TCM_ACE

K: TCM_Acu

Dbar pD DIC

18.33276 18.10921 36.44197

18 data points, ratio 1.018, I^2 = 7%

Dbar pD DIC

17.97869 17.97834 35.95704

18 data points, ratio 0.9988, I^2 = 5%

# ALP（含BP）

Dbar pD DIC

18.84719 18.08061 36.92780

18 data points, ratio 1.047, I^2 = 10%

Dbar pD DIC

18.80187 18.08977 36.89164

18 data points, ratio 1.045, I^2 = 10%

No close loops

# OCN (no bp)

Treatments used

A (reference): CT_VD

B: CT_VD_Acu

C: CT_VD_HSMox_ElecAcu

D: CT_VD_Mox

E: CT_VD_TCM

F: CT_VD_TCM_Acu

G: CT_VD_TFMox

H: CT_VD_WarmAcu

Note: Loop A-D-E is formed only by multi-arm trial(s) - Consistent by definition

# OCN(bp)

No closed loops
