# Supplementary material for: Evaluating combined acupuncture and antiresorptive therapy in Chinese women with postmenopausal osteoporosis: a systematic review and network meta-analysis
Source: Front Endocrinol (Lausanne). 2026 Jul 1;17:1784394. doi: 10.3389/fendo.2026.1784394 (PMC13368563; doi:10.3389/fendo.2026.1784394)
Supplement: Supplementary file 7 [file DataSheet7.docx]

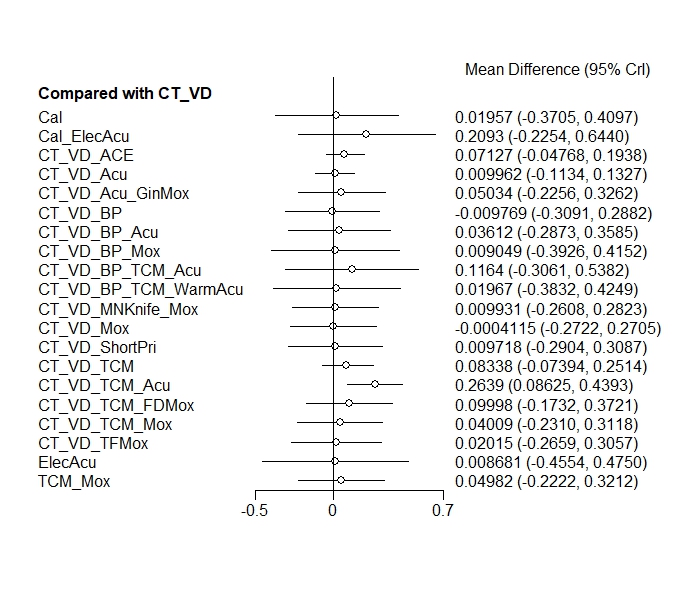


Figure S2. 1 The forest plot for LS-BMD


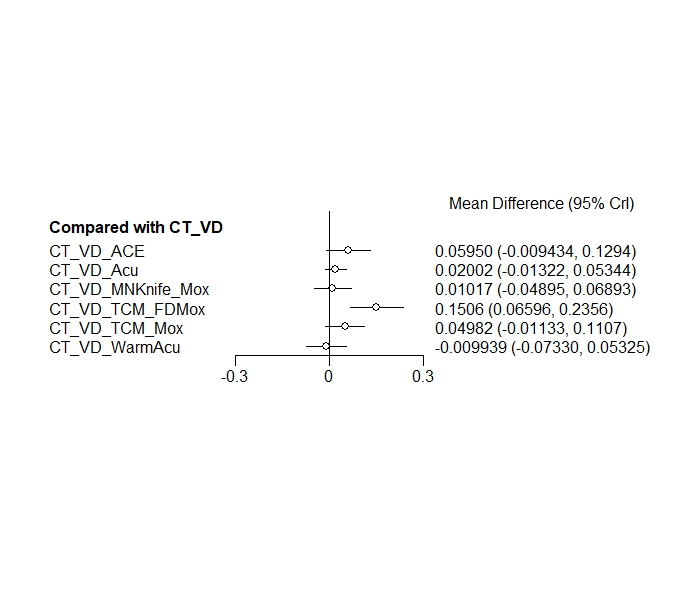


Figure S2. 2 The forest plot for the BP-free FN-BMD


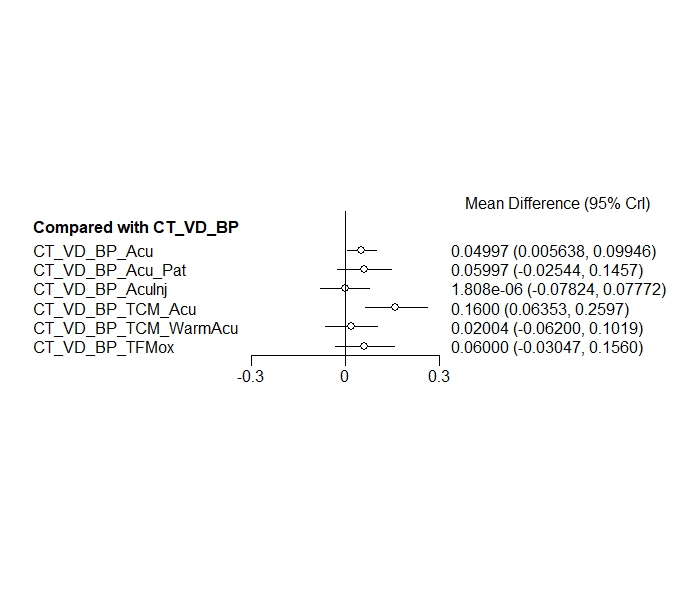


Figure S2. 3 The forest plot for the BP-containing FN-BMD


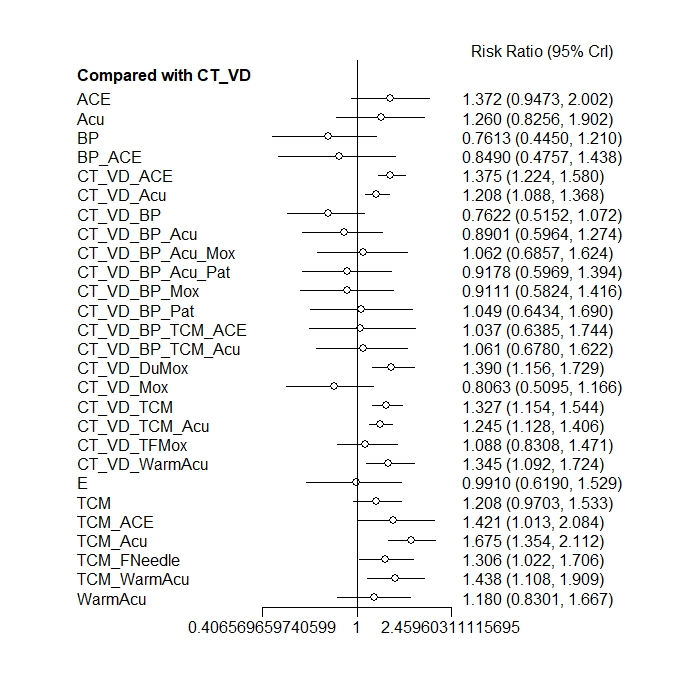


Figure S2. 4 The forest plot for clinical efficacy


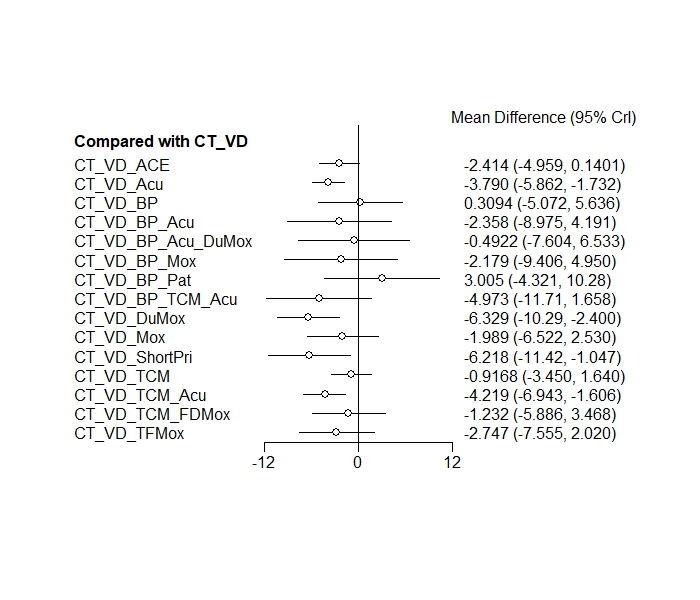


Figure S2. 5 The forest plot for the total TCM syndrome score


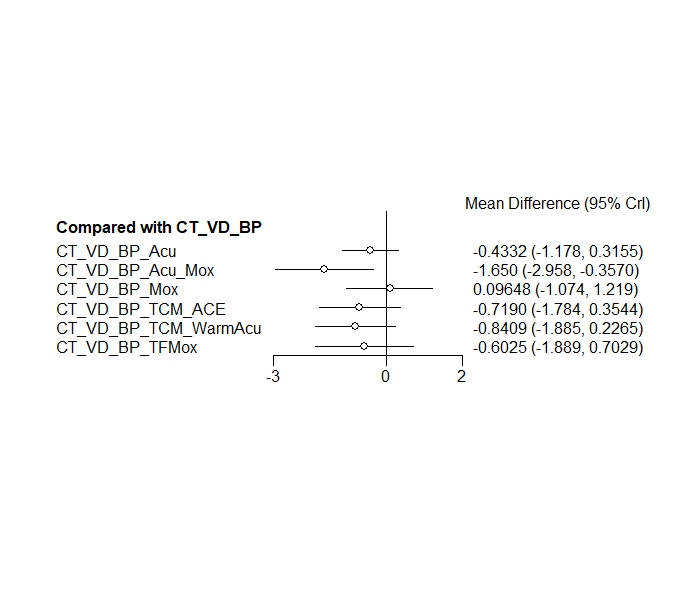


Figure S2. 6 The forest plot for low back pain score of TCM syndrome score (include BP)


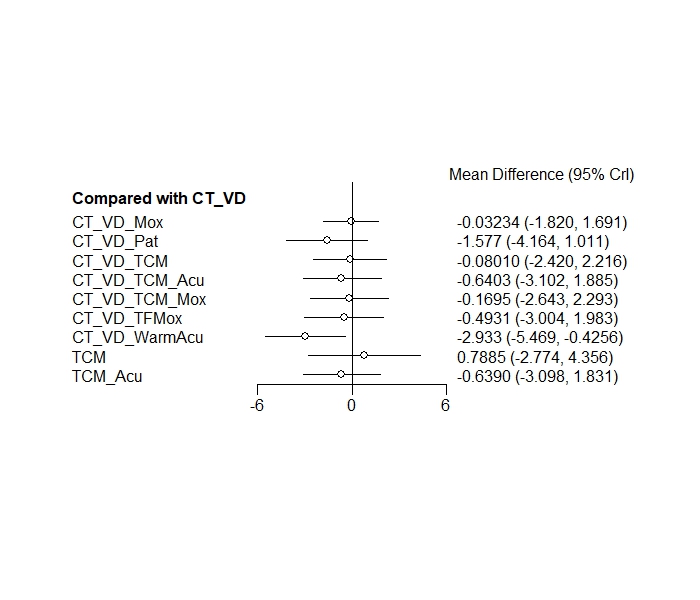


Figure S2. 7 The forest plot for low back pain score of TCM syndrome score (exclude BP)


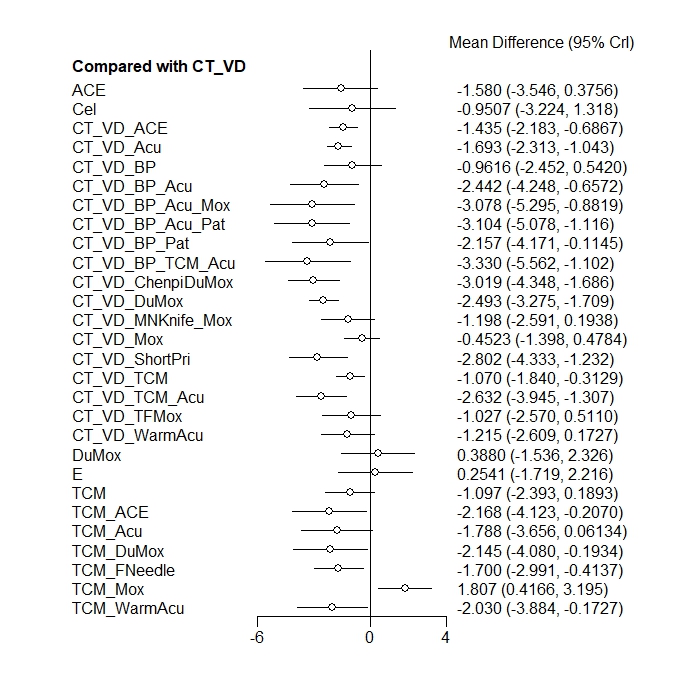


Figure S2. 8 The forest plot for the VAS score


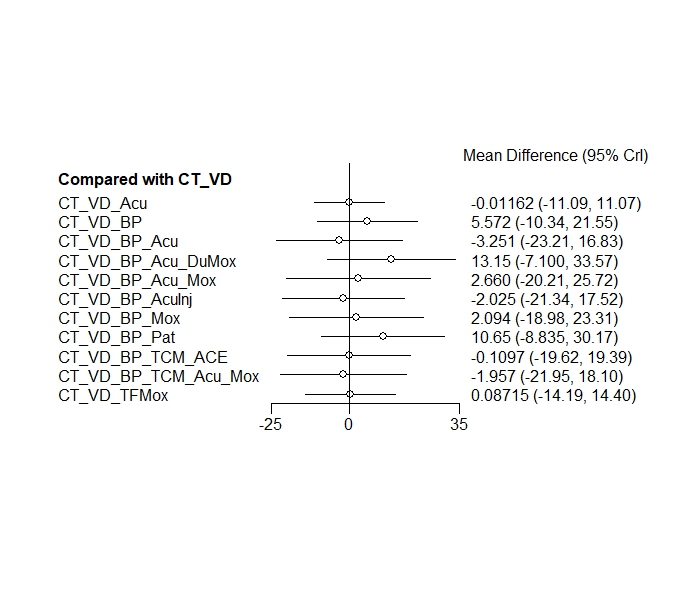


Figure S2. 9 The forest plot for PINP


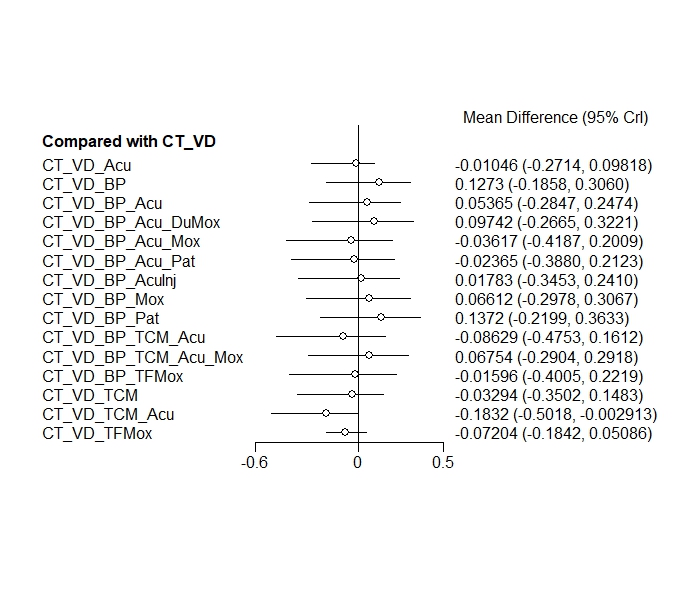


Figure S2. 10 The forest plot for CTX


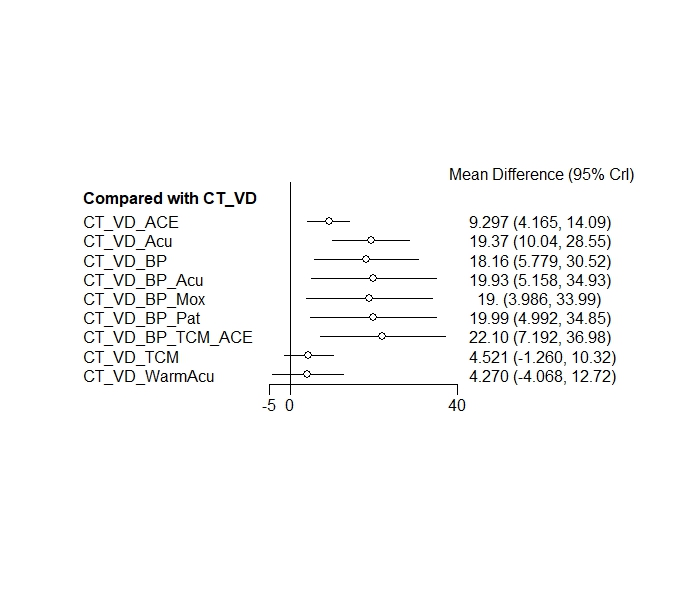


Figure S2. 11 The forest plot for E2


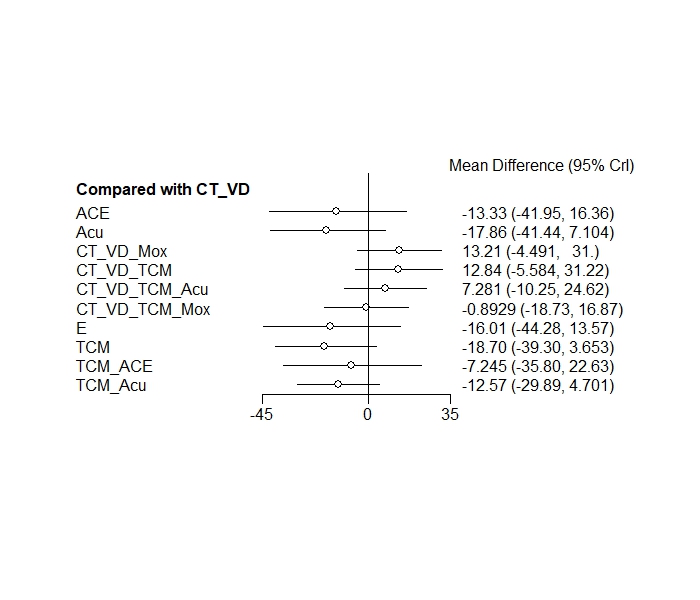


Figure S2. 12 The forest plot for ALP (exclude BP)


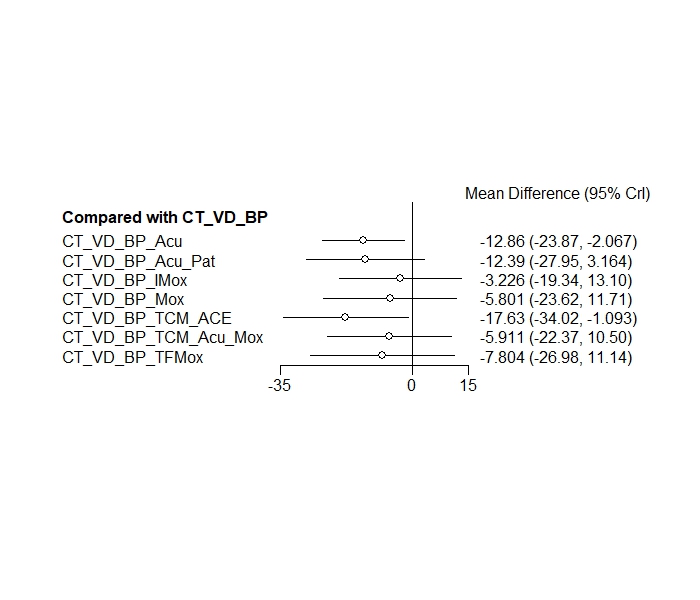


Figure S2. 13 The forest plot for ALP (include BP)


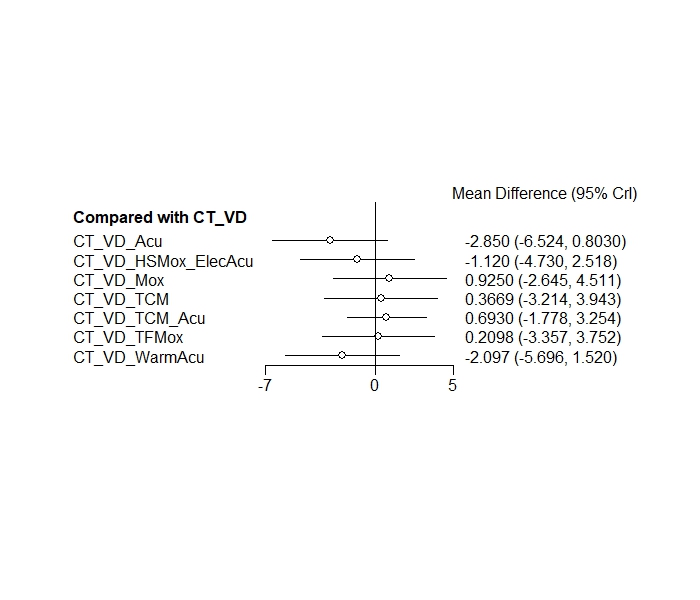


Figure S2. 14 The forest plot for OCN (exclude BP)


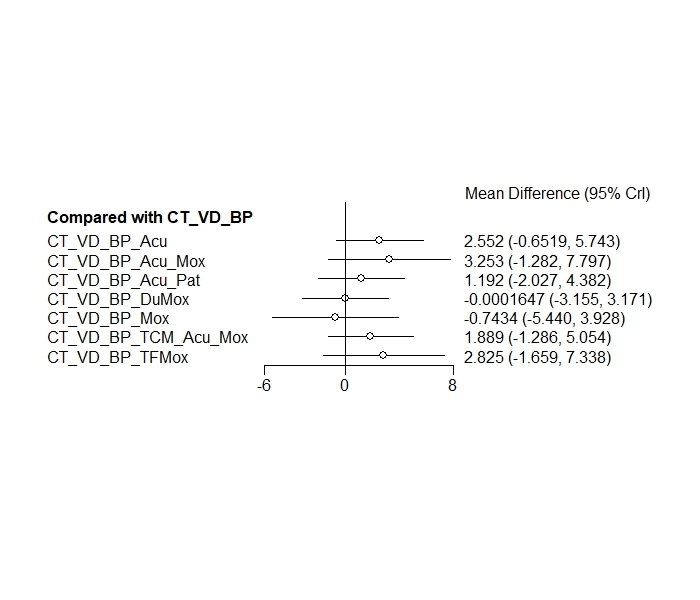


Figure S2. 15 The forest plot for OCN (include BP)
